# Current status of molecular rice breeding for durable and broad-spectrum resistance to major diseases and insect pests

**DOI:** 10.1007/s00122-024-04729-3

**Published:** 2024-09-10

**Authors:** Xiaoyan Cheng, Guohua Zhou, Wei Chen, Lin Tan, Qishi Long, Fusheng Cui, Lei Tan, Guoxing Zou, Yong Tan

**Affiliations:** 1 Jiangxi Tiandao Liangan Seed Industry Co., Ltd., 568 South Huancheng Rd., Yuanzhou Dist., Yichun, People’s Republic of China; 2 National Engineering Research Center of Rice (Nanchang), Rice Research Institute, Jiangxi Academy of Agricultural Sciences, Nanchang, People’s Republic of China; 3 College of Life Sciences and Resources and Environment, Yichun University, Yichun, People’s Republic of China; 4 Jiangxi Super-Rice Research and Development Center, Jiangxi Provincial Key Laboratory of Rice Germplasm Innovation and Breeding, Jiangxi Academy of Agricultural Sciences, National Engineering Research Center for Rice, Nanchang, People’s Republic of China; 5 Yichun Academy of Sciences (Jiangxi Selenium-Rich Industry Research Institute), Yichun, People’s Republic of China

## Abstract

In the past century, there have been great achievements in identifying resistance (*R*) genes and quantitative trait loci (QTLs) as well as revealing the corresponding molecular mechanisms for resistance in rice to major diseases and insect pests. The introgression of *R* genes to develop resistant rice cultivars has become the most effective and eco-friendly method to control pathogens/insects at present. However, little attention has been paid to durable and broad-spectrum resistance, which determines the real applicability of *R* genes. Here, we summarize all the *R* genes and QTLs conferring durable and broad-spectrum resistance in rice to fungal blast, bacterial leaf blight (BLB), and the brown planthopper (BPH) in molecular breeding. We discuss the molecular mechanisms and feasible methods of improving durable and broad-spectrum resistance to blast, BLB, and BPH. We will particularly focus on pyramiding multiple *R* genes or QTLs as the most useful method to improve durability and broaden the disease/insect spectrum in practical breeding regardless of its uncertainty. We believe that this review provides useful information for scientists and breeders in rice breeding for multiple stress resistance in the future.

**Supplementary Information:**

The online version contains supplementary material available at 10.1007/s00122-024-04729-3.

## Introduction

Rice has become one of the most important staple crops, feeding over half of the world’s population (Cheng et al. 2013a). Annually, up to 37% of rice yield is lost due to diseases and arthropod pests on average (Sparks et al. 2012). Among them, blast, BLB, and BPH are the three main biotic stresses (Ji et al. 2016). Over approximately 4 million tons of pesticides are used annually (FAO 2021) to control detrimental insects, fungal, and bacterial diseases. As a result, they have caused adverse effects on water, soil, air, and various taxa, which finally severely affect global ecosystem, biodiversity, and human health (Sharma et al. 2019).

The development of resistant cultivars by introducing resistance (*R*) genes into rice has become a sustainable and environmental-friendly approach for disease and insect management (Cheng et al. 2013a; Yin and Qiu 2019). A major limitation of host plant resistance is that targeted pest species can overcome resistance mediated by specific resistance genes by producing new virulent strains of the pathogens and insects (Palloix et al. 2009). For example, the resistance effects of the blast disease gene were overcome within 3 years after planting (Kiyosawa 1982). The pathogen and insect populations have such extremely rapid evolutionary speed, but the process of discovering a new *R* gene and incorporating it into a rice cultivar is quite slow and inefficient (Li et al. 2021b). Thus, durable resistance in cultivars has increasingly attracted attention since durable resistance can remain effective over a longer time (Johnson 1979, 1984). This descriptive term provides no implications for genetic background or molecular mechanism but only two empirical elements—time and area—which are difficult to define clearly for different farmers (Brown 2015). For a subsistence farmer, durable resistance is desired to maintain stable, predictable, and adequate crop production other than an above-average or maximizing yield, while a farmer in industrial agriculture prefers new crop cultivars with the most commercial value in the market to those that are out of date, which requires durable resistance to maintain crops with the highest yield and quality (Summers and Brown 2013; Brown 2015; Anuradha et al. 2022).Unfortunately, little is known about the molecular mechanisms for durable resistance in rice (Johnson 1993; Brown 2015).

Rice varieties with broad-spectrum resistance are highly desirable since this type of resistance is effective to more than one pathogen species or multiple races/strains of the same pathogen (Kou and Wang 2010; Hu et al. 2023). Many organizations and governments have accepted that the current norm for the public release of new rice varieties since these varieties have been evaluated for resistance to major diseases and insects based on standard protocols (Bank; Benedict and Linscombe 2009; IRRI, 2022). Recently, the term broad-spectrum resistance has expanded to include resistance to at least two different insect species or multiple biotypes/populations of the same insect pest (Jairin et al. 2007a; Liu et al. 2015; Kloth et al. 2021). The genetic basis for broad-spectrum resistance may include membrane-associated pattern recognition receptors (PRRs) and R proteins in the plant innate immunity system, defense-signaling proteins, pathogenesis-related (PR) proteins, susceptibility (S) proteins, quantitative trait loci (QTLs), and non-host resistance (NHR) (Liu et al. 2015; Fonseca and Mysore 2019; Li et al. 2020a). Both broad-spectrum and durable resistance have become increasingly prominent for practical applications since their combination provides an effective strategy for better pest managements to avoid heavy economic losses.

Another constraint on breeding for disease and insect resistance in rice for commercial crop varieties is the pleiotropic effects of resistance, leading to a growth–defense trade-off (Huot et al. 2014). In most cases, plant defense responses to pathogens and insects can evoke fitness costs associated with crop growth and development, the valuable agronomic property of the cultivar, and resistance to other environmental stresses, which lead to economic losses in yield and/or cooking and taste quality and unpredictable ecological consequences (Brown and Rant 2013; Huot et al. 2014; Wiesner-Hanks and Nelson 2016; Nelson et al. 2018). Therefore, farmers prefer to growing rice varieties with higher yields and better quality instead of growing cultivars with pest resistance but lower commercial values even though those varieties with higher economic values need to use chemical pesticides for pest control. During the post-Green Revolution era, many elite rice cultivars were developed with high yield and superior quality. Hence, those *R* genes that would not alter yield-associated traits and superior grain quality of the elite rice cultivars have been used in breeding programs with the aim of obtaining the most benefits of resistance against diseases and pests.

Marker-assisted selection (MAS) refers to the indirect selection process of target traits according to a DNA marker linked to a desired trait. Since it was first used in tomato breeding four decades ago (Tanksley and Rick 1980; Soller and Beckmann 1983), it has made a profound impact on crop genetic improvement by manipulating non-observable traits in conventional breeding. It has accelerated resistance breeding for diseases and pests, tolerance breeding for drought and hypersalinity, and the introgression of important agronomic traits, such as plant architecture and seed morphology (Ben-Ari and Lavi 2012). The extensive use of MAS has even remodeled the general procedures of conventional breeding, such as backcrossing, pyramiding, and recurrent selection, to marker-assisted backcrossing, marker-assisted gene pyramiding (MAGP), and marker-assisted recurrent selection. Thus far, it has become a powerful new methodology in crop breeding (Jiang 2015). In particular, MAGP of multiple *R* genes and/or QTLs in crop resistance breeding has empirical applications in improving durability and broad-spectrum resistance against disease and insects (Mekonnen et al. 2017; Fukuoka 2018).

For the past thirty years, there has been an increasing number of reports about *R* genes and/or QTLs identified from germplasm resistant lines with good resistance against diseases and insects, and in some cases, molecular mechanisms underlying rice defense against biotic stress have been studied and their potential applications in practical breeding have been explored. However, little attention has been paid to durable and broad-spectrum resistance to diseases and insects, which are really two most important parameters to determine the practical values in breeding programs and rice production. Here, we summarize recent significant progress on durable and broad-spectrum resistance to blast, leaf blight, and brown planthopper in rice. We focus on the genetic basis and feasible methods for improving durable and broad-spectrum resistance to blast, BLB, and BPH in practical breeding. Additionally, our work provides the primary basis of molecular design for breeding for multiple stress resistance in the future.

## Durable and broad-spectrum resistance to rice blast disease

Rice blast disease, caused by the phytopathogenic fungus *Magnaporthe oryzae* (anamorph: *Pyricularia grisea*), is one of the most devastating rice diseases in the world. Because this fungal disease leads to annual losses of 10–30% of the global rice yield with an estimated cost of about $ 66 billion (Pennisi 2010; Boddy 2016). It uses an appressorium to penetrate the plant cell wall and then manipulates host cells for parasitism (Galhano and Talbot 2011). The pathogen attacks all aboveground parts of a rice plant, including the leaf, collar, node, neck, panicle, and leaf sheath in the field and even roots in the laboratory (Sesma and Osbourn 2004; Marcel et al. 2010).

The rice blast pathosystem follows a typical gene-for-gene model of plant–microbe interactions, showing that the avirulence (*AVR*) gene from *M*. *oryzae* triggers resistance responses in the host rice harboring the corresponding *R* gene (Wang et al. 2017a). More than 100 blast resistance genes/alleles and 500 related QTLs have been identified (Li et al. 2019a; Dixit et al. 2020), most of which have been identified by molecular and QTL mapping (Tanweer et al. 2015). Primarily, these approaches are built on rice germplasm identified as donors of blast resistance in laboratory or field work. Considering the potentially applicable values of the improvement of rice varieties, the genes/loci conferring broad-spectrum resistance have attracted a great deal of attention. Thus far, approximately 44 genes/loci with broad-spectrum resistance have been identified (Table 1 and Supplemental Table 1), and 38 have been used for MAS in rice breeding programs (Supplemental Table 2) (Rao et al. 2014; Junjie et al. 2019; Mehta et al. 2019). Among the identified genes, *Pi1*, *Pi2/Piz-5*, *Pi9*, *pi21*, *Pi54/Pi-kh*, *Pb1*, and *Pigm* have been widely used in breeding programs. The genes *Pi1*, *Pi9*, *pi21*, *Pb1*, and *Pigm* confer broad-spectrum and durable resistance to rice blast (Fukuoka et al. 2009; Hayashi et al. 2010; Hua et al. 2012; Rathour et al. 2016; Deng et al. 2017). *Pb1* has been widely used in Japan for over 40 years and still remains resistance to blast in fields (Fujii et al. 2023). As shown in Supplemental Table 2, only one or two *Pi* genes have been more successfully introduced into rice varieties/lines to improve blast resistance. Pyramiding multiple genes (three or more *Pi*) remains to be developed.

**Table 1:** Identified *R* genes and QTLs in rice conferring broad-spectrum and durable resistance to fungal blast, bacterial leaf blight (BLB), and brown planthopper (BPH)

	Gene/Locus	Broad-spectrum Resistance	Durable resistance	References
Blast	*Pi1*	+	+	Hua et al. (2012)
	*Pi2/Piz-5*	+	ND	Liu et al. (2002)
	*Pi3/Pi5*	+	ND	Jeon et al. (2003)
	*Pi7(t)*	+	ND	Wang et al. (1994), Telebanco-Yanoria et al. (2010)
	*Pi9*	+	+	Liu et al. (2002), Qu et al. (2006)
	*pi21*	+	+	Fukuoka and Okuno (2001), Fukuoka et al. (2009)
	*Pi25*	+	+	Wu et al. (2005), Chen et al. (2011a, b)
	*Pi33*	+	ND	Berruyer et al. (2003), Ballini et al. (2007)
	*Pi35*	+	ND	Fukuoka et al. (2014)
	*Pi39*	+	ND	Hua et al. (2015)
	*Pi40*	+	+	Jena et al. (2007), Jeung et al. (2007)
	*Pi41*	+	ND	Yang et al. (2009)
	*Pi46*	+	ND	Xiao et al. (2011)
	*Pi47*	+	ND	Huang et al. (2011)
	*Pi48*	+	ND	Huang et al. (2011)
	*Pi49*	+	+	Sun et al. (2013)
	*Pi50*	+	ND	Zhu et al. (2012), Su et al. (2015)
	*Pi54/Pi-kh*	+	ND	Sharma et al. (2005)
	*Pi56(t)*	+	ND	Liu et al. (2013)
	*Pi57(t)*	+	ND	Dong et al. (2017)
	*Pi63*	+	ND	Xu et al. (2014a)
	*Pi64*	+	ND	Ma et al. (2015)
	*Pb1*	+	+	Hayashi et al. (2010)
	*Pia*	+	ND	Telebanco-Yanoria et al. (2010), Zeng et al. (2011)
	*Pib*	+	ND	Wang et al. (1999), Telebanco-Yanoria et al. (2010)
	*Pid3*	+	ND	Shang et al. (2009), Xu et al. (2014b)
	*Pi-d(t)*	+	ND	Chen et al. (2004)
	*Pii*	+	ND	Suh et al. (2009), Takagi et al. (2013)
	*Pik*	+	+	Telebanco-Yanoria et al. (2010), Zhai et al. (2011)
	*Pik-m*	+	ND	Ashikawa et al. (2008)
	*Pik-p*	+	ND	Yuan et al. (2011)
	*Pik-s*	+	ND	Wang et al. (2009a, b)
	*Pish*	+	+	Koide et al. (2010)
	*Pit*	+	ND	Hayashi and Yoshida, (2009)
	*Pita*	+	ND	Bryan et al. (2000), Telebanco-Yanoria et al. (2010)
	*Pita2/Ptr*	+	ND	Meng et al. (2020)
	*Piz*	+	ND	Suh et al. (2009)
	*Piz-t*	+	ND	Zhou et al. (2006)
	*Pizh*	+	+	Xie et al. (2019)
	*Pigm*	+	+	Deng et al. (2017)
	*qBl1/QTL1*	+	ND	Sreewongchai et al. (2010), Wongsaprom et al. (2010)
	*qBI11QTL11*	+	ND	Sreewongchai et al. (2010), Wongsaprom et al. (2010)
	*QTL2*	+	ND	Sreewongchai et al. (2010)
	*QTL12*	+	ND	Sreewongchai et al. (2010)
BLB	*Xa2*	+	ND	He et al. (2006), Ji et al. (2020)
	*Xa3/Xa26*	+	+	Kaku and Ogawa, (2000), Kaku and Ogawa, (2001), Sun et al. (2004), Xiang et al. (2006), Deng et al. (2018)
	*Xa4*	+	+	Wang et al. (2001), Hu et al. (2017)
	*xa5*	+	ND	Li et al. (2001), Iyer and McCouch (2004)
	*Xa6/Xa3*	+	ND	Sidhu and Khush (1978), Kaku and Ogawa (2000)
	*Xa7*	+	+	Vera Cruz et al. (2000), Porter et al. (2003), Chen et al. (2021)
	*xa8*	+	ND	Vikal et al. (2014)
	*xa9*	+	ND	Singh et al. (1983), Hisatoshi, (1993), Yogesh and Dharminder, (2017)
	*Xa10*	+	ND	Gu et al. (2008)
	*Xa11*	+	ND	Goto et al. (2009)
	*xa13*	+	ND	Li et al. (2001), Chu et al. (2006)
	*xa15*	+	ND	Nakai et al. (1988)
	*Xa18*	+	ND	Yamamoto and Ogawa, (1990)
	*xa19*	+	ND	Taura et al. (1991), Taura and Ichitani, (2023)
	*xa20*	+	ND	Taura et al. (1992), Msami et al. (2021)
	*Xa21*	+	ND	Khush et al. (1990), Ronald et al. (1992), Song et al. (1995)
	*Xa22(t)*	+	ND	Lin et al. (1996), Wang et al. (2003)
	*Xa23*	+	ND	Zhang et al. (2001), Wang et al. (2014), Wang et al. (2015)
	*xa24*	+	ND	Wu et al. (2008)
	*Xa25*	+	ND	Gao et al. (2002), Gao et al. (2005)
	*xa26(t)*	+	ND	Lee et al. (2003)
	*Xa27(t)*	+	ND	Gu et al. (2004)
	*xa28(t)*	+	ND	Lee et al. (2003)
	*Xa30(t)*	+	ND	Wang et al. (2004), Jin et al. (2007)
	*Xa31(t)*	+	ND	Wang et al. (2009a, b), Ji et al. (2020)
	*Xa32(t)*	+	ND	Zheng et al. (2009)
	*Xa33*	+	ND	Kumar et al. (2012)
	*xa33(t)*	+	ND	Korinsak et al. (2009)
	*Xa34(t)*	+	ND	Ram et al. (2010)
	*xa34(t)*	+	ND	Chen et al. (2011a, b)
	*Xa35(t)*	+	ND	Guo et al. (2010)
	*Xa36(t)*	+	ND	Miao et al. (2010)
	*Xa38*	+	ND	Vikal et al. (2007), Cheema et al. (2008)
	*Xa39*	+	ND	Zhang et al. (2015)
	*Xa40(t)*	+	ND	Kim et al. (2015)
	*xa41(t)*	+	ND	Hutin et al. (2015)
	*xa42*	+	ND	Busungu et al. (2016), Busungu et al. (2018)
	*xa42(t)*	+	ND	Zeng et al. (2009), Liang et al. (2017)
	*Xa43(t)*	+	ND	Kim (2018), Kim and Reinke (2019)
	*xa44(t)*	+	ND	Kim (2018)
	*Xa45(t)*	+	ND	Ji et al. (2020)
	*xa45(t)*	+	ND	Neelam et al. (2020)
	*Xa46(t)*	+	ND	Chen et al. (2020)
	*Xa47*	+	+	Xing et al. (2021), Lu et al. (2022)
BPH	*BPH1*	+	ND	Khush and Brar (1991), Jena and Kim (2010)
	*bph2*	+	ND	Khush and Brar (1991), Young-Soon et al. (2008), Jena and Kim (2010)
	*BPH3*	+	ND	Khush and Brar (1991), Jairin et al. (2007a), Jena and Kim (2010), Liu et al. (2015)
	*bph4*	+	ND	Khush and Brar (1991), Jena and Kim (2010)
	*BPH6*	+	ND	Khush and Brar (1991), Jena and Kim (2010), Guo et al. (2018)
	*bph7*	+	ND	Khush and Brar (1991), Jena and Kim (2010), Qiu et al. (2014)
	*bph8*	+	ND	Khush and Brar (1991), Jena and Kim (2010)
	*BPH9*	+	ND	Murata et al. (2001), Zhao et al. (2016)
	*BPH9*	+	ND	Khush and Brar (1991), Jena and Kim (2010)
	*BPH10*	+	ND	Ishii et al. (1994)
	*BPH17*	+	ND	Sun et al. (2005)
	*BPH18*	+	ND	Jena and Kim (2010)
	*bph18(t)*	+	ND	Li et al. (2006)
	*bph19(t)*	+	ND	Li et al. (2006)
	*bph22(t)*	+	ND	Hou et al. (2011)
	*bph23(t)*	+	ND	Hou et al. (2011)
	*bph25*	+	ND	Myint et al. (2012)
	*BPH30*	+	ND	Shi et al. (2021)
	*BPH31*	+	ND	Prahalada et al. (2017)
	*BPH43*	+	ND	Kim et al. (2022)
	*qBPH6(t)*	+	ND	Jairin et al. (2007b)

ND: no data. Details on rice *R* genes/loci with broad-spectrum and durable resistance to blast, BLB, and BPH are listed in Supplemental Tables 1, 3 and 4, respectively

**Table 2:** Number of Chinese super rice cultivars and Indian high-yielding varieties with resistance to blast, bacterial leaf blight (BLB), and brown planthopper (BPH)

	Resistance to 1 stress			Resistance to 2 stresses			Resistance to Blast + BLB + BPH
	Blast	BLB	BPH	Blast + BLB	BLB + BPH	Blast + BPH	
China	31	8	1	2	0	0	0
India	45	15	4	13	2	5	4

Details on Chinese super rice admitted by the Ministry of Agriculture and Rural Affairs by 2020 are shown in Supplemental Table 6. Data were collected from the China Rice Data Center (https://ricedata.cn). Details on Indian high-yielding rice varieties, which were developed by the Indian Council of Agricultural Research-National Rice Research Institute (ICAR-NRRI) and released by the Central Varietal Release Committee (CVRC), are included in Supplemental Table 7. Data were collected from the National Rice Research Institute (https://icar-nrri.in/released-varieties/)

Defense regulating genes, genes involved in NHR, and QTLs related to blast resistance are important keys to engineering durable resistance in crops (Fonseca and Mysore 2019; Li et al. 2019a). Seventy-seven defense regulating genes have been extensively studied regarding the mechanisms underlying rice responses to blast pathogens (Li et al. 2019a). *bsr-d1*, *spl11*, *spl33*, *OsBBI1*, *OsMYB30*, *OsNAC60*, *PIBP1*, and *LHCB5* confer broad-spectrum resistance to the rice blast disease (Zeng et al. 2004; Li et al. 2011, 2017, 2020b; Wang et al. 2017b, 2018; Liu et al. 2019; Zhai et al. 2019). In particular, *bsr-d1* shows broad-spectrum blast resistance without a significant yield penalty (Li et al. 2017). However, there are rare examples of their practical utilization in breeding programs.

Diverse genes are involved in NHR, such as physical or chemical barriers, cell death, reactive oxygen species (ROS) accumulation, lignification, and callose deposition. Thus far, many attempts have been made to introduce or overexpress NHR genes from other plant species into crops (Fonseca and Mysore 2019). In particular, overexpression of *NPR1* from Arabidopsis and *RXO1* from maize in rice has provided valuable information to engineer ideal broad-spectrum disease resistance in the laboratory (Zhao et al. 2005; Xu et al. 2017). However, to date, the effective utilization of NHR genes in practical applications is still very limited.

QTLs usually improve plant resistance partially, which shows smaller additive effects than major *R* genes. However, QTL-mediated resistance is usually non-race-specific. Thus, weak but additive resistance mediated by QTLs is considered a potential alternative to engineer durable resistance against a broad range of pathogens (Fukuoka et al. 2015; Srivastava et al. 2017). Although less studied than *R* genes, four QTLs, *pi21*, *Pi35*, *Pi63*, and *Pb1*, have been cloned (Fukuoka et al. 2009, 2014; Hayashi et al. 2010; Xu et al. 2014a). QTLs can confer durable resistance in practical utilization (Fujii et al. 2023), but the allele alone is probably not able to control the disease under high disease pressure or in a complex environment. Therefore, pyramiding appropriate R genes together with QTLs may provide an applicable means to achieve durable and broad-spectrum resistance in the field.

Pyramided *Pi* genes are considered an artificially effective way to increase the blast resistance spectrum and durability (Liu et al. 2008; Luo et al. 2017; Guan et al. 2019). However, this method always leads to unexpected resistant effects with positive or negative deviations (Ning et al. 2020). Therefore, it seems greatly challenging for rice breeders to deploy and utilize such a high number of *Pi* genes to improve durable and broad-spectrum resistance. There are three major concerns in current resistance breeding programs. First, the genetic background usually alters the resistance spectrum and durability conferred by *R* genes (Cao et al. 2007; Gallois et al. 2018), which makes *R* genes with optimistic molecular demonstrations in laboratories often fall short in the field tests after being used in rice breeding (Brutus and Yang He 2010). Second, durable and broad-spectrum resistance to blast disease conferred by *R* genes in rice is inevitably at the expense of yield, grain quality, cooking and eating quality, or resistance to other abiotic stresses. This conflict propels breeders and farmers to find a balance between disease resistance and fitness costs according to their own experience and economic benefits. This is subjective and debatable, as it is not determined by an index but rather personal preference. Thus, a certain balance is difficult to apply extensively in rice breeding and production. Third, *R* gene frequency is diversified in different rice-planting regions, and the core effective *R* genes in rice varieties show obvious differences in different rice-growing zones. For example, the gene frequencies of *Pi9* were recorded as 50%, 21.4%, and 15.22% in Thai, Korea, and India, respectively. Thus, *Pi9* is the core effective *R* gene in Thai, but not in Korea or India. The *Pit* gene is the core effective *R* gene with a 60.24% gene frequency in India but cannot be detected in Korea (Cho et al. 2007; Yadav et al. 2019; Sooklim et al. 2022). However, different ecological zones can share similar key effective *R* genes. For example, Guangdong province (20°13′–25°31′N, 109°39′–117°19′E) in China is characterized by high ambient temperatures, and Hunan province (24°37′–30° 08′N, 108°47′–114°16′E) has a high summer and low fall temperature. However, both provinces share the same key effective *R* genes, *Pi1*, *Pik*, *Pik-m*, and *Piz* (Zhang et al. 2017). In addition, similar ecological zones have different key effective genes, such as Jiangsu province in China with *Pish*, *Pit*, and *Pia* (Qi et al. 2023) and Jiangxi province with *Piz* and *Pid3* (Lan et al. 2019). These factors may constrain the improvement of durable and broad-spectrum resistance to rice blast disease in a limited region and a short time.

There are other approaches to improving durable and broad-spectrum resistance to blast disease. Due to the rapid development of CRISPR/Cas9‐mediated genome editing, it is feasible to knock out disease-susceptibility (*S*) genes (sometime recessive *R* genes) or related disease-susceptibility factors to generate new varieties with broad-spectrum resistance to blast disease, e.g., *pi21* and *RESISTANCE TO BLAST1* (*RBL1*) (Nawaz et al. 2020; Tao et al. 2021; Sha et al. 2023). However, the authority’s approval is required before the commercialization of crops and foods created using genome editing technology, which is always debatable and takes a lot of time. Another strategy for the long-term control of blast pathogens is rice multilines, which are made of several near-isogenic lines (NILs) with different *R* genes showing uniform phenotypic agricultural traits to reduce blast pathogen development and prevent the breakdown of blast *R* genes (Koizumi et al. 2004). Rice multilines have shown stable blast disease control in Japan for more than 10 years (Koizumi and Tani 2000; Ishikawa et al. 2022). However, the breeding of multiple NILs and strict maintenance increases the cost of developing and growing rice multilines. In addition, breeders need to pay continuous attention to the race distribution of the blast fungus (Fujii et al. 2023). Therefore, rice multilines controlling blast disease have not been accepted by farmers outside Japan.

## Durable and broad-spectrum resistance to rice blight disease

Rice blight disease, the most destructive bacterial disease among rice and rice-related species (Jiang et al. 2023; Savary et al. 2019; Sanya et al. 2022), leading to yield losses varying from 2 to 74% (Reddy 1979; Naqvi et al. 2018), is caused by the gram-negative and non-spore-forming bacteria *Xanthomonas oryzae* pv. *oryzae* (*Xoo*) (Ming et al. 1991; Ishiyama 1992). The disease is also called bacterial leaf blight (BLB) since it induces yellowing and drying of leaves, as well as wilting of seedlings (also called kresek). The pathogen enters rice tissues through wounds on leaves and roots and natural openings, such as hydathodes, stomata, and growth cracks caused by the emergence of new roots at the base of the leaf sheath; it multiplies in epithem cells, moves to the xylem vessels, and blocks the phloem (Gnanamanickam et al. 1999; Shekhar et al. 2020). The pathogen infects rice at all growth stages, resulting in severe losses. Infection at early growth stages can lead to yield losses of up to 90% (Shekhar et al. 2020). Infection at the later booting stage does not decrease the yield too much but leads to poor-quality grains and a high proportion of broken kernels (Niones et al. 2022). This disease was first observed in 1884–1885 in Japan, and the pathogen *Xoo* was identified in 1911. BLB is prevalent throughout rice-cropping regions, including Asia, Africa, Australia, Europe, Latin America, the Caribbean, and North America. This disease spreads easily through water, rain, typhoons, plant-to-plant contact, farm implements, and seeds (Niones et al. 2022).

The rice–*Xoo* pathosystem has been regarded as a genetic model for host–pathogen interactions and co-evolution (Niño-Liu et al. 2006; Chen et al. 2020). *R* gene-mediated BLB resistance usually follows gene-for-gene theory and is race-specific (Dai et al. 2007; Chen et al. 2020). To date, over 47 BLB resistance genes/loci (Supplemental Table 3) and 100 s of QTLs have been identified from cultivated and wild rice and artificial and natural mutants (Yang et al. 2021; Lu et al. 2022). Some genes confer resistance to a wide spectrum of *Xoo* races from different geographical locations, including *Xa2*, *Xa3/Xa26*, *xa5*, *Xa6/Xa3*, *Xa7*, *xa8*, *xa13*, *xa19*, *xa20*, *Xa21*, *Xa22(t)*, *Xa23*, *xa24*, *Xa25*, *Xa27(t)*, *Xa31(t)*, *Xa32(t)*, *Xa36(t)*, *Xa39*, *xa41(t)*, *Xa45(t)*, and *Xa47*, whereas others are effective against only one or a few strains that may be limited to a particular geographical location, such as *Xa1* and *Xa10* (Table 1 and Supplemental Table 3). Among these *R* genes, *Xa3/Xa26*, *Xa4*, *Xa7*, and *Xa47* have been reported to confer durable resistance against *Xoo* (Hu et al. 2017; Deng et al. 2018; Chen et al. 2021; Lu et al. 2022).

Thus far, 14 *Xa* genes (*Xa3/Xa26*, *Xa4*, *xa5*, *Xa7*, *Xa10*, *xa13*, *Xa21*, *Xa22*, *Xa23*, *Xa27*, *Xa33*, *xa33(t)*, *Xa38*, and *Xa40*) have been used in breeding practices (Fiyaz et al. 2022; Yang et al. 2022). *Xa4* is the most widely deployed BLB *R* gene in modern commercial rice varieties, including the famous ‘Green Revolution’ rice variety IR64, and has been deployed in 55 countries and over 80% of the total rice-farming area since the late 1960s (Khush et al. 1989; Khush 2005; Arif et al. 2010; Mackill and Khush 2018; Quibod et al. 2020). As a result, the regional *Xoo* race frequency has changed, especially the major population of *Xoo* races, and new strains virulent to *Xa4* have emerged and prevailed in different geographical locations, causing new BLB epidemics (Mew et al. 1992; Zhang 2009; Quibod et al. 2020). Therefore, seeking new genes with broad-spectrum and durable resistance and deploying these genes into rice varieties are important tasks for scientists and breeders. After the frequency of *Xa4* virulent outbreaks increased during the 1990s, *xa5*, *Xa7*, *xa13*, and *Xa21* were used in BLB resistance breeding throughout Asia (Furuya et al. 2012; Yugander et al. 2017; Chukwu et al. 2019; Quibod et al. 2020; Nugroho et al. 2022).

Many attempts have been made to pyramid multiple *R* genes and delay the breakdown of BLB resistance conferred by a single *R* gene. Thus far, two, three, four, and even five BLB *R* genes have been successfully pyramided in rice cultivars (Huang et al. 1997; Chukwu et al. 2019; Hsu et al. 2020). As the *Xoo* populations and *Xoo* pathotype distribution are continuously evolving with both local environments and modern agricultural practices, different *R* genes and pyramided *R* genes have been deployed in different counties in recent decades. For example, *Xa23* has been widely used in Chinese breeding programs over the past decade (Zhou et al. 2011; Zhang et al. 2015). While japonica rice cultivars carrying *Xa3/Xa26* have been widely grown in Korea (Shin et al. 2011). The gene combination of *xa5* and *xa13* confers resistance to all isolates throughout Vietnam (NODA et al. 1999), while the pyramided *xa5*, *xa13*, and *Xa21* are the most suitable gene combination applied in Indian BLB breeding programs (Mishra et al. 2013).

In addition to *R* genes and relative MAS breeding, there are other genes and up-to-date technologies that can potentially broaden the resistance spectrum of rice cultivars. For example, *S* genes to BLB have been engineered by CRISPR/Cas9-mediated gene editing to generate new rice lines with broad-spectrum resistance to *Xoo* races (Oliva et al. 2019; Xu et al. 2019). The genetic engineering of *Xa10* can enhance broad-spectrum and durable resistance to *Xoo* (Zeng et al. 2015). They provide novel strategies for creating new rice germplasm with broad-spectrum resistance, although further investigations to evaluate the performance of those edited lines in the field are still underway. Among the 44 defense regulator genes known to be involved in BLB resistance, 21 genes contribute to resistance to at least two *Xoo* isolates (Liu et al. 2021b). Compared with *R* genes and *S* genes, these genes have attracted much less attention in current molecular breeding. However, they enrich the gene pool for broad-spectrum resistance to disease, which might provide a new method for resistance breeding in the future.

At present, a new problem of BLB resistance breeding brings out as the new virulent pathogens causing bacterial leaf blight-like symptoms occur in different rice-growing regions. *Pantoea ananatis* was first reported as a new BLB pathogen, showing the same symptomatic rice leaves as *Xoo* in India (Mondal et al. 2011), Malaysia (Azizi et al. 2019), and the USA (Luna et al. 2023). In Korea, only *P*. *agglomerans* has been found (Lee et al. 2010). Both *P*. *ananatis* and *P*. *stewartii* have been discovered in Africa (Kini et al. 2017b, 2017a) and Thailand (Arayaskul et al. 2020). In Malaysia, both *P*. *ananatis* and *P*. *dispersa* have been discovered (Toh et al. 2019), and in China, both *P*. *ananatis* and *E*. *asburiae* have been reported (Xue et al. 2021). Specially, in southeast China, the BLB incidence caused by *P*. *ananatis* is 45–60%, which alarms the new present pathogen is replacing the traditional *Xoo* to cause disease and even a new BLB pandemic (Yu et al. 2022). Therefore, these new pathogens pose a great challenge to the rice cultivars incorporated with *Xa* genes, since we have not determined the occurrence, spread, and control of new emerging pathogens yet.

## Durable and broad-spectrum resistance to the brown planthopper

The brown planthopper, *Nilaparvata lugens* (Stål) (Hemiptera: Delphacidae, abbreviated as BPH), is the most devastating insect pest of rice in major rice producing countries. It can cause up to over 20% of yield loss and more than $ 300 million of economic loss every year, due to its high intrinsic fecundity, rapid development of insecticide resistance, and long-distance migration (Dyck and Thomas 1979; Stout 2014; Liu and Sun 2016; Muduli et al. 2021). It causes direct damage by feeding exclusively on rice sap from phloem tissues. The high population of BPHs in the field causes rice leaves to wilt and become brown, which is a phenomenon known as ‘hopperburn’. BPH can cause indirect damage by transmitting pathogens, such as grassy stunt virus and ragged stunt virus (Cheng et al. 2013a; Stout 2014).

Today, the deployment of the BPH resistance gene in rice cultivars has inevitably been considered the most effective and long-lasting method of BPH management, although it was seriously questioned at the beginning of the application of *BPH*-resistant varieties (Sogawa 2015). The first *BPH*-resistant variety carrying *Bph1*, IR26, defended against the prevalent BPH population biotype 1 and was released to the public in 1973 (Peng et al. 2000; Jena and Kim 2010). In the subsequent 3–4 years, BPH resistance was easily broken down as biotype 2 emerged and became predominant (Jena and Kim 2010). Variety IR36 harboring *bph2*, which confers resistance to biotype 2, was deployed widely for over 10 years after its release in 1976 (Peng et al. 2000; Guan et al. 2022). However, in 1981, biotype 3, which could overcome *bph2*-mediated resistance, was detected in the field (Zhao et al. 2016; Baehaki and Iswanto 2017). *Bph3* and *bph4* were discovered later and incorporated into rice cultivars with durable and broad-spectrum resistance to all BPH biotypes (Kawaguchi et al. 2001; Jairin et al. 2007a, 2010; Liu et al. 2015). This typical boom-and-bust cycle (Priestley 1978) forced scientists and breeders to explore new germplasm resources and new technologies that could confer durable and broad-spectrum resistance to rice cultivars.

Thus far, over 45 BPH resistance genes and genetic loci have been identified in wild and cultivated rice (Du et al. 2020; Sani Haliru et al. 2020). Among these, 21 genes/loci have been confirmed to confer broad-spectrum resistance (Table 1 and Supplemental Table 4). *BPH3*, *BPH6*, *bph25*, *BPH30*, and *qBPH6(t)* also exhibit resistance to another notorious pest, the white-backed planthopper (WBPH, *Sogatella furcifera*) (Khush and Brar 1991; Jairin et al. 2007a, 2007b; Jena and Kim 2010; Myint et al. 2012; Liu et al. 2015; Guo et al. 2018; Shi et al. 2021). The allelic polymorphism of *BPH9* confers diverse levels of resistance to multiple BPH biotypes (Zhao et al. 2016). However, 38 genes/loci could not be determined because only 1 biotype or population of BPH or new laboratory-selected colonies were used during the period of BPH resistance identification. As new biotypes of BPH are continuously increasing (Thanysiriwat et al. 2009; Listihani et al. 2022), more efforts are needed to identify the virulence shift in wild populations and to keep updating new biotype nomenclature over time. In rice breeding programs, 34 genes/loci have been introduced into rice cultivars to develop new varieties or breeding lines. A total of 7 genes/loci have been introgressed into rice cultivars and released to the public, while the other 27 genes/loci have just been introduced into advanced breeding lines (Supplemental Table 5).

Pyramided *R* genes have been discovered with additive resistance effects. For example, *BPH14* and *BPH15* have shown more durable resistance to BPH, while *qBPH3* and *qBPH4* display more resistance than *qBPH3* and *qBPH4* alone (Huang et al. 2001; Hu et al. 2015). *BPH1*-introgressed cultivars have lost control of BPH in Southeast Asia, where biotype 2 is widely distributed, but IR64 has shown durable and broad-spectrum resistance to different biotypes over two decades due to the major genes *BPH1* and *BPH37* and associated minor QTLs (Alam and Cohen 1998; Mackill and Khush 2018; Yang et al. 2019). However, gene combinations do not always lead to positive effects compared to a single *R* gene (Jena et al. 2017). Gene stacking has become the most effective way to develop a new variety harboring durable and broad-spectrum resistance in practical modern rice breeding programs (Mundt 2014; Muduli et al. 2021). To improve stable and durable resistance to BPH in rice and develop promising genetic sources for emerging BPH biotypes in advance, 2–4 *BPH* genes are stacked to develop new breeding materials (Supplemental Table 5) (Rahman et al. 2009; Zhu et al. 2013; Jena et al. 2017; Li et al. 2019b).

As a new method of improving disease resistance, the CRISPR/Cas9-mediated gene knockout of the cytochrome P450 gene *CYP71A1* (*S*-related factors) improves rice insect resistance, not only to BPH but also to WBPH and the striped stem borer (*Chilo suppressalis*) (Lu et al. 2018).

## Multiple disease or stress resistance in rice

Multiple disease resistance (MDR) refers to host plant resistance to a minimum of two diseases, excluding non-host resistance (Wiesner-Hanks and Nelson 2016). Although it was regarded as a valued trait for plant pathologists and breeders for over a century, genetic evidence and molecular mechanisms were not well studied until Wiesner-Hanks and Nelson’s review. MDR is considered a peculiar form of broad-spectrum resistance and is particularly difficult to overcome due to multiple unlinked genes/loci, clusters of tightly linked genes, and individual genes with pleiotropic effects (Wiesner-Hanks and Nelson 2016). MDR has been described as potentially important in barley and wheat (Pooja et al. 2014; Pal et al. 2022), while increasing genetic evidence in other plants, including rice, maize, coffee, and cotton, has been reported (Ali et al. 2013; de Almeida et al. 2021; Jamaloddin et al. 2021; Huo et al. 2023). In regard to sessile plants and ongoing climate change, the idea of MDR might extend to the resistance or tolerance to two or more multiple stresses, both abiotic and biotic, such as drought, heat, chilling, flood, fungi, bacteria, viruses, parasitic plants, and insects. This concept has been accepted and cited in the second-generation ‘new plant type’ (NPT-2) initiative and the Green Super Rice (GSR) project (Bin Rahman and Zhang 2023).

In rice, 40 defense regulator genes coding various proteins in different biological activities, as summarized by Liu et al. (2021b), have been found to be involved in MDR, 29 of which contribute to resistance to both blast and BLB (Liu et al. 2021b). Even a series of developmental genes, protein networks, signaling pathways, pleiotropic genes, and a set of response factors in plants can confer multiple stress resistance (Chun et al. 2012; Jacob et al. 2017; Cohen and Leach 2019; Zhang et al. 2021b; Husaini 2022). However, there have been few practical applications of these genes in crop breeding. Most plant *R* genes are race-specific, conforming to the typical gene-for-gene relationship, but some exhibit non-race-specific resistance to at least two pathogens, insects, or stresses. For example, wheat *Lr34* and *Lr67* confer resistance to both rust disease and powdery mildew (Krattinger et al. 2009; Moore et al. 2015), even *Lr34* confers resistance to rust disease and northern corn leaf blight in maize and barley and blast disease in rice (Risk et al. 2013; Krattinger et al. 2016; Sucher et al. 2017). As stated previously, *BPH3*, *BPH6*, *bph25*, *BPH30*, and *qBPH6(t)* confer resistance to both BPH and WBPH in rice (Khush and Brar 1991; Jairin et al. 2007a, 2007b; Jena and Kim 2010; Myint et al. 2012; Liu et al. 2015; Guo et al. 2018; Shi et al. 2021). Rice *OslecRK* can induce an immune response to fungal and bacterial diseases, as well as BPH (Cheng et al. 2013b).

Thus far, an effective strategy for producing MDR or multiple stress resistance has been to combine multiple *R* genes into a single crop genotype, which can simultaneously increase the durability of resistance (Wiesner-Hanks and Nelson 2016; Mundt 2018). Due to MAS, it is possible to pyramid multiple stress resistance genes using molecular markers rather than phenotyping in different stress environments. Marker-assisted forward breeding and marker-assisted backcross breeding have been used to develop rice cultivars with multiple *R* genes or QTLs (Dixit et al. 2020; Ramalingam et al. 2020). For example, *Pi9* for blast, *Xa21* for BLB, *Gm8* for gall midge (a serious rice pest), and three major QTLs, *qDTY1*.*1*, *qDTY2*.*2*, and *qDTY4*.*1*, were introduced into an elite Indian rice variety, ‘Naveen,’ in six years (Janaki Ramayya et al. 2021). The new approaches could not reduce the time of the breeding project, as the selected crop plants had to be tested and evaluated in the field (Ben-Ari and Lavi 2012). In addition, the development of multiple *R* gene pyramids usually takes a great deal of time because a series of backcrosses and subsequent MAS are needed to avoid the loss of gene effectiveness and durability before the cultivars introgressed with multiple *R* genes are released to the public (Stam and McDonald 2018). As Supplemental Table 6 shows, among 133 super rice cultivars in China, only 40 cultivars show resistance to one stress, 2 cultivars exhibit resistance to two stresses, and none show resistance to blast, BLB, and BPH. Similarly, among 154 varieties released by the Central Varietal Release Committee in India (Supplemental Table 7), a total of 64 cultivars show resistance to one stress, 20 cultivars exhibit resistance to two stresses, and 4 varieties show resistance to three stresses (Table 2). Rice breeders now know of many resistance genes; however, most breeding programs emphasize monogenic resistance. It seems that pyramiding of *R* genes to produce multiple stress or disease resistance has attracted less attention and effort than stress or disease resistance to a single stress.

Another strategy for stacking resistance is multigene transformation. At first, scientists combined a single gene transformation with hybrid polymerization (Datta et al. 2002). However, this method is time-consuming and requires hybridization and screening work to confirm pyramided transgenic plants. Another method, multi-vector co-transformation, has been used to transfer multiple genes in separate vectors into the same recipient plant at the same time, but this method is uncertain and unstable. Multigene single-vector transformation utilizes multiple genes on one plasmid, which can then be transferred to the same recipient plant at one time. This method has received much more attention due to its easy manipulation and time and labor efficiency (Shehryar et al. 2020; Li et al. 2021a). The only hurdle is placing multiple genes on a single T-DNA, which is being resolved by the development of a high-efficiency transgene stacking system, such as the TransGene Stacking II (TGSII) system (Zhu et al. 2017) and GAANTRY (Gene Assembly in *Agrobacterium* by Nucleic acid Transfer using Recombinase Technology) system (Collier et al. 2018). A new method, named HACKing (Highly Efficient and Accessible System by CracKing Genes into the Genome) system based on CRISPR/Cas9 genome editing, has been developed to assemble multigene pathways in fungi. This system provides high and stable expression of multiple genes and more choice for different conditions based on the researcher’s demands (Yue et al. 2023). Therefore, it can be quickly modified for use in other organisms, especially plants. Recently, a new approach to obtaining MDR in rice has been reported, in which multiple *S* genes are modified by CRISPR/Cas9-mediated gene knockout. A rice mutant inhibiting *pi21*, *bsr-d1*, and *xa5* shows enhanced resistance to blast disease and BLB (Tao et al. 2021). Both transgenic stacking genes and modifying genes are logically time- and labor-saving methods compared to multiple gene pyramiding. However, it will still be a long time before they are widely used, as transgenic and cisgenic gene cassettes are strictly required for government approval before field deployment (Stam and McDonald 2018). For example, since the first transgenic tobacco plants were developed in 1983 (Herrera-Estrella et al. 1983), only 463 transgenic events from the 32 main crops have been approved according to the genetically modified crops database of the International Service for the Acquisition of Agri-Biotech Applications (ISAAA, http://www.isaaa.org/gmapprovaldatabase/default.asp). Among them, only 6 events from rice and only 2 events for 2 genes conferring resistance to 2 Lepidopteran insects have been reported.

## Conclusion and perspectives

Rice breeding is an ancient activity that dates back to the dawn of agriculture (Wallace et al. 2018; Xu and Sun 2021). The multi-origin domestications of *Oryza sativa* from wild to cultivated rice began more than 10,000 years ago, when the morphological traits, physiological characteristics, and ecological adaptability were greatly improved by local and independent famers’ conscious and unconscious selection in the fields (Sweeney and McCouch 2007; Xue et al. 2021; Zhang et al. 2021a). After Mendelian genetics was found and introduced into breeding programs between the late nineteenth and early twentieth century, plant breeding entered scientific conventional breeding (Wallace et al. 2018). With the fast development of molecular genetics and biotechnology, especially MAS applied extensively in plant breeding programs, marker-assisted breeding has developed a great number of crop cultivars with important agronomic traits to alleviate global hunger in the past half century. For example, the semidwarf-1 (*sd1*) gene, a ‘Green Revolution’ gene in rice, has been widely deployed in modern rice varieties, increasing rice yields to a new peak in the world (Cheng et al. 2022). Plant breeding has now developed into an applied, multidisciplinary science utilizing the maximum benefits from genomic, phenomic, and enviromic data (Gepts and Hancock 2006; Crossa et al. 2021).

Thus far, pyramiding multiple *R* genes/QTLs is the most effective method for increasing the durability of resistance and broadening the pathogen spectrum in rice breeding. Although this approach has many empirical applications, scientists and breeders are not certain about the final effects of multiple *R* gene combinations. The main reason is that multiple genes do not mean one gene function added to another gene function, as there is a potentially complex relationship that could result in an unexpected phenotype. This complex relationship cannot be understood clearly only from the *R* gene/QTLs studied from a single perspective or several related perspectives, such as gene function, signal pathway, or regulatory mechanism, but also from systemic and multi-omics studies, such as genomics, transcriptomics, metabolomics, and phenomics. Such great systemic and multi-omics work cannot be accomplished in only a wet laboratory but a dry laboratory.

Machine learning (ML) refers to computational algorithms that turn datasets from large-scale observations into usable models (Edgar and Manz 2017). It has shown rapid development and has been deployed in multi-dimensional phenotyping, plant–pathogen interactions, and plant breeding, including QTL mapping, genetic architecture assessment, and genomic prediction (Sperschneider 2020; Wang et al. 2020; van Dijk et al. 2021). Multiple stress resistance genes have been identified in rice using ML (Shaik and Ramakrishna 2013). Artificial intelligence (AI) refers to systems imitated by a computer to display intelligent behavior similar to humans but with some degree of autonomy (Sheikh et al. 2023). It has acted as a revolution in our society, impacting industry, business, agriculture, and science. It can turn traditional experience-based selection into precision molecular design in breeding (Liu et al. 2021a) and reshape modern crop breeding into smart breeding (Khan et al. 2022; Xu et al. 2022). In addition, AI integrated with speed breeding can accelerate crop breeding due to resistance to multiple stresses from unfavorable environmental conditions and infectious living organisms (Watson et al. 2018; Rai 2022). Therefore, ML and AI can help us not only discover the genetic basis of durable and broad-spectrum resistance but also quickly design and develop new rice cultivars with durable and broad-spectrum resistance to major diseases and insects for specific geographic locations in the near future.

## Supplementary Information

Below is the link to the electronic supplementary material.

Supplementary file1 (PDF 192 KB)

Supplementary file2 (PDF 263 KB)

Supplementary file3 (PDF 213 KB)

Supplementary file4 (PDF 257 KB)

Supplementary file5 (PDF 213 KB)

Supplementary file6 (PDF 291 KB)

Supplementary file7 (PDF 147 KB)
