# Supplementary material for: Current status of molecular rice breeding for durable and broad-spectrum resistance to major diseases and insect pests
Source: Theor Appl Genet. 2024 Sep 10;137(10):219. doi: 10.1007/s00122-024-04729-3 (PMC11387466; doi:10.1007/s00122-024-04729-3)
Supplement: Supplementary file 1 — Supplementary file1 (PDF 192 KB) [file 122_2024_4729_MOESM1_ESM.pdf]

Supplemental Table1. Details on rice genes/Loci with broad-spectrum and durable resistance to blast disease

	Donor	Chr	Resistance	References
Pi1	LAC23	11	durable resistance to diverse isoates in China	(Hua et al., 2012)
Pi2/Piz-5	5173	6	resistance to 36 isolates from 10 countries	(Liu et al., 2002)
Pi3/Pi5	C104PKT	5	broad-spectrum resistance in the Philippines, Indonesia and Korea	(Jeon et al., 2003)
Pi7(t)	RIL29 (Moroberekan)	11	broad-spectrum resistance in the Philippines	(Wang et al., 1994; Telebanco-Yanoria et al., 2010)
Pi9	75-1-127 (O. minuta)	6	durable, resistance to 43 isolates from 13 countries, over 100 isolates atat the International Rice Research Institute (IRRI)	(Liu et al., 2002; Qu et al., 2006)
pi21	Owarihatamochi (OW)	4	durable, broad-spectrum resistance	(Fukuoka and Okuno, 2001; Fukuoka et al., 2009)
Pi25	Gumei2	6	durable resistance in China, and resistance to multiple isolates	(Wu et al., 2005; Chen et al., 2011)
Pi33	IRGC101508 (O. rufipogon)	8	broad-spectrum resistance	(Berruyer et al., 2003; Ballini et al., 2007)
Pi35	Hokkai 188	1	durable, broad-spectrum resistance	(Fukuoka et al., 2014)
Pi39	Q15	12	broad spectrum resistance	(Hua et al., 2015)
Pi40	O. australiensis (Acc. 100882)	6	broad-spectrum durable resistance in Korea	(Jena et al., 2007; Jeung et al., 2007)
Pi41	93-11	12	broad spectrum resistance in three Provinces of China	(Yang et al., 2009)
Pi46	H4	11	broad spectrum resistance in Guangdong, China	(Xiao et al., 2011)
Pi47	Xiangzi 3150	11	broad spectrum resistance in Guangdong, China	(Huang et al., 2011)
Pi48	Xiangzi 3150	12	broad spectrum resistance in Hunan, China	(Huang et al., 2011)
Pi49	Mowanggu	11	broad spectrum resistance in 6 Provinces of China, durable resistance	(Sun et al., 2013)
Pi50	Er-Ba-Zhan (EBZ)	6	resistance to some indica-sourced isolates	(Zhu et al., 2012; Su et al., 2015)
Pi54/Pi-kh	Tetep	11	broad spectrum resistance in India	(Sharma et al., 2005)
Pi56(t)	Sanhuangzhan No 2 (SHZ-2)	9	broad-spectrum resistance	(Liu et al., 2013)
Pi57(t)	IL-E1454 (O. longistaminata)	12	broad-spectrum resistance	(Dong et al., 2017)
Pi63	Kahei	4	broad-spectrum resistance	(Xu et al., 2014a)
Pi64	Yangmaogu (YMG)	1	resistance to some indica-sourced isolates	(Ma et al., 2015)
Pb1	Modan	11	durable, broad-spectrum resistance	(Hayashi et al., 2010)
Pia	Zenith	11	resistance to two isolates from the Philippines and isolates from 2 provinces of China	(Telebanco-Yanoria et al., 2010b; Zeng et al., 2011)
Pib	BL1	2	resistance to six isolates from the Philippines and most isolates from Japan	(Wang et al., 1999; Telebanco-Yanoria et al., 2010b)
Pid3	Digu	6	broad spectrum resistance in China	(Shang et al., 2009; Xu et al., 2014b)
Pi-d(t)	Digu	2	broad spectrum resistance in China	(Chen et al., 2004)
Pii	Hitomebore	9	resistance to several isolates from Korea	(Suh et al., 2009; Takagi et al., 2013)
Pik	Kusabue, Kanto51	11	resistance to many isolates from China and the Philipies, stable resistance	(Telebanco-Yanoria et al., 2010b; Zhai et al., 2011)
Pik-m	Tsuyuake	11	broad spectrum resistance	(Ashikawa et al., 2008)
Pik-p	K60	11	broad spectrum resistance	(Yuan et al., 2011)
Pik-s	Shin 2	11	broad spectrum resistance	(Wang et al., 2009)
Pish	Shin 2	1	durable resistance to many isolates from the Philipines	(Koide et al., 2010)
Pit	Tjahaja, K59	1	broad spectrum resistance	(Hayashi and Yoshida, 2009)
Pita	YT14	12	resistance to some isolates from the Philipies	(Bryan et al., 2000; Telebanco-Yanoria et al., 2010)
Pita2/Ptr	Pi No4	12	broader resistance spectrum than Pita	(Meng et al., 2020)
Piz	Zenith	6	broad spectrum resistance	(Suh et al., 2009)

Piz-t	Toride 1	6	broad spectrum resistance	(Zhou et al., 2006)
Pizh	Zhonghua11 (ZH11)	6	durable and broad spectrum resistance	(Xie et al., 2019)
Pigm	Gumei 4	6	durable and broad-spectrum resistance	(Deng et al., 2017)
qBl1/QTL1	Jao Hom Nin (JHN)	1	broad spectrum resistance	(Sreewongchai et al., 2010; Wongsaprom et al., 2010)
qBl11QTL11	Jao Hom Nin (JHN)	11	broad spectrum resistance	(Sreewongchai et al., 2010; Wongsaprom et al., 2010)
QTL2	IR64	2	broad spectrum resistance	(Sreewongchai et al., 2010)
QTL12	IR64	12	broad spectrum resistance	(Sreewongchai et al., 2010)

References

Ashikawa, I., Hayashi, N., Yamane, H., Kanamori, H., Wu, J., Matsumoto, T., Ono, K., and Yano, M. (2008). Two Adjacent Nucleotide-Binding Site–Leucine-Rich Repeat Class Genes Are Required to Confer Pikm-Specific Rice Blast Resistance. Genetics **180,** 2267-2276.

Ballini, E., Berruyer, R., Morel, J.-B., Lebrun, M.-H., Nottéghem, J.-L., and Tharreau, D. (2007). Modern elite rice varieties of the 'Green Revolution' have retained a large introgression from wild rice around the Pi33 rice blast resistance locus. New Phytologist **175,** 340-350.

Berruyer, R., Adreit, H., Milazzo, J., Gaillard, S., Berger, A., Dioh, W., Lebrun, M.H., and Tharreau, D. (2003). Identification and fine mapping of Pi33, the rice resistance gene corresponding to the Magnaporthe grisea avirulence gene ACE1. Theoretical and Applied Genetics **107,** 1139-1147.

Bryan, G.T., Wu, K.-S., Farrall, L., Jia, Y., Hershey, H.P., McAdams, S.A., Faulk, K.N., Donaldson, G.K., Tarchini, R., and Valent, B. (2000). A Single Amino Acid Difference Distinguishes Resistant and Susceptible Alleles of the Rice Blast Resistance Gene Pi-ta. The Plant Cell **12,** 2033-2045.

Chen, J., Shi, Y., Liu, W., Chai, R., Fu, Y., Zhuang, J., and Wu, J. (2011). A Pid3 allele from rice cultivar Gumei2 confers resistance to Magnaporthe oryzae. Journal of Genetics and Genomics **38,** 209-216.

Chen, X.W., Li, S.G., Xu, J.C., Zhai, W.X., Ling, Z.Z., Ma, B.T., Wang, Y.P., Wang, W.M., Cao, G., Ma, Y.Q., Shang, J.J., Zhao, X.F., Zhou, K.D., and Zhu, L.H. (2004). Identification of Two Blast Resistance Genes in a Rice Variety, Digu. Journal of Phytopathology **152,** 77-85.

Deng, Y., Zhai, K., Xie, Z., Yang, D., Zhu, X., Liu, J., Wang, X., Qin, P., Yang, Y., Zhang, G., Li, Q., Zhang, J., Wu, S., Milazzo, J., Mao, B., Wang, E., Xie, H., Tharreau, D., and He, Z. (2017). Epigenetic regulation of antagonistic receptors confers rice blast resistance with yield balance. Science **355,** 962-965.

Dong, L., Liu, S., Xu, P., Deng, W., Li, X., Tharreau, D., Li, J., Zhou, J., Wang, Q., Tao, D., and Yang, Q. (2017). Fine mapping of Pi57(t) conferring broad spectrum resistance against Magnaporthe oryzae in introgression line IL-E1454 derived from Oryza longistaminata. PLOS ONE **12,** e0186201.

Fukuoka, S., and Okuno, K. (2001). QTL analysis and mapping of pi21, a recessive gene for field resistance to rice blast in Japanese upland rice. Theoretical and Applied Genetics **103,** 185-190.

Fukuoka, S., Saka, N., Koga, H., Ono, K., Shimizu, T., Ebana, K., Hayashi, N., Takahashi, A., Hirochika, H., Okuno, K., and Yano, M. (2009). Loss of Function of a Proline-Containing Protein Confers Durable Disease Resistance in Rice. Science **325,** 998-1001.

Fukuoka, S., Yamamoto, S.-i., Mizobuchi, R., Yamanouchi, U., Ono, K., Kitazawa, N., Yasuda, N., Fujita, Y., Nguyen, T., Koizumi, S., Sugimoto, K., Matsumoto, T., and Yano, M. (2014). Multiple functional polymorphisms in a single disease resistance gene in rice enhance durable resistance to blast. Scientific Reports **4**.

Hayashi, K., and Yoshida, H. (2009). Refunctionalization of the ancient rice blast disease resistance gene Pit by the recruitment of a retrotransposon as a promoter. The Plant Journal **57,** 413-425.

Hayashi, N., Inoue, H., Kato, T., Funao, T., Shirota, M., Shimizu, T., Kanamori, H., Yamane, H., Hayano-Saito, Y., Matsumoto, T., Yano, M., and Takatsuji, H. (2010). Durable panicle blast-resistance gene Pb1 encodes an atypical CC-NBS-LRR protein and was generated by acquiring a promoter through local genome duplication. The Plant Journal **64,** 498-510.

Hua, L.-X., Liang, L.-Q., He, X.-Y., Wang, L., Zhang, W.-S., Liu, W., Liu, X.-Q., and Lin, F. (2015). Development of a marker specific for the rice blast resistance gene Pi39 in the Chinese cultivar Q15 and its use in genetic improvement. Biotechnology & Biotechnological Equipment **29,** 448-456.

Hua, L., Wu, J., Chen, C., Wu, W., He, X., Lin, F., Wang, L., Ashikawa, I., Matsumoto, T., Wang, L., and Pan, Q. (2012). The isolation of *Pi1*, an allele at the *Pik* locus which confers broad spectrum resistance to rice blast. Theoretical and Applied Genetics **125,** 1047-1055.

Huang, H., Huang, L., Feng, G., Wang, S., Wang, Y., Liu, J., Jiang, N., Yan, W., Xu, L., Sun, P., Li, Z., Pan, S., Liu, X., Xiao, Y., Liu, E., Dai, L., and Wang, G.-L. (2011). Molecular Mapping of the New Blast Resistance Genes Pi47 and Pi48 in the Durably Resistant Local Rice Cultivar Xiangzi 3150. Phytopathology® **101,** 620-626.

Jena, K., Suh, J., Jeung, J., Cho, Y., Roh, J., Han, S., Kim, Y., and Brar, D. (2007). Molecular breeding for durable blast disease resistance in rice.

Jeon, J.S., Chen, D., Yi, G.H., Wang, G.L., and Ronald, P.C. (2003). Genetic and physical mapping of *Pi5(t)*, a locus associated with broad-spectrum resistance to rice blast. Molecular Genetics and Genomics **269,** 280-289.

Jeung, J.-U., Kim, B., Cho, Y.C., Han, S.S., Moon, H.P., Lee, Y.T., and Jena, K.K. (2007). A novel gene, Pi40(t), linked to the DNA markers derived from NBS-LRR motifs confers broad spectrum of blast resistance in rice. Theoretical and Applied Genetics **115,** 1163-1177.

Koide, Y., Kawasaki, A., Telebanco-Yanoria, M.J., Hairmansis, A., Nguyet, N.T.M., Bigirimana, J., Fujita, D., Kobayashi, N., and Fukuta, Y. (2010). Development of pyramided lines with two resistance genes, Pish and Pib, for blast disease (Magnaporthe oryzae B. Couch) in rice (Oryza sativa L.). Plant Breeding **129,** 670-675.

Liu, G., Lu, G., Zeng, L., and Wang, G.L. (2002). Two broad-spectrum blast resistance genes, *Pi9(t)* and *Pi2(t)*, are physically linked on rice chromosome 6. Molecular Genetics and Genomics **267,** 472-480.

Liu, Y., Liu, B., Zhu, X., Yang, J., Bordeos, A., Wang, G., Leach, J.E., and Leung, H. (2013). Fine-mapping and molecular marker development for Pi56(t), a NBS-LRR gene conferring broad-spectrum resistance to Magnaporthe oryzae in rice. Theoretical and Applied Genetics **126,** 985-998.

Ma, J., Lei, C., Xu, X., Hao, K., Wang, J., Cheng, Z., Ma, X., Ma, J., Zhou, K., Zhang, X., Guo, X., Wu, F., Lin, Q., Wang, C., Zhai, H., Wang, H., and Wan, J. (2015). Pi64, Encoding a Novel CC-NBS-LRR Protein, Confers Resistance to Leaf and Neck Blast in Rice. Molecular Plant-Microbe Interactions® **28,** 558-568.

Meng, X., Xiao, G., Telebanco-Yanoria, M.J., Siazon, P.M., Padilla, J., Opulencia, R., Bigirimana, J., Habarugira, G., Wu, J., Li, M., Wang, B., Lu, G.-d., and Zhou, B. (2020). The broad-spectrum rice blast resistance (R) gene Pita2 encodes a novel R protein unique from Pita. Rice **13,** 19.

Qu, S., Liu, G., Zhou, B., Bellizzi, M., Zeng, L., Dai, L., Han, B., and Wang, G.-L. (2006). The Broad-Spectrum Blast Resistance Gene Pi9 Encodes a Nucleotide-Binding Site–Leucine-Rich Repeat Protein and Is a Member of a Multigene Family in Rice. Genetics **172,** 1901-1914.

Shang, J., Tao, Y., Chen, X., Zou, Y., Lei, C., Wang, J., Li, X., Zhao, X., Zhang, M., Lu, Z., Xu, J., Cheng, Z., Wan, J., and Zhu, L. (2009). Identification of a New Rice Blast Resistance Gene, Pid3, by Genomewide Comparison of Paired Nucleotide-Binding Site–Leucine-Rich Repeat Genes and Their Pseudogene Alleles Between the Two Sequenced Rice Genomes. Genetics **182,** 1303-1311.

Sharma, T.R., Madhav, M.S., Singh, B.K., Shanker, P., Jana, T.K., Dalal, V., Pandit, A., Singh, A., Gaikwad, K., Upreti, H.C., and Singh, N.K. (2005). High-resolution mapping, cloning and molecular characterization of the Pi-khgene of rice, which confers resistance to Magnaporthe grisea. Molecular Genetics and Genomics **274,** 569-578.

Sreewongchai, T., Toojinda, T., Thanintorn, N., Kosawang, C., Vanavichit, A., Tharreau, D., and Sirithunya, P. (2010). Development of elite indica rice lines with wide spectrum of resistance to Thai blast isolates by pyramiding multiple resistance QTLs. Plant Breeding **129,** 176-180.

Su, J., Wang, W., Han, J., Chen, S., Wang, C., Zeng, L., Feng, A., Yang, J., Zhou, B., and Zhu, X. (2015). Functional divergence of duplicated genes results in a novel blast resistance gene Pi50 at the Pi2/9 locus. Theoretical and Applied Genetics **128,** 2213-2225.

Suh, J.P., Roh, J.H., Cho, Y.C., Han, S.S., Kim, Y.G., and Jena, K.K. (2009). The Pi40 Gene for Durable Resistance to Rice Blast and Molecular Analysis of Pi40-Advanced Backcross Breeding Lines. Phytopathology **99,** 243-250.

Sun, P., Liu, J., Wang, Y., Jiang, N., Wang, S., Dai, Y., Gao, J., Li, Z., Pan, S., Wang, D., Li, W., Liu, X., Xiao, Y., Liu, E., Wang, G.-L., and Dai, L. (2013). Molecular mapping of the blast resistance gene Pi49 in the durably resistant rice cultivar Mowanggu. Euphytica **192,** 45-54.

Takagi, H., Uemura, A., Yaegashi, H., Tamiru, M., Abe, A., Mitsuoka, C., Utsushi, H., Natsume, S., Kanzaki, H., Matsumura, H., Saitoh, H., Yoshida, K., Cano, L.M., Kamoun, S., and Terauchi, R. (2013). MutMap-Gap: whole-genome resequencing of mutant F2 progeny bulk combined with de novo assembly of gap regions identifies the rice blast resistance gene Pii. New Phytologist **200,** 276-283.

Telebanco-Yanoria, M.J., Koide, Y., Fukuta, Y., Imbe, T., Kato, H., Tsunematsu, H., and Kobayashi, N. (2010). Development of near-isogenic lines of Japonica-type rice variety Lijiangxintuanheigu as differentials for blast resistance. Breeding Science **60,** 629-638.

Wang, G.L., Mackill, D.J., Bonman, J.M., McCouch, S.R., Champoux, M.C., and Nelson, R.J. (1994). RFLP mapping of genes conferring complete and partial resistance to blast in a durably resistant rice cultivar. Genetics **136,** 1421-1434.

Wang, L., Xu, X., Lin, F., and Pan, Q. (2009). Characterization of Rice Blast Resistance Genes in the Pik Cluster and Fine Mapping of the Pik-p Locus. Phytopathology **99,** 900-905.

Wang, Z.-X., Yano, M., Yamanouchi, U., Iwamoto, M., Monna, L., Hayasaka, H., Katayose, Y., and Sasaki, T. (1999). The Pib gene for rice blast resistance belongs to the nucleotide binding and leucine-rich repeat class of plant disease resistance genes. The Plant Journal **19,** 55-64.

Wongsaprom, C., Sirithunya, P., Vanavichit, A., Pantuwan, G., Jongdee, B., Sidhiwong, N., Lanceras-Siangliw, J., and Toojinda, T. (2010). Two introgressed quantitative trait loci confer a broad-spectrum resistance to blast disease in the genetic background of the cultivar RD6 a Thai glutinous jasmine rice. Field Crops Research **119,** 245-251.

Wu, J.L., Fan, Y.Y., Li, D.B., Zheng, K.L., Leung, H., and Zhuang, J.Y. (2005). Genetic control of rice blast resistance in the durably resistant cultivar Gumei 2 against multiple isolates. Theoretical and Applied Genetics **111,** 50-56.

Xiao, W., Yang, Q., Wang, H., Guo, T., Liu, Y., Zhu, X., and Chen, Z. (2011). Identification and fine mapping of a resistance gene to Magnaporthe oryzae in a space-induced rice mutant. Molecular Breeding **28,** 303-312.

Xie, Z., Yan, B., Shou, J., Tang, J., Wang, X., Zhai, K., Liu, J., Li, Q., Luo, M., Deng, Y., and He, Z. (2019). A nucleotide-binding site-leucine-rich repeat receptor pair confers broad-spectrum disease resistance through physical association in rice. Philosophical Transactions of the Royal Society B: Biological Sciences **374,** 20180308.

Xu, X., Hayashi, N., Wang, C.-T., Fukuoka, S., Kawasaki, S., Takatsuji, H., and Jiang, C.-J. (2014a). Rice blast resistance gene Pikahei-1(t), a member of a resistance gene cluster on chromosome 4, encodes a nucleotide-binding site and leucine-rich repeat protein. Molecular Breeding **34,** 691-700.

Xu, X., Lv, Q., Shang, J., Pang, Z., Zhou, Z., Wang, J., Jiang, G., Tao, Y., Xu, Q., Li, X., Zhao, X., Li, S., Xu, J., and Zhu, L. (2014b). Excavation of Pid3 Orthologs with Differential Resistance Spectra to Magnaporthe oryzae in Rice Resource. PLOS ONE **9,** e93275.

Yang, Q., Lin, F., Wang, L., and Pan, Q. (2009). Identification and mapping of Pi41, a major gene conferring resistance to rice blast in the Oryza sativa subsp. indica reference cultivar, 93-11. Theoretical and Applied Genetics **118,** 1027-1034.

Yuan, B., Zhai, C., Wang, W., Zeng, X., Xu, X., Hu, H., Lin, F., Wang, L., and Pan, Q. (2011). The Pik-p resistance to Magnaporthe oryzae in rice is mediated by a pair of closely linked CC-NBS-LRR genes. Theoretical and Applied Genetics **122,** 1017-1028.

Zeng, X., Yang, X., Zhao, Z., Lin, F., Wang, L., and Pan, Q. (2011). Characterization and fine mapping of the rice blast resistance gene Pia. Science China Life Sciences **54,** 372-378.

Zhai, C., Lin, F., Dong, Z., He, X., Yuan, B., Zeng, X., Wang, L., and Pan, Q. (2011). The isolation and characterization of Pik, a rice blast resistance gene which emerged after rice domestication. New Phytologist **189,** 321-334.

Zhou, B., Qu, S., Liu, G., Dolan, M., Sakai, H., Lu, G., Bellizzi, M., and Wang, G.-L. (2006). The Eight Amino-Acid Differences Within Three Leucine-Rich Repeats Between Pi2 and Piz-t Resistance Proteins Determine the Resistance Specificity to Magnaporthe grisea. Molecular Plant-Microbe Interactions® **19,** 1216-1228.

Zhu, X., Chen, S., Yang, J., Zhou, S., Zeng, L., Han, J., Su, J., Wang, L., and Pan, Q. (2012). The identification of Pi50(t), a new member of the rice blast resistance Pi2/Pi9 multigene family. Theoretical and Applied Genetics **124,** 1295-1304.
