# Supplementary material for: Current status of molecular rice breeding for durable and broad-spectrum resistance to major diseases and insect pests
Source: Theor Appl Genet. 2024 Sep 10;137(10):219. doi: 10.1007/s00122-024-04729-3 (PMC11387466; doi:10.1007/s00122-024-04729-3)
Supplement: Supplementary file 2 — Supplementary file2 (PDF 263 KB) [file 122_2024_4729_MOESM2_ESM.pdf]

Supplemental Table2. Genes with broad-spectrum and durable resistance to blast in rice breeding by MAS

	Improved variety/line	References
Pi1	US-2, Pusa Basmati 1 (PB1), II-32B, IR64, GD-8S, Samba Mahsuri (BPT 5204)	(Jin et al., 2007; Khanna et al., 2015; Guan et al., 2019; Sudarsanam et al., 2019; Fukuta et al., 2022b; Fukuta et al., 2022a)
Pi2/Piz-5	Yangdao 6, LijiangxintLijiangxintuanheigu (LTH), PB1, Hui 316, Pusa Basmati 1121 (PB1121), Pusa Basmati 6, Boyarin, IR64, BPT5204, C815S, RPHR-1005, IR50, PRR78	(Narayanan et al., 2002; Telebanco-Yanoria et al., 2010; Singh et al., 2012b; Jiang et al., 2015; Khanna et al., 2015; Ellur et al., 2016; Kumar et al., 2016; Usatov et al., 2016; Wu et al., 2016; Sagar KrishnaMurthy et al., 2017; Tian et al., 2019; Fukuta et al., 2022a)
Pi3/Pi5	US-2, LTH, PB1, IR64, Samba Mahsuri (BPT5204)	(Telebanco-Yanoria et al., 2010; Khanna et al., 2015; Sagar KrishnaMurthy et al., 2017; Fukuta et al., 2022b; Fukuta et al., 2022a)
Pi7(t)	US-2, LTH, IR64	(Telebanco-Yanoria et al., 2010; Fukuta et al., 2022b; Fukuta et al., 2022a)
Pi9	US-2, LTH,Hui316, R894, Yangdao 6, PB1, II-32B, 07GY31, C815S, R6547, R599, R288, Shuhui527, Minghui86, Minhui3301, X5, 03S, Luhui17, E32, Q211S, IR64, Huhan 1B	(Telebanco-Yanoria et al., 2010; Yin et al., 2011; Wen and Gao, 2012; Cao et al., 2015; Khanna et al., 2015; Wang et al., 2015; Zhang et al., 2015; Wu et al., 2016; Xing et al., 2016; Chen et al., 2017; Xiao et al., 2017; Zou et al., 2017; Huang et al., 2018; Guan et al., 2019; Tian et al., 2019; Chen et al., 2020; Zhang et al., 2021; Fukuta et al., 2022b; Fukuta et al., 2022a)
pi21	Mineasahi, Koshihikari, IR63307-4B-13-2, IRBB4/5/13/21, Kinandang Patong (KP), Aichiasahi (AA), Kongyu-131	(Fukuoka et al., 2009; Saka et al., 2010; Fukuoka et al., 2015; Feng et al., 2019; Angeles-Shim et al., 2020)
Pi25	C815S, Zhenda A, Xiangwanxian No.13	(Cao et al., 2015; Dong et al., 2017; Liu et al., 2017; Chen et al., 2020)
Pi35	Koshihikari	(Yasuda et al., 2015)
Pi39	Yuexiangzhan (YXZ), Yueyinsimiao (YYSM)	(Hua et al., 2015)
Pi40	Yangdao 6, Osmancik-97, Halilbey	(Beşer et al., 2016; Wu et al., 2016)
Pi49	C815S	(Cao et al., 2015; Chen et al., 2020)
Pi54/Pi-kh	US-2, LTH, PB1, samba mahsuri (BPT 5204), K343, II-32B, IR 58025A, PRR78, Improved Pusa Basmati 1, Pusa Basmati 1121 (PB1121), Pusa Basmati 6, 07GY31, IR64	(Telebanco-Yanoria et al., 2010; Yin et al., 2011; Wen and Gao, 2012; Cao et al., 2015; Khanna et al., 2015; Wang et al., 2015; Zhang et al., 2015; Wu et al., 2016; Xing et al., 2016; Chen et al., 2017; Xiao et al., 2017; Zou et al., 2017; Huang et al., 2018; Guan et al., 2019; Tian et al., 2019; Chen et al., 2020; Liu et al., 2021; Zhang et al., 2021; Fukuta et al., 2022b; Fukuta et al., 2022a)
Pb1	Tsukinohikari, Asanohikari, Aoinokaze, Yumeminori, Akanezora, Maturibare, Goropikari, Koigokoro, Asahinoyume, Daichinokaze, AichinokaoriSBL, KoshihikariAichiSBL, Sainokagayaki, Tsuyaotome, Yumematsuri, Kinuhanamochi, Yumeginga, Koharumochi, Harumoni, Sainokizuna, Kiraho, Ginganoshizuku, MineasahiSBL, Yawakoimochi, Nijinokirameki, Ilmi, Saeilmi	(Lee et al., 2015; Fujii et al., 2023)
Pia	US-2, LTH	(Telebanco-Yanoria et al., 2010; Fukuta et al., 2022)
Pib	LTH, Pib	(Telebanco-Yanoria et al., 2010; Khanna et al., 2015)
Pid3	Fanyuan B	(Hong-Guang et al., 2016)
Pii	US-2, IR64	(Fukuta et al., 2022b; Fukuta et al., 2022a)
Pik	US-2, LTH, IR64	(Telebanco-Yanoria et al., 2010; Fukuta et al., 2022b; Fukuta et al., 2022a)
Pik-m	US-2	(Fukuta et al., 2022)
Pik-p	US-2, IR64	(Fukuta et al., 2022b; Fukuta et al., 2022a)
Pik-s	US-2, LTH	(Telebanco-Yanoria et al., 2010; Fukuta et al., 2022)
Pish	IR64	(Fukuta et al., 2022a)
Pit	Chucheong	(Kwon et al., 2008)
Pita	US-2, LTH, PB1	(Telebanco-Yanoria et al., 2010; Khanna et al., 2015; Fukuta et al., 2022)
Pita2/Ptr	US-2, LTH	(Telebanco-Yanoria et al., 2010; Fukuta et al., 2022)
Piz	US-2, Yangdao 6, IR64	(Wu et al., 2016; Fukuta et al., 2022b; Fukuta et al., 2022a)
Piz-t	Yangdao 6, Chucheong, US-2, 07GY31	(Kwon et al., 2008; Wu et al., 2016; Xiao et al., 2017; Fukuta et al., 2022b)
Pizh	Kongyu131	(Xie et al., 2019)

Pigm	KT27S, Chunhui350, Wuyungeng 32/WYG32, Huageng 8/HG8), Yangdao 6, Minghui86, C815S	(Cao et al., 2015; Yang et al., 2015; Wu et al., 2016; Dai et al., 2018; Chen et al., 2020; Yang et al., 2020; Feng et al., 2022)
Pi1, Pi2/Piz-5	II-32B, Rongfeng B, Peiai64S, GD-7S, Guihong 1, PRR78, Intan	(Liu et al., 2008; Dong et al., 2010; Liu et al., 2012; Gouda et al., 2013; Hedge and Prashanthi, 2016; Sun et al., 2018; Guan et al., 2019)
Pi2, Pi5	PB1	(Khanna et al., 2015)
Pi1, Pi9	II-32B	(Guan et al., 2019)
Pi2, Pi9	R179	(Luo et al., 2017)
Pi5, Pi9	PB1	(Khanna et al., 2015)
Pi1, Pi33	Boyarin	(Usatov et al., 2016)
Pi47, Pi48	C815S	(Cao et al., 2015; Chen et al., 2020)
Pi9, Pi49	Chuang 5S	(Zhang et al., 2018)
Pi54, Pi1	PB1, Swarna-Sub1,	(Khanna et al., 2015; Patroti et al., 2019; Jamaloddin et al., 2020)
Pi2, Pi54	Improved Samba Mahsuri (ISM), Pusa Basmati 1121 (PB1121), Pusa Basmati 6,	(Ellur et al., 2016; Madhavi et al., 2016; Patroti et al., 2019)
Pi9, Pi54	07GY31	(Xiao et al., 2017)
Pi1,Pi-kh	II-32B	(Guan et al., 2019)
Pi2, Pi-kh	II-32B	(Guan et al., 2019)
Pi9, Pi-kh	II-32B	(Guan et al., 2019)
Pi5, Pib	PB1	(Khanna et al., 2015)
Pi9, Pib	PB1	(Khanna et al., 2015)
Pi2, Pib	PB1	(Khanna et al., 2015)
pi21, Pi35	Koshihikari	(Yasuda et al., 2015)
Pb1, Pi39	Mineasahi	(Suzuki et al., 2017)
Pib, Pi-kh	MR219	(Tanweer et al., 2015)
Pib, Pik	Junam	(Reinke et al., 2018)
Pib, Pish	CO 39	(Koide et al., 2010)
Pik, Pish	Kho Khor 6 (RD6)	(Ngernmuen et al., 2020)
Pi46, Pita	Hang-Hui-179 (HH179)	(Xiao et al., 2016)
Pi1, Pita	PB1	(Khanna et al., 2015)
Pi54, Pita	PB1	(Khanna et al., 2015)
Pita, Pita2	Chucheong	(Kwon et al., 2008)
Piz-t, Pi54	07GY31	(Xiao et al., 2017)
Pi9, Piz-t	07GY31	(Xiao et al., 2017)
qBl1/QTL1, qBl11QTL11	RD6	(Wongsaprom et al., 2010)
Pi1, Pi2, Pi33	Kuboyar, ADT43, Jin 23B	(Chen et al., 2008; Divya et al., 2014; Usatov et al., 2016)
Pi1, Pi2, Pi54	Swarna-Sub1	(Patroti et al., 2019)
Pi1, Pi9, Pi-kh	II-32B	(Guan et al., 2019)
Pi5, Pi9, Pi54	Huhan 1S	(Liu et al., 2021)
Pia, Pik, Pik-p	Chucheong	(Kwon et al., 2008)
Pi1, Pi54, Pita	PB1, Mushk Budji	(Khan et al., 2018; Khanna et al., 2015)
Piz, Piz-t, Pi9	Chucheong	(Kwon et al., 2008)
Pi-d(t), Pib, Pita2	G46B	(Chen et al., 2004)
Piz, Pi2, Pi9	MR219	(Miah et al., 2017)
Pi1, Piz-5, Pita	CO39	(Hittalmani et al., 2000)
Pia, Pik, Pik-p, Pik-m	Chucheong	(Kwon et al., 2008)
Pia, Pib, Pik, Pik-p	Chucheong	(Kwon et al., 2008)
Pia, Pii, Pik, Pik-p, Pik-m	Chucheong	(Kwon et al., 2008)
Pia, Pik, Pik-p, Pita2	Chucheong	(Kwon et al., 2008)
qBl1/QTL1, qBl11/QTL11,QTL 2, QTL12	IR64, Jao Hom Nin (JHN)	(Sreewongchai et al., 2010)
Pia, Pib, Pik, Pik-p, Pita2	Chucheong	(Kwon et al., 2008)

References

Angeles-Shim, R.B., Reyes, V.P., del Valle, M.M., Lapis, R.S., Shim, J., Sunohara, H., Jena, K.K., Ashikari, M., and Doi, K. (2020). Marker-Assisted Introgression of Quantitative Resistance Gene pi21 Confers Broad Spectrum Resistance to Rice Blast. Rice Science **27,** 113-123.

Beşer, N., Valle, M.M.d., Kim, S.M., Vinarao, R., Sürek, H., and Jena, K.K. (2016). Marker-assisted Introgression of a Broad-spectrum Resistance Gene, Pi40 Improved Blast Resistance of Two Elite Rice (Oryza sativa L.) Cultivars of Turkey. Molecular plant breeding **7**.

Cao, Z., Zeng, G., Hao, M., Sheng, H., Ye, N., and Xiao, Y. (2015). Improving Blast Resistance of Dual-purpose Genic Sterile Line C815S by Using Molecular Marker-assisted Selection. Molecular Plant Breeding **13,** 1193-1200.

Chen, H., Chen, Z., Ni, S., Zuo, S., Pan, X., and Zhu, X. (2008). Pyramiding three genes with resistance to blast by markerassisted selection to improve rice blast resistance of Jin 23B. Chinese Journal of Rice Science **22,** 23-27.

Chen, H., Li, Y., Liu, X., Liu, J., Yang, F., Xing, X., Liao, H., Huang, J., and Liu, J. (2017). Improving blast resistance of Indica rice restorer R599 and its hybrid by MAS technology. Hybrid Rice **32,** 61-65.

Chen, Q., Zeng, G., Hao, M., Jiang, H., and Xiao, Y. (2020). Improvement of rice blast and brown planthopper resistance of PTGMS line C815S in two-line hybrid rice through marker-assisted selection. Molecular Breeding **40,** 21.

Chen, X.W., Li, S.G., Ma, Y.Q., Li, H.Y., Zhou, K.D., and Zhu, L.H. (2004). Marker-assisted selection and pyramiding for three blast resistance genes, Pi-d(t)1, Pi-b, Pi-ta2, in rice. Sheng Wu Gong Cheng Xue Bao **20**, 708-714.

Dai, X., He, C., Zhou, L., Liang, M., Fu, X., Qin, P., Yang, Y., and Chen, L. (2018). Identification of a specific molecular marker for the rice blast-resistant gene Pigm and molecular breeding of thermo-sensitive genic male sterile leaf-color marker lines. Molecular Breeding **38,** 72.

Divya, B., Robin, S., Rabindran, R., Senthil, S., Raveendran, M., and Joel, A.J. (2014). Marker assisted backcross breeding approach to improve blast resistance in Indian rice (Oryza sativa) variety ADT43. Euphytica **200,** 61-77.

Dong, R., Wang, H., Dong, L., Zhou, P., Tu, S., You, Q., Liang, F., and Huang, T. (2017). Improving the rice blast resistance for a CMS line of rice Zhenda A and its hybrids using molecular marker-assistant selection. Journal of Plant Genetic Resources **18,** 573-586.

Dong, W., Li, X., Yan, B., Wu, C., Gao, G., Bao, L., Li, Y., Zhu, X., and He, Y. (2010). Improving the blast resistance of Peiai64S through markerassisted selection. Molecular Plant Breeding **8,** 853-860.

Ellur, R.K., Khanna, A., Yadav, A., Pathania, S., Rajashekara, H., Singh, V.K., Gopala Krishnan, S., Bhowmick, P.K., Nagarajan, M., Vinod, K.K., Prakash, G., Mondal, K.K., Singh, N.K., Vinod Prabhu, K., and Singh, A.K. (2016). Improvement of Basmati rice varieties for resistance to blast and bacterial blight diseases using marker assisted backcross breeding. Plant Science **242,** 330-341.

Feng, X., Lin, K., Zhang, W., Nan, J., Zhang, X., Wang, C., Wang, R., Jiang, G., Yuan, Q., and Lin, S. (2019). Improving the blast resistance of the elite rice variety Kongyu-131 by updating the pi21 locus. BMC Plant Biology **19,** 249.

Feng, Z., Li, M., Xu, Z., Gao, P., Wu, Y., Wu, K., Zhao, J., Wang, X., Wang, J., Li, M., Hu, K., Chen, H., Deng, Y., Li, A., Chen, Z., and Zuo, S. (2022). Development of Rice Variety With Durable and Broad-Spectrum Resistance to Blast Disease Through Marker-Assisted Introduction of Pigm Gene. Frontiers in Plant Science **13**.

Fujii, K., Suzuki, T., Nakamura, M., Yoshida, T., Uchikawa, Y., Suwazono, H., Hayashi, N., Kanda, Y., and Inoue, H. (2023). Investigating the Mechanisms Underlying the Durability and Sustainable Use of Pb1 Gene-Mediated High Field Resistance to Rice Panicle Blast. Agronomy **13,** 1751.

Fukuoka, S., Saka, N., Mizukami, Y., Koga, H., Yamanouchi, U., Yoshioka, Y., Hayashi, N., Ebana, K., Mizobuchi, R., and Yano, M. (2015). Gene pyramiding enhances durable blast disease resistance in rice. Scientific Reports **5,** 7773.

Fukuoka, S., Saka, N., Koga, H., Ono, K., Shimizu, T., Ebana, K., Hayashi, N., Takahashi, A., Hirochika, H., Okuno, K., and Yano, M. (2009). Loss of Function of a Proline-Containing Protein Confers Durable Disease Resistance in Rice. Science **325,** 998-1001.

Fukuta, Y., Telebanco-Yanoria, M.J., Koide, Y., Saito, H., Kobayashi, N., Obara, M., and Yanagihara, S. (2022a). Near-isogenic lines for resistance to blast disease, in the genetic background of the Indica Group rice (Oryza sativa L.) cultivar IR64. Field Crops Research **282,** 108506.

Fukuta, Y., Koide, Y., Kobayashi, N., Kato, H., Saito, H., Telebanco-Yanoria, M.J., Ebron, L.A., Mercado-Escueta, D., Tsunematsu, H., Ando, I., Fujita, D., Obara, M., Tomita, A., Hayashi, N., and Imbe, T. (2022b). Lines for blast resistance genes with genetic background of Indica Group rice as international differential variety set. Plant Breeding **141,** 609-620.

Gouda, P.K., Saikumar, S., Varma, C.M.K., Nagesh, K., Thippeswamy, S., Shenoy, V., Ramesha, M.S., and Shashidhar, H.E. (2013). Marker-assisted breeding of Pi-1 and Piz-5 genes imparting resistance to rice blast in PRR78, restorer line of Pusa RH-10 Basmati rice hybrid. Plant Breeding **132,** 61-69.

Guan, H., Hou, X., Jiang, Y., Srivastava, V., Mao, D., Pan, R., Chen, M., Zhou, Y., Wang, Z., and Chen, Z. (2019). Feature of blast resistant near-isogenic lines using an elite maintainer line II-32B by marker-assisted selection. Journal of Plant Pathology **101,** 491-501.

Hari, Y., Srinivasarao, K., Viraktamath, B.C., Hari Prasad, A.S., Laha, G.S., I. Ahmed, M., Natarajkumar, P., Sujatha, K., Srinivas Prasad, M., Pandey, M., Ramesha, M.S., Neeraja, C.N., Balachandran, S.M., S. Rani, N., Kemparaju, B., Madhan Mohan, K., Sama, V.S.A.K., Shaik, H., Balachiranjeevi, C., Pranathi, K., Ashok Reddy, G., Madhav, M.S., and Sundaram, R.M. (2013). Marker-assisted introgression of bacterial blight and blast resistance into IR 58025B, an elite maintainer line of rice. Plant Breeding **132,** 586-594.

Hedge, S.S., and Prashanthi, S.K. (2016). Identification of polymorphic markers and introgression of Pi1 and Pi2 genes for blast resistance in rice. Journal of Farm Sciences **29,** 327-331 ref.317.

Hittalmani, S., Parco, A., Mew, T.V., Zeigler, R.S., and Huang, N. (2000). Fine mapping and DNA marker-assisted pyramiding of the three major genes for blast resistance in rice. Theoretical and Applied Genetics **100,** 1121-1128.

Hong-Guang, X., Jia-Huang, J., Yan-Mei, Z., Yong-Sheng, Z., Fang-Xi, W., Xi, L., Qiu-hua, C., Jian-Fu, Z., and Hua-An, X. (2016). Development of Hybrid Rice Variety FY7206 with Blast Resistance Gene Pid3 and Cold Tolerance Gene Ctb1. Rice Science **23,** 266-273.

Hua, L.-X., Liang, L.-Q., He, X.-Y., Wang, L., Zhang, W.-S., Liu, W., Liu, X.-Q., and Lin, F. (2015). Development of a marker specific for the rice blast resistance gene Pi39 in the Chinese cultivar Q15 and its use in genetic improvement. Biotechnology & Biotechnological Equipment **29,** 448-456.

Huang, Y., Yan, Z., Wang, H., Shen, G., and Zhang, C. (2018). Directed improvement of rice blast resistance of sterile line Q211S with molecular marker-assisted selection. Chinese Agricultural Science Bulletin **34,** 135-140.

Jamaloddin, M., Durga Rani, C.V., Swathi, G., Anuradha, C., Vanisri, S., Rajan, C.P.D., Krishnam Raju, S., Bhuvaneshwari, V., Jagadeeswar, R., Laha, G.S., Prasad, M.S., Satyanarayana, P.V., Cheralu, C., Rajani, G., Ramprasad, E., Sravanthi, P., Arun Prem Kumar, N., Aruna Kumari, K., Yamini, K.N., Mahesh, D., Sanjeev Rao, D., Sundaram, R.M., and Madhav, M.S. (2020). Marker Assisted Gene Pyramiding (MAGP) for bacterial blight and blast resistance into mega rice variety "Tellahamsa". PLOS ONE **15,** e0234088.

Jiang, J., Mou, T., Yu, H., and Zhou, F. (2015). Molecular breeding of thermo-sensitive genic male sterile (TGMS) lines of rice for blast resistance using Pi2 gene. Rice **8,** 11.

Jin, S., Liu, W., Zhu, X., Wang, F., Li, J., Liu, Z., Liao, Y., Zhu, M., Huang, H., and Liu, Y. (2007). Improving Blast Resistance of a Thermo-Sensitive Genic Male Sterile Rice Line GD-8S by Molecular Marker-Assisted Selection. Chinese Journal of Rice Science **21,** 599-604.

Jong-Hee, L., lt, sup, gt, lt, sup, gt, Ji-Yoon, L., lt, sup, gt, lt, sup, gt, Young-Nam, Y., lt, sup, gt, lt, sup, gt, Sang-Yeol, K., lt, sup, gt, lt, sup, gt, Yeon-Jae, H., lt, sup, gt, lt, sup, gt, Un-Sang, Y., lt, sup, gt, lt, sup, gt, Young-Bo, S., lt, sup, gt, lt, sup, gt, You-Chun, S., lt, sup, gt, lt, sup, gt, Dong-Soo, P., lt, sup, gt, lt, sup, gt, Min-Hee, N., lt, sup, gt, lt, sup, gt, and Jun-Hyeon, C., lt, sup, gt, lt, sup, and gt. (2015). Enhancement of Panicle Blast Resistance in Korean Rice Cultivar 'Saeilmi' by Marker Assisted Backcross Breeding. Plant Breed. Biotech. **3,** 1-10.

Khan, G.H., Shikari, A.B., Vaishnavi, R., Najeeb, S., Padder, B.A., Bhat, Z.A., Parray, G.A., Bhat, M.A., Kumar, R., and Singh, N.K. (2018). Marker-assisted introgression of three dominant blast resistance genes into an aromatic rice cultivar Mushk Budji. Scientific Reports **8,** 4091.

Khanna, A., Sharma, V., Ellur, R.K., Shikari, A.B., Gopala Krishnan, S., Singh, U.D., Prakash, G., Sharma, T.R., Rathour, R., Variar, M., Prashanthi, S.K., Nagarajan, M., Vinod, K.K., Bhowmick, P.K., Singh, N.K., Prabhu, K.V., Singh, B.D., and Singh, A.K. (2015). Development and evaluation of near-isogenic lines for major blast resistance gene(s) in Basmati rice. Theoretical and Applied Genetics **128,** 1243-1259.

Kiran, U., Sharma, M., Punya, Salgotra, R., Singh, B., and Singh, A. (2020). Introgression of *Pi54* gene through marker assisted backcross breeding for development of blast resistant genetic stocks in rice. Journal of Pharmacognosy and Phytochemistry **9,** 1034-1040.

Koide, Y., Kawasaki, A., Telebanco-Yanoria, M.J., Hairmansis, A., Nguyet, N.T.M., Bigirimana, J., Fujita, D., Kobayashi, N., and Fukuta, Y. (2010). Development of pyramided lines with two resistance genes, Pish and Pib, for blast disease (Magnaporthe oryzae B. Couch) in rice (Oryza sativa L.). Plant Breeding **129,** 670-675.

Kumar, V.A., Balachiranjeevi, C.H., Naik, S.B., Rambabu, R., Rekha, G., Harika, G., Hajira, S.K., Pranathi, K., Vijay, S., Anila, M., Mahadevaswamy, H.K., Kousik, M., Yugander, A., Aruna, J., Hari Prasad, A.S., Madhav, M.S., Laha, G.S., Balachandran, S.M., Prasad, M.S., Babu, V.R., and Sundaram, R.M. (2016). Marker-assisted improvement of the elite restorer line of rice, RPHR-1005 for resistance against bacterial blight and blast diseases. Journal of genetics **95,** 895-903.

Kwon, S.-W., Cho, Y.-C., Kim, Y.-G., Suh, J.-P., Jeung, J.-U., Roh, J.-H., Lee, S.-K., Jeon, J.-S., Yang, S.-J., and Lee, Y.-T. (2008). Development of near-isogenic Japonica rice lines with enhanced resistance to Magnaporthe grisea. Mol. Cells **25,** 407-416.

Lee, J.-H., Lee, J.-Y., Yoon, Y.-N., Kim, S.-Y., Hur, Y.-J., Yeo, U.-S., Sohn, Y.-B., Song, Y.-C., Park, D.-S., Nam, M.-H., and Cho, J.-H. (2015). Enhancement of Panicle Blast Resistance in Korean Rice Cultivar 'Saeilmi' by Marker Assisted Backcross Breeding. Plant Breed. Biotech. **3,** 1-10.

Liu, W., Li, X., Li, Y., Pan, X., Sheng, X., and Duan, Y. (2017). Improvement of rice blast resistance of Xiangwanxian No.13 with high quality by molecular marker-assisted selection. Molecular Plant Breeding **15,** 3063-3069.

Liu, W., Wang, F., Liu, Z., Zhu, X., Li, J., Huang, H., Liao, Y., Zhu, M., Fu, C., and Chen, J. (2012). Improvement of rice blast resistance in CMS line Rongfeng A by pyramiding Pi-1 and Pi-2 with molecular marker techniques. Molecular Plant Breeding **10,** 575-582.

Liu, W., Wang, F., Jin, S., Zhu, X., Li, J., Liu, Z., Liao, Y., Zhu, M., Huang, H., Fu, F., and Liu, Y. (2008). Improvement of rice blast resistance in TGMS line by pyramiding of Pi-1 and Pi-2 through molecular marker-assisted selection. Acta Agronomica Sinica **34,** 1128-1136.

Liu, Y., Zhang, F., Luo, X., Kong, D., Zhang, A., Wang, F., Pan, Z., Wang, J., Bi, J., Luo, L., Liu, G., and Yu, X. (2021). Molecular Breeding of a Novel PTGMS Line of WDR for Broad-Spectrum Resistance to Blast Using Pi9, Pi5, and Pi54 Genes. Rice **14,** 96.

Luo, W., Huang, M., Guo, T., Xiao, W., Wang, J., Yang, G., Liu, Y., Wang, H., Chen, Z., and Zhuang, C. (2017). Marker-assisted selection for rice blast resistance genes Pi2 and Pi9 through high-resolution melting of a gene-targeted amplicon. Plant Breeding **136,** 67-73.

Madhavi, K., Rambabu, R., Kumar, V., Sudarsanam, V., Aruna, J., Ramesh, S., Sundaram, R., Laha, G.s., Sheshu madhav, M., Babu, V., and Prasad, M. (2016). Marker assisted introgression of blast (Pi-2 and Pi-54) genes in to the genetic background of elite, bacterial blight resistant indica rice variety, Improved Samba Mahsuri. Euphytica **212**.

Miah, G., Rafii, M.Y., Ismail, M.R., Puteh, A.B., Rahim, H.A., and Latif, M.A. (2017). Marker-assisted introgression of broad-spectrum blast resistance genes into the cultivated MR219 rice variety. Journal of the Science of Food and Agriculture **97,** 2810-2818.

Narayanan, N.N., Baisakh, N., Vera Cruz, C.M., Gnanamanickam, S.S., Datta, K., and Datta, S.K. (2002). Molecular Breeding for the Development of Blast and Bacterial Blight Resistance in Rice cv. IR50. Crop Science **42,** 2072-2079.

Ngernmuen, A., Suktrakul, W., Katengam, S., and Jantasuriyarat, C. (2020). Transcriptome Comparison of Defense Responses in the Rice Variety 'Jao Hom Nin' Regarding Two Blast Resistant Genes, Pish and Pik. Plants **9,** 694.

Patroti, P., Vishalakshi, B., Umakanth, B., Suresh, J., Senguttuvel, P., and Madhav, M.S. (2019). Marker-assisted pyramiding of major blast resistance genes in Swarna-Sub1, an elite rice variety (Oryza sativa L.). Euphytica **215,** 179.

Reinke, R., Kim, S.-M., and Kim, B.-K. (2018). Developing japonica rice introgression lines with multiple resistance genes for brown planthopper, bacterial blight, rice blast, and rice stripe virus using molecular breeding. Molecular Genetics and Genomics **293,** 1565-1575.

Sagar KrishnaMurthy, P., Deshmukh, D.B., Yashvanth Kumar, K.J., Patil, S., Jakkeral, S., Nemappa, G.H., Singh, U.D., Variar, M., Rathour, R., Subbaiyan, G., Singh, A.K., and Sharma, T.R. (2017). Introgression of Pi2 and Pi5 Genes for Blast (Magnaporthe oryzae) Resistance in Rice and Field Evaluation of Introgression Lines for Resistance and Yield Traits. Journal of Phytopathology **165,** 397-405.

Saka, N., Fukuoka, S., Terashima, T., Kudo, S., Shirota, M., Ando, I., Sugiura, K., Sato, H., Maeda, H., Endo, I., Kato, H., and Inoue, M. (2010). Breeding of a new rice variety "Chubu 125" with high field resistance for blast and excellent eating quality, pp. 171-183.

Singh, A., Singh, V.K., Singh, S.P., Pandian, R.T.P., Ellur, R.K., Singh, D., Bhowmick, P.K., Gopala Krishnan, S., Nagarajan, M., Vinod, K.K., Singh, U.D., Prabhu, K.V., Sharma, T.R., Mohapatra, T., and Singh, A.K. (2012a). Molecular breeding for the development of multiple disease resistance in Basmati rice. AoB PLANTS **2012**.

Singh, V.K., Singh, A., Singh, S.P., Ellur, R.K., Choudhary, V., Sarkel, S., Singh, D., Krishnan, S.G., Nagarajan, M., Vinod, K.K., Singh, U.D., Rathore, R., Prashanthi, S.K., Agrawal, P.K., Bhatt, J.C., Mohapatra, T., Prabhu, K.V., and Singh, A.K. (2012b). Incorporation of blast resistance into "PRR78", an elite Basmati rice restorer line, through marker assisted backcross breeding. Field Crops Research **128,** 8-16.

Sreewongchai, T., Toojinda, T., Thanintorn, N., Kosawang, C., Vanavichit, A., Tharreau, D., and Sirithunya, P. (2010). Development of elite indica rice lines with wide spectrum of resistance to Thai blast isolates by pyramiding multiple resistance QTLs. Plant Breeding **129,** 176-180.

Sudarsanam, V., Rambabu, R., Phaneendra, B., Madhavi, K., Sabbu, S., Vellaichamy, P., Sundaram, R., Sheshu madhav, M., Venkata SubbaRao, L., and Prasad, M. (2018). Introgression of durable blast resistance gene Pi-54 into indica rice cv. samba mahsuri, through Marker Assisted Backcross Breeding **9,** 705-715.

Sudarsanam, V., Prasad, M., Rambabu, R., Madhavi, K., Phaneendra, B., Kumar, V., Sundaram, R., Satya, A., Sheshu madhav, M., and Vellaichamy, P. (2019). Marker-Assisted Introgression of Pi-1 Gene Conferring Resistance to Rice Blast Pathogen Pyricularia oryzae in the Background of Samba Mahsuri. International Journal of Current Microbiology and Applied Sciences **8,** 2133-2146.

Sun, F., Tang, M., He, C., and Lu, H.-c. (2018). Improvement of rice blast resistance in red rice by pyramiding of Pi1 and Pi2 through molecular marker-assisted selection. Hubei Agricultural Sciences **57,** 23-27.

Suzuki, T., Nakamura, M., Saka, N., Ikeda, A., Terashima, T., Mizukami, Y., Nonoyama, T., Yoshida, T., Shirota, M., and Kato, T. (2017). Breeding of a new rice variety" Chubu138", a near-isogenic line of" Mineasahi", with blast and rice stripe disease resistance. Research Bulletin of the Aichi Agricultural Research Center**,** 93-102.

Tanweer, F.A., Rafii, M.Y., Sijam, K., Rahim, H.A., Ahmed, F., Ashkani, S., and Latif, M.A. (2015). Introgression of Blast Resistance Genes (Putative Pi-b and Pi-kh) into Elite Rice Cultivar MR219 through Marker-Assisted Selection. Frontiers in Plant Science **6**.

Telebanco-Yanoria, M.J., Koide, Y., Fukuta, Y., Imbe, T., Kato, H., Tsunematsu, H., and Kobayashi, N. (2010). Development of near-isogenic lines of Japonica-type rice variety Lijiangxintuanheigu as differentials for blast resistance. Breeding Science **60,** 629-638.

Tian, D., Guo, X., Zhang, Z., Wang, M., and Wang, F. (2019). Improving blast resistance of the rice restorer line, Hui 316, by introducing Pi9 or Pi2 with marker-assisted selection. Biotechnology & Biotechnological Equipment **33,** 1195-1203.

Usatov, A., Kostylev, P., Azarin, K., Markin, N., Makarenko, M., Khachumov, V., and Bibov, M. (2016). Introgression of the rice blast resistance genes Pi1, Pi2 and Pi33 into Russian rice varieties by marker-assisted selection. Indian Journal of Genetics and Plant Breeding (The) **76,** 18.

Wang, H., Chen, J., Zhou, G., Shen, G., Jiang, J., Zhang, C., Fang, Y., and Huang, Y. (2015). Improving the rice blast resistance of sterile line 03S by molecular marker-assisted selection. Journal of Yangzhou University **36,** 74-78.

Wen, S., and Gao, B. (2012). Introgressing blast resistant gene Pi-9 (t) into elite rice restorer Luhui17 by marker-assisted selection. Molecular Plant Breeding **10**.

Wongsaprom, C., Sirithunya, P., Vanavichit, A., Pantuwan, G., Jongdee, B., Sidhiwong, N., Lanceras-Siangliw, J., and Toojinda, T. (2010). Two introgressed quantitative trait loci confer a broad-spectrum resistance to blast disease in the genetic background of the cultivar RD6 a Thai glutinous jasmine rice. Field Crops Research **119,** 245-251.

Wu, Y., Yu, L., Pan, C., Dai, Z., Li, Y., Xiao, N., Zhang, X., Ji, H., Huang, N., Zhao, B., Zhou, C., Liu, G., Liu, X., Pan, X., Liang, C., and Li, A. (2016). Development of near-isogenic lines with different alleles of Piz locus and analysis of their breeding effect under Yangdao 6 background. Molecular Breeding **36,** 12.

Xiao, N., Wu, Y., Pan, C., Yu, L., Chen, Y., Liu, G., Li, Y., Zhang, X., Wang, Z., Dai, Z., Liang, C., and Li, A. (2017). Improving of Rice Blast Resistances in Japonica by Pyramiding Major R Genes. Frontiers in Plant Science **7**.

Xiao, W.-m., Luo, L.-x., Wang, H., Guo, T., Liu, Y.-z., Zhou, J.-y., Zhu, X.-y., Yang, Q.-y., and Chen, Z.-q. (2016). Pyramiding of Pi46 and Pita to improve blast resistance and to evaluate the resistance effect of the two R genes. Journal of Integrative Agriculture **15,** 2290-2298.

Xie, Z., Yan, B., Shou, J., Tang, J., Wang, X., Zhai, K., Liu, J., Li, Q., Luo, M., Deng, Y., and He, Z. (2019). A nucleotide-binding site-leucine-rich repeat receptor pair confers broad-spectrum disease resistance through physical association in rice. Philosophical Transactions of the Royal Society B: Biological Sciences **374,** 20180308.

Xing, X., Liu, X., Chen, H., Yang, F., Li, Y., Liao, H., You, L., Liu, J., Dai, L., and Wang, G. (2016). Improving blast resistance of rice restorer R288 by molecular marker-assisted selection of Pi9 Gene. Crop Research **30,** 487-491.

Yang, D., Li, S., Lu, L., Fang, J., Wang, W., Cui, H., and Tang, D. (2020). Identification and application of the Pigm-1 gene in rice disease resistance breeding. Plant Biology **22,** 1022-1029.

Yang, P., Zou, G., Chen, C., Huang, Y., Lan, B., Xiong, Y., and Yin, J. (2015). Improvement of rice blast resistance of Chunhui350 by using molecular-marker assisted selection. Molecular Plant Breeding **13,** 741-747.

Yasuda, N., Mitsunaga, T., Hayashi, K., Koizumi, S., and Fujita, Y. (2015). Effects of Pyramiding Quantitative Resistance Genes pi21, Pi34, and Pi35 on Rice Leaf Blast Disease. Plant Disease **99,** 904-909.

Yin, D., Xia, M., Li, J., Wan, B., Zha, Z., Du, X., and Qi, H. (2011). Development of STS marker linked to rice blast resistance gene Pi9 in marker-assisted selection breeding. Chinese Journal of Rice Science **25,** 25-30.

Zhang, H., Zhou, P., Tu, S., Zheng, J., Zhang, J., and Xie, H. (2015). Developing new restorer lines with blast-resistance gene Pi9 for hybrid rice by marker assistance selection (MAS). Molecular Plant Breeding **13,** 1918-1922.

Zhang, J., Hao, M., Zeng, G., Cao, Z., Jiang, H., Huang, X., and Xiao, Y. (2018). Polymerization of Pi9 and Pi49 loci by marker assisted selection to improve blast resistance of dual-purpose genic sterile rice Chuang 5S. Molecular Plant Breeding **16,** 7372-7379.

Zhang, X., Sun, J., Wang, Q., Wang, H., Li, J., Zhang, D., and Zhu, S. (2021). Breeding and Application of Blast-resistant Restorer Line Qianhui 101 in Rice. China Rice **27,** 108-110.

Zou, J., Li, Y., Liu, X., Liu, J., Chen, H., Yang, F., Huang, J., and Liao, H. (2017). Improving blast reisistance of rice restorer 'E32' and its hybrid through molecular marker-assisted selection. Crop Research **31,** 11-14.
