# Supplementary material for: Current status of molecular rice breeding for durable and broad-spectrum resistance to major diseases and insect pests
Source: Theor Appl Genet. 2024 Sep 10;137(10):219. doi: 10.1007/s00122-024-04729-3 (PMC11387466; doi:10.1007/s00122-024-04729-3)
Supplement: Supplementary file 3 — Supplementary file3 (PDF 213 KB) [file 122_2024_4729_MOESM3_ESM.pdf]

Supplemental Table3. Details on rice resistance genes to bacterial leaf blight disease

	Donor	Chr	Resistance	References
Xa1	IRBB1	4	resistance to Japanese race 1	(Yoshimura et al., 1998)
Xa2	IRBB2	4	resistance to Japanese race 2, Korean strains KXO85,JW89011, and Australian strains Aust-2013,Aust-R3	(He et al., 2006; Ji et al., 2020)
Xa3/Xa26	IRBB3, Minghui63	11	resistance to Philippine races 1,2,3,4,5, Japanese races 1,2,3, and Chinese strains HB84-17,Zhe173,LN85-57,JS49-6; durable resistance	(Kaku and Ogawa, 2000; Kaku and Ogawa, 2001; Sun et al., 2004; Xiang et al., 2006; Deng et al., 2018)
Xa4	IRBB4	11	resistance to Philippine races 1, 4, 5; durable resistance	(Wang et al., 2001; Hu et al., 2017)
xa5	IRBB5	5	resistance to Philippine races 1,2,3,5 and Indian races PXO 1,13,17	(Li et al., 2001; Iyer and McCouch, 2004)
Xa6/Xa3	Zenith, Malagkit Sungsong	11	resistance to Philippine races 1,2,3,4,5 and Japanese races	(Sidhu and Khush, 1978; Kaku and Ogawa, 2000)
Xa7	DV85	6	broad-spectrum and extremely durable resistance	(Vera Cruz et al., 2000; Porter et al., 2003; Chen et al., 2021)
xa8	PI 231129	7	resistance to Punjab races 1,2,3,4,6 and Philippine races 1,2,4,6	(Vikal et al., 2014)
xa9	Khao Lay Nhay, Sateng	11	resistance to Philippine races 1,3,4,5,6	(Singh et al., 1983; Hisatoshi, 1993; Yogesh and Dharminder, 2017)
Xa10	Cas 209	11	resistance to Philippine races	(Gu et al., 2008)
Xa11	IR8, IRBB11	3	resistance to Japanese races 1b,2,3a,5	(Goto et al., 2009)
Xa12	Kogyoku, Java 14	4	resistance to Indonesian group V	(OGAWA et al., 1978; Yamada, 1984; Bao et al., 2006)
xa13	BJ1, IRBB13	8	resistance to Philippine race 6 and Indian race PXO 8	(Li et al., 2001; Chu et al., 2006)
Xa14	Taichung Native 1 (TN1)	4	resistance to Philippine race 5	(Taura et al., 1987; Bao et al., 2010)
xa15	M41	-	resistance to Japanese races 1, 2, 3, 4	(Nakai et al., 1988)
Xa16	Tetep	-	resistance to Japanese race 7	(Noda and Ohuchi, 1989)
Xa17	Asominori	-	resistance to Japanese race 2	(Ogawa et al., 1989)
Xa18	IR24, Miyang23, Toyonishiki	-	resistance to Myanmar strains BM8417, BM8429	(Yamamoto and Ogawa, 1990)
xa19	XM5 (mutant of IR24)	7	resistance to Philippine races 1,2,3,4,5,6 and Japanese races 1,2,3,4,5	(Taura et al., 1991; Taura and Ichitani, 2023)
xa20	XM6 (mutant of IR24)	3	resistance to Philippine races 1,2,3,4,5,6 and Japanese races 1,2,3,4,5	(Taura et al., 1992; Msami et al., 2021)
Xa21	*O. longistaminata*	11	resistance to Philippine races 1,2,3,4,5,6 and Indian races	(Khush et al., 1990; Ronald et al., 1992; Song et al., 1995)
Xa22(t)	Zhachanglong	11	broad-spectrum resistance to Philippine, Japanese and Chinese isolates	(Lin et al., 1996; Wang et al., 2003)
Xa23	*O. rufipogon*	11	extremely broad resistance spectrum to rances in the Philippines, Japan, China, Korea, Bangladesh and Africa	(Zhang et al., 2001; Wang et al., 2014; Wang et al., 2015)
xa24	DV86	2	resistance to Philippine races 4,6,10, and Chinese stains Zhe173, JL691,KS-1-21	(Wu et al., 2008)
xa25	Minghui 63	12	resistance to Philippine race 9	(Chen et al., 2002; Liu et al., 2011)
Xa25	HX-3 (mutant of Minghui 63)	4	resistance to Philippine races 1,3,4, Japanese races 3,4, and Chinese strains BJ84-3, KW-2,4, KS-66, ZJ173, OS34, OS218, RB209, ZJ26, RBFJ21, GD-32, OS37,X-S-2, ZJ25, HB8417, GX-09, Hen114, ZJ28, JS158-2, OS35, OS171, OS225, #2	(Gao et al., 2002; Gao et al., 2005)
xa26(t)	Nep Bha Bong To	-	resistance to Philippine races 1,2,3,5	(Lee et al., 2003)

Xa27(t)	*O. minuta* Acc. 101141, IRBB27	6	resistance to Philippine races 2,3,4,5,6, Japanese races 1,4, Chinese strains C1, C2, C3, C5, C6, C7, GD1358, HB17, HB21, HLJ72, JS49-6, LN57, NX42, Australian strains Aust-2031, Aust-R3, Columbia strain CIAT1185, Indian isolates A3842, A3857, Indoneisan strain IXO56, Korean strain JW89011, Nepal strain NXO260, Thailand strains	(Gu et al., 2004)
xa28(t)	Lota Sail	-	resistance to Philippine races 2,5	(Lee et al., 2003)
Xa29(t)	B5 (*O. officinalis*)	1	resistance to Philippine race 1	(Tan et al., 2004)
Xa30(t)	Y238 (*O. rufipogon*)	11	resistance to Philippine races 2,3,5,6,7,8,9	(Wang et al., 2004; Jin et al., 2007)
Xa31(t)	Zhachanglong	4	resistance to Philippine race 5, Chinese strains OS105, HB17, Korean strains KXO85,JW89011, Japanese race 2, and Australian strains Aust-2013, Aust-R3	(Wang et al., 2009; Ji et al., 2020)
Xa32(t)	C4064 (*O. ustraliensis*)	11	resistance to Philippine races 1,4,5,6,7,8,9, and Korean strain KX085	(Zheng et al., 2009)
xa32(t)	Y76 (O. meyeriana)	12	resistance to Philippine race 6	(Ruan et al., 2008)
Xa33	*O. nivara* IRGC 105710	7	resistance to Indian isolates DX-022, DX-002, DX-018, DX-027, DX-049, DX-084, DX-066	(Kumar et al., 2012)
xa33(t)	Ba7	6	resistance to Thai isolates TB9602, TXO16, TXO55, TXO56, TXO111, TXO114, TXO121,	(Korinsak et al., 2009)
Xa34(t)	*O. rufipogon*	-	broad resistance spectrum to local isolates including Indian isolate DX-020	(Ram et al., 2010)
xa34(t)	BG1222	1	resistance to Chinese races 1,2,3,4,5,6,7; broad spectrum resistance to the local races	(Chen et al., 2011)
Xa35(t)	*O. minuta* Acc. 101133	11	resistance to Philippine races 1,5,9	(Guo et al., 2010)
Xa36(t)	C4059	11	broad resistance spectrum to local isolates including Philippine race 6, and Chinese race 5	(Miao et al., 2010)
Xa38	*O. nivara* (IRGC 81825)	4	broad resistance spectrum to seven Indian local pathotypes	(Vikal et al., 2007; Cheema et al., 2008)
Xa39	PSBRC66 (P66)	11	resistance to Philippine races 1,2,3b,3c,4,5,6,7,8,9a,9b,9c,9d,10, Chinese stains GD1358, KS6-6, Z173, OS198, JS49-6, GD-IV, GD-V	(Zhang et al., 2015)
Xa40(t)	IR65482-7-216-1-2	11	resistance to Korean races K1,K2,K3,K3a	(Kim et al., 2015)
xa41(t)	*O. barthii, O. glaberrima*	11	broad-spectrum resistance to Asian and African races	(Hutin et al., 2015)
xa42	XM14 (mutant of IR24)	3	resistance to Japanese races 1,2a,2b,3,4,5	(Busungu et al., 2016; Busungu et al., 2018)
xa42(t)	Baixiangzhan (BXZ)	6	resistance to Chinese races 1,2,3,4,5	(Zeng et al., 2009; Liang et al., 2017)
Xa43(t)	P8 (Colombia XXI; IRGC: 126955)	11	resistance to Korean isolates HB 1009,1013,1014,1015,2010,2024, 2038,3011,3034,3055,3079,4024, 4027,4030,4032,4040,4044,4052, 4074,4079,4084,4087,5004,6142	(Kim, 2018; Kim and Reinke, 2019)
xa44(t)	IR73571-3B-11-3-K3 (P6)	11	resistance to Korean isolates HB 1009,1013,1014,1015,2010,2024, 2038,3011,3034,4027,4030,4032, 4040,4052,4074,4079,4084,4087, 5004,6142	(Kim, 2018)
Xa45(t)	*O. nivara*, IRGC 102463	4	resstance to African strain AXO1947 and Korean strain KXO85	(Ji et al., 2020)
xa45(t)	*O. glaberrima* IRGC 102600B	8	resistance to Indian pathotypes 1,2,3,4,5,6,7,8,9,10	(Neelam et al., 2020)
Xa46	*O. rufipogen*	-	resistance to Korean strains J18	(He, 2021)
Xa46(t)	H120 (mutant of Lijiangxintuanheigu)	11	resistance to Chinese pathotypes I (GD9240), II (GD9269), III (GD9279), IV (GD9315), V (GD9352), and IX (GD9385)	(Chen et al., 2020)

Xa47	G252	11	resistance to Philippine race 6, Japanese race 2, Chinese races 5,9, and local strains Y8, Hzhj19, YM1, YM187, Yjdp-2, Yjws-2; durable resistance	(Xing et al., 2021; Lu et al., 2022)

References

Bao, S.-y., Tan, M.-p., and Lin, X.-h. (2006). Genetic mapping of the region including a bacterial blight resistance gene Xa12 in rice. Subtropical Plant Science **35,** 1.

Bao, S.-Y., Tan, M.-P., and Lin, X. (2010). Genetic Mapping of a Bacterial Blight Resistance Gene Xa14 in Rice: Genetic Mapping of a Bacterial Blight Resistance Gene Xa14 in Rice. Acta Agronomica Sinica **36,** 422-427.

Busungu, C., Taura, S., Sakagami, J.-I., and Ichitani, K. (2016). Identification and linkage analysis of a new rice bacterial blight resistance gene from XM14, a mutant line from IR24. Breeding Science **advpub**.

Busungu, C., Taura, S., Sakagami, J.-I., Anai, T., and Ichitani, K. (2018). High-resolution mapping and characterization of xa42, a resistance gene against multiple Xanthomonas oryzae pv. oryzae races in rice (Oryza sativa L.). Breeding science **68,** 188-199.

Cheema, K.K., Grewal, N.K., Vikal, Y., Sharma, R., Lore, J.S., Das, A., Bhatia, D., Mahajan, R., Gupta, V., Bharaj, T.S., and Singh, K. (2008). A novel bacterial blight resistance gene from Oryza nivara mapped to 38 kb region on chromosome 4L and transferred to Oryza sativa L. Genetics Research **90,** 397-407.

Chen, H., Wang, S., and Zhang, Q. (2002). New Gene for Bacterial Blight Resistance in Rice Located on Chromosome 12 Identified from Minghui 63, an Elite Restorer Line. Phytopathology® **92,** 750-754.

Chen, S., Liu, X., Zeng, L., Ouyang, D., Yang, J., and Zhu, X. (2011). Genetic analysis and molecular mapping of a novel recessive gene xa34(t) for resistance against Xanthomonas oryzae pv. oryzae. Theoretical and Applied Genetics **122,** 1331-1338.

Chen, S., Wang, C., Yang, J., Chen, B., Wang, W., Su, J., Feng, A., Zeng, L., and Zhu, X. (2020). Identification of the novel bacterial blight resistance gene Xa46(t) by mapping and expression analysis of the rice mutant H120. Scientific Reports **10,** 12642.

Chen, X., Liu, P., Mei, L., He, X., Chen, L., Liu, H., Shen, S., Ji, Z., Zheng, X., Zhang, Y., Gao, Z., Zeng, D., Qian, Q., and Ma, B. (2021). Xa7, a new executor R gene that confers durable and broad-spectrum resistance to bacterial blight disease in rice. Plant Communications **2,** 100143.

Chu, Z., Fu, B., Yang, H., Xu, C., Li, Z., Sanchez, A., Park, Y.J., Bennetzen, J.L., Zhang, Q., and Wang, S. (2006). Targeting xa13, a recessive gene for bacterial blight resistance in rice. Theoretical and Applied Genetics **112,** 455-461.

Deng, Y., Liu, H., Zhou, Y., Zhang, Q., Li, X., and Wang, S. (2018). Exploring the mechanism and efficient use of a durable gene-mediated resistance to bacterial blight disease in rice. Molecular Breeding **38,** 18.

Gao, D.-Y., Liu, A.-M., Zhou, Y.-H., Cheng, Y.-J., Xiang, Y.-H., Sun, L.-H., and Zhai, W.-X. (2005). Molecular mapping of a bacterial blight resistance gene Xa-25 in rice. Yi chuan xue bao = Acta genetica Sinica **32,** 183-188.

Gao, D.Y., Xu, Z.G., Chen, Z.Y., Sun, L.H., Sun, Q.M., Lu, F., Hu, B.S., and Liu, Y.F. (2002). Identification of a resistance gene to bacterial blight (Xanthomonas oryzae pv. oryzae) in a somaclonal mutant HX-3 of indica rice. Yi Chuan Xue Bao **29,** 138-143.

Goto, T., Matsumoto, T., Furuya, N., Tsuchiya, K., and Yoshimura, A. (2009). Mapping of Bacterial Blight Resistance Gene Xa11 on Rice Chromosome 3. Japan Agricultural Research Quarterly: JARQ **43,** 221-225.

Gu, K., Sangha, J.S., Li, Y., and Yin, Z. (2008). High-resolution genetic mapping of bacterial blight resistance gene Xa10. Theoretical and Applied Genetics **116,** 155-163.

Gu, K., Tian, D., Yang, F., Wu, L., Sreekala, C., Wang, D., Wang, G.L., and Yin, Z. (2004). High-resolution genetic mapping of Xa27(t), a new bacterial blight resistance gene in rice, Oryza sativa L. Theoretical and Applied Genetics **108,** 800-807.

Guo, S., Zhang, D., and Lin, X. (2010). Identification and mapping of a novel bacterial blight resistance gene *Xa35(t)* originated from *Oryza minuta*. Scientia Agricultura Sinica **43,** 2611-2618.

He, Q., Li, D., Zhu, Y., Tan, M., Zhang, D., and Lin, X. (2006). Fine Mapping of Xa2, a Bacterial Blight Resistance Gene in Rice. Molecular Breeding **17,** 1-6.

He, Z. (2021). Subspecies-specific domestication of an NLR immune receptor and reconstruction of broad-spectrum disease resistance in rice. In MOLECULAR PLANT-MICROBE INTERACTIONS (AMER PHYTOPATHOLOGICAL SOC 3340 PILOT KNOB ROAD, ST PAUL, MN 55121 USA).

Hisatoshi, K. (1993). Infection Types in Rice-Xanthomonas campestris pv. oryzae Interaction. Japan Agricultural Research Quarterly **27,** 81-87.

Hu, K., Cao, J., Zhang, J., Xia, F., Ke, Y., Zhang, H., Xie, W., Liu, H., Cui, Y., Cao, Y., Sun, X., Xiao, J., Li, X., Zhang, Q., and Wang, S. (2017). Improvement of multiple agronomic traits by a disease resistance gene via cell wall reinforcement. Nature Plants **3,** 17009.

Hutin, M., Sabot, F., Ghesquière, A., Koebnik, R., and Szurek, B. (2015). A knowledge-based molecular screen uncovers a broad-spectrum OsSWEET14 resistance allele to bacterial blight from wild rice. The Plant Journal **84,** 694-703.

Iyer, A.S., and McCouch, S.R. (2004). The Rice Bacterial Blight Resistance Gene xa5 Encodes a Novel Form of Disease Resistance. Molecular Plant-Microbe Interactions® **17,** 1348-1354.

Ji, C., Ji, Z., Liu, B., Cheng, H., Liu, H., Liu, S., Yang, B., and Chen, G. (2020). Xa1 Allelic R Genes Activate Rice Blight Resistance Suppressed by Interfering TAL Effectors. Plant communications **1,** 100087-100087.

Jin, X.W., Wang, C., Yang, Q., Jiang, Q.X., Fan, Y.L., Liu, G.C., and Zhao, K. (2007). Breeding of near-isogenic line CBB30 and molecular mapping of Xa30(t), a new resistance gene to bacterial blight in rice. Sci Agric Sin **40,** 1094-1100.

Kaku, H., and Ogawa, T. (2000). The relationship between browning reaction and bacterial blight resistance gene Xa3 in rice.

Kaku, H., and Ogawa, T. (2001). Genetic Analysis of the Relationship between the Browning Reaction and Bacterial Blight Resistance Gene Xa3 in Rice. Journal of General Plant Pathology **67,** 228-230.

Khush, G.S., Bacalangco, E., and Ogawa, T. (1990). 18. A new gene for resistance to bacterial blight from O. longistaminata. Rice Genet. News Lett **7,** 121-122.

Kim, S.-M. (2018). Identification of novel recessive gene xa44(t) conferring resistance to bacterial blight races in rice by QTL linkage analysis using an SNP chip. Theoretical and Applied Genetics **131,** 2733-2743.

Kim, S.-M., and Reinke, R.F. (2019). A novel resistance gene for bacterial blight in rice, Xa43(t) identified by GWAS, confirmed by QTL mapping using a bi-parental population. PloS one **14,** e0211775-e0211775.

Kim, S.-M., Suh, J.-P., Qin, Y., Noh, T.-H., Reinke, R.F., and Jena, K.K. (2015). Identification and fine-mapping of a new resistance gene, Xa40, conferring resistance to bacterial blight races in rice (Oryza sativa L.). Theoretical and Applied Genetics **128,** 1933-1943.

Korinsak, S., Sriprakhon, S., Sirithanya, P., Jairin, J., Korinsak, S., Vanavichit, A., and Toojinda, T. (2009). Identification of microsatellite markers (SSR) linked to a new bacterial blight resistance gene xa33(t) in rice cultivar 'Ba7'. Maejo international journal of science and technology **3,** 235-247.

Kumar, P.N., Sujatha, K., Laha, G.S., Rao, K.S., Mishra, B., Viraktamath, B.C., Hari, Y., Reddy, C.S., Balachandran, S.M., Ram, T., Madhav, M.S., Rani, N.S., Neeraja, C.N., Reddy, G.A., Shaik, H., and Sundaram, R.M. (2012). Identification and Fine-Mapping of Xa33, a Novel Gene for Resistance to Xanthomonas oryzae pv. oryzae. Phytopathology® **102,** 222-228.

Lee, K., Rasabandith, S., Angeles, E.R., and Khush, G. (2003). Inheritance of Resistance to Bacterial Blight in 21 Cultivars of Rice. Phytopathology **93,** 147-152.

Li, Z.K., Sanchez, A., Angeles, E., Singh, S., Domingo, J., Huang, N., and Khush, G.S. (2001). Are the dominant and recessive plant disease resistance genes similar? A case study of rice R genes and Xanthomonas oryzae pv. oryzae races. Genetics **159,** 757-765.

Liang, L.Q., Wang, C.Y., Zeng, L.X., Wang, W.J., Feng, J.Q., Chen, B., Su, J., Chen, S., Shang, F.D., Zhu, X.Y., and Lin, F. (2017). The rice cultivar Baixiangzhan harbours a recessive gene xa42(t) determining resistance against Xanthomonas oryzae pv. oryzae. Plant Breeding **136,** 603-609.

Lin, X., Zhang, D., Xie, Y., Gao, H., and Zhang, Q. (1996). Identifying and mapping a new gene for bacterial blight resistance in rice based on RFLP markers. Phytopathology **86,** 1156-1159.

Liu, Q., Yuan, M., Zhou, Y., Li, X., Xiao, J., and Wang, S. (2011). A paralog of the MtN3/saliva family recessively confers race-specific resistance to Xanthomonas oryzae in rice. Plant, Cell & Environment **34,** 1958-1969.

Lu, Y., Zhong, Q., Xiao, S., Wang, B., Ke, X., Zhang, Y., Yin, F., Zhang, D., Jiang, C., Liu, L., Li, J., Yu, T., Wang, L., Cheng, Z., and Chen, L. (2022). A new NLR disease resistance gene Xa47 confers durable and broad-spectrum resistance to bacterial blight in rice. Frontiers in plant science **13,** 1037901-1037901.

Miao, L., Wang, C., Zheng, C., Che, J., Gao, Y., Wen, Y., Li, G., and Zhao, K. (2010). Molecular mapping of a new gene for resistance to rice bacterial blight. Scientia Agricultura Sinica **43,** 3051-3058.

Msami, J.A., Kawaguchi, Y., Ichitani, K., and Taura, S. (2021). Linkage analysis of rice bacterial blight resistance gene xa20 in XM6, a mutant line from IR24. Breeding science **71,** 144-154.

Nakai, H., Nakamura, K., Kuwahara, S., and Saito, M. (1988). Genetic studies of an induced rice mutant resistant to multiple races of bacterial leaf blight. Rice Genetics Newsletter **5,** 101.

Neelam, K., Mahajan, R., Gupta, V., Bhatia, D., Gill, B.K., Komal, R., Lore, J.S., Mangat, G.S., and Singh, K. (2020). High-resolution genetic mapping of a novel bacterial blight resistance gene xa-45(t) identified from Oryza glaberrima and transferred to Oryza sativa. Theoretical and Applied Genetics **133,** 689-705.

Noda, T., and Ohuchi, A. (1989). A New Pathogenic Race of *Xanthomonas campestris* pv. *oryzae* and Inheritance of Resistance of Differential Rice Variety, Te-tep to It. Japanese Journal of Phytopathology **55,** 201-207.

Ogawa, T., Kaku, H., and Yamamoto, T. (1989). Resistance gene of rice cultivar, Asaminori to bacterial blight of rice. Jpn J Breed **39,** 196-197.

OGAWA, T., MORINAKA, T., FUJII, K., and KIMURA, T. (1978). Inheritance of resistance of rice varieties Kogyoku and Java 14 to bacterial group V of Xanthomonas oryzae. Japanese Journal of Phytopathology **44,** 137-141.

Porter, B.W., Chittoor, J.M., Yano, M., Sasaki, T., and White, F.F. (2003). Development and Mapping of Markers Linked to the Rice Bacterial Blight Resistance Gene Xa7. Crop Science **43,** 1484-1492.

Ram, T., Laha, G.s., Gautam, S., Deen, R., Sheshu madhav, M., Brar, D., and Viraktamath, B. (2010). Identification of a new gene from Oryza brachyantha with broad-spectrum resistance to bacterial blight of rice in India. Rice Genet. Newsl. **25**.

Ronald, P.C., Albano, B., Tabien, R., Abenes, L., Wu, K.-s., McCouch, S., and Tanksley, S.D. (1992). Genetic and physical analysis of the rice bacterial blight disease resistance locus, Xa21. Molecular and General Genetics MGG **236,** 113-120.

Ruan, H.-H., Yan, C.-Q., An, D.-R., Liu, R.-H., and Chen, J.-P. (2008). Identifying and mapping new gene xa32 (t) for resistance to bacterial blight (Xanthomonas oryzae pv. oryzae, Xoo) from Oryza meyeriana L. Acta Agricult Boreali-occidentalis Sin **17,** 170-174.

Sidhu, G.S., and Khush, G.S. (1978). Dominance reversal of a bacterial blight resistance gene in some rice cultivars. Phytopathology **68,** 461.

Singh, R.J., Khush, G.S., and Mew, T.W. (1983). A New Gene for Resistance to Bacterial Blight in Rice. Crop Science **23,** cropsci1983.0011183X002300030026x.

Song, W.-Y., Wang, G.-L., Chen, L.-L., Kim, H.-S., Pi, L.-Y., Holsten, T., Gardner, J., Wang, B., Zhai, W.-X., Zhu, L.-H., Fauquet, C., and Ronald, P. (1995). A Receptor Kinase-Like Protein Encoded by the Rice Disease Resistance Gene, *Xa21*. Science **270,** 1804-1806.

Sun, X., Cao, Y., Yang, Z., Xu, C., Li, X., Wang, S., and Zhang, Q. (2004). Xa26, a gene conferring resistance to Xanthomonas oryzae pv. oryzae in rice, encodes an LRR receptor kinase-like protein. The Plant journal **37,** 517-527.

Tan, G.-X., Ren, X., Weng, Q.-M., Shi, Z.-Y., Zhu, L.-L., and He, G.-C. (2004). Mapping of a new resistance gene to bacterial blight in rice line introgressed from Oryza officinalis. Yi chuan xue bao = Acta genetica Sinica **31,** 724-729.

Taura, S., and Ichitani, K. (2023). Chromosomal Location of xa19, a Broad-Spectrum Rice Bacterial Blight Resistant Gene from XM5, a Mutant Line from IR24. Plants (Basel) **12,** 602.

Taura, S., Ogawa, T., Yoshimura, A., Ikeda, R., and Omura, T. (1991). Identification of a Recessive Resistance Gene in Induced Mutant Line XM5 of Rice to Rice Bacterial Blight. Japanese Journal of Breeding **41,** 427-432.

Taura, S., Ogawa, T., Yoshimura, A., Ikeda, R., and IWata, N. (1992). Identification of a Recessive Resistance Gene to Rice Bacterial Blight of Mutant Line XM6, Oryza sativa L. Japanese Journal of Breeding **42,** 7-13.

Taura, S., Ogawa, T., Tabien, R., Khush, G., Yoshimura, A., and Omura, T. (1987). The specific reaction of Taichung Native 1 to Philippine races of bacterial blight and inheritance of resistance to race 5 (Pxo112). Rice Genetics Newsletter **4,** 101-102.

Vera Cruz, C.M., Bai, J., Oña, I., Leung, H., Nelson, R.J., Mew, T.-W., and Leach, J.E. (2000). Predicting durability of a disease resistance gene based on an assessment of the fitness loss and epidemiological consequences of avirulence gene mutation. Proceedings of the National Academy of Sciences **97,** 13500-13505.

Vikal, Y., Chawla, H., Sharma, R., Lore, J.S., and Singh, K. (2014). Mapping of bacterial blight resistance gene xa8 in rice (Oryza sativa L.). INDIAN JOURNAL OF GENETICS AND PLANT BREEDING **74,** 589-595.

Vikal, Y., Das, A., Patra, B., Goel, R.K., Sidhu, J.S., and Singh, K. (2007). Identification of new sources of bacterial blight (Xanthomonas oryzae pv. oryzae) resistance in wild Oryza species and O. glaberrima. Plant Genetic Resources **5,** 108-112.

Wang, C.-L., Zhao, B., Zhang, Q., Zhao, K.-j., and Xing, Q.-d. (2004). Identification of a New Rice Germplasm with Resistance to Bacterial Blight and the Breeding of a Near-isogenic Line. Journal of Plant Genetic Resources **5,** 26-30.

Wang, C., Wen, G., Lin, X., Liu, X., and Zhang, D. (2009). Identification and fine mapping of the new bacterial blight resistance gene, Xa31(t), in rice. European Journal of Plant Pathology **123,** 235-240.

Wang, C., Tan, M., Xu, X., Wen, G., Zhang, D., and Lin, X. (2003). Localizing the Bacterial Blight Resistance Gene, Xa22(t), to a 100-Kilobase Bacterial Artificial Chromosome. Phytopathology® **93,** 1258-1262.

Wang, C., Qin, T., Yu, H., Zhang, X., Che, J., Gao, Y., Zheng, C., Yang, B., and Zhao, K. (2014). The broad bacterial blight resistance of rice line CBB23 is triggered by a novel transcription activator-like (TAL) effector of *Xanthomonas oryzae* pv. *oryzae*. Molecular plant pathology **15,** 333-341.

Wang, C., Zhang, X., Fan, Y., Gao, Y., Zhu, Q., Zheng, C., Qin, T., Li, Y., Che, J., Zhang, M., Yang, B., Liu, Y., and Zhao, K. (2015). XA23 Is an Executor R Protein and Confers Broad-Spectrum Disease Resistance in Rice. Molecular Plant **8,** 290-302.

Wang, W., Zhai, W., Luo, M., Jiang, G., Chen, X., Li, X., Wing, R.A., and Zhu, L. (2001). Chromosome landing at the bacterial blight resistance gene Xa4 locus using a deep coverage rice BAC library. Molecular Genetics and Genomics **265,** 118-125.

Wu, X., Li, X., Xu, C., and Wang, S. (2008). Fine genetic mapping of xa24, a recessive gene for resistance against Xanthomonas oryzae pv. oryzae in rice. Theoretical and Applied Genetics **118,** 185-191.

**Xiang, Y., Cao, Y., Xu, C., Li, X., and Wang, S.** (2006). Xa3, conferring resistance for rice bacterial blight and encoding a receptor kinase-like protein, is the same as Xa26. Theoretical and Applied Genetics **113,** 1347-1355.
**Xing, J., Zhang, D., Yin, F., Zhong, Q., Wang, B., Xiao, S., Ke, X., Wang, L., Zhang, Y., Zhao, C., Lu, Y., Chen, L., Cheng, Z., and Chen, L.** (2021). Identification and Fine-Mapping of a New Bacterial Blight Resistance Gene, Xa47(t), in G252, an Introgression Line of Yuanjiang Common Wild Rice (Oryza rufipogon). Plant Disease **105,** 4106-4112.
**Yamada, T.** (1984). Multiple allele at the Xa-1 and Xa-kg loci for resistance to bacterial leaf blight. Rice Genet. Newsl **1,** 97-98.
**Yamamoto, T., and Ogawa, T.** (1990). Inheritance of resistance in rice cultivars, Toyonishiki, Milyang 23 and IR24 to Myanmar isolates of bacterial leaf blight pathogen. JARQ **24,** 74-77.
**Yogesh, V., and Dharminder, B.** (2017). Genetics and Genomics of Bacterial Blight Resistance in Rice. In Advances in International Rice Research, L. Jinquan, ed (Rijeka: IntechOpen), pp. Ch. 10.
**Yoshimura, S., Yamanouchi, U., Katayose, Y., Toki, S., Wang, Z.-X., Kono, I., Kurata, N., Yano, M., Iwata, N., and Sasaki, T.** (1998). Expression of *Xa1*, a bacterial blight-resistance gene in rice, is induced by bacterial inoculation. Proceedings of the National Academy of Sciences **95,** 1663-1668.
**Zeng, L., Cheng, T., and Zhu, X.** (2009). Breeding of rice inbred cultivar Baixiangzhan first reported resistance to rice bacterial blight pathogen type V severe virulent strain in Guangdong. Guangdong Agricultural Sciences **5,** 19, 28.
**Zhang, F., Zhuo, D.-L., Zhang, F., Huang, L.-Y., Wang, W.-S., Xu, J.-L., Vera Cruz, C., Li, Z.-K., and Zhou, Y.-L.** (2015). Xa39, a novel dominant gene conferring broad-spectrum resistance to Xanthomonas oryzae pv. oryzae in rice. Plant Pathology **64,** 568-575.
**Zhang, Q., Wang, C., Zhao, K., Zhao, Y., Caslana, V., Zhu, X., Li, D., and Jiang, Q.** (2001). The effectiveness of advanced rice lines with new resistance gene Xa23 to rice bacterial blight. Rice Genetics Newsletter **18,** 71-72.
**Zheng, C.-K., Wang, C.-L., Yu, Y.-J., Liang, Y.-T., and Zhao, K.-J.** (2009). Identification and Molecular Mapping of Xa32(t), a Novel Resistance Gene for Bacterial Blight (Xanthomonas oryzae pv. oryzae) in Rice. Acta Agronomica Sinica **35,** 1173-1180.
