# Supplementary material for: Current status of molecular rice breeding for durable and broad-spectrum resistance to major diseases and insect pests
Source: Theor Appl Genet. 2024 Sep 10;137(10):219. doi: 10.1007/s00122-024-04729-3 (PMC11387466; doi:10.1007/s00122-024-04729-3)
Supplement: Supplementary file 4 — Supplementary file4 (PDF 257 KB) [file 122_2024_4729_MOESM4_ESM.pdf]

Supplemental Table 4. Details on brown planthopper resistance genes/Loci in rice

Gene	Germplasm source	Chromosome	Resistance to biotypes				Resistance to other insects	References
			Biotype1	Biotype2	Biotype3	Biotype4		
BPH1	Mudgo	12	R	S	R	S		(Khush and Brar, 1991; Jena and Kim, 2010)
bph2	ASD7	12	R	R	S	S		(Khush and Brar, 1991; Young-Soon et al., 2008; Jena and Kim,
BPH3	Rathu Heenati	4	R	R	R	R	WBPH	(Khush and Brar, 1991; Jairin et al., 2007a; Jena and Kim, 2010; Liu et al., 2015)
bph4	Babawee	6	R	R	R	R		(Khush and Brar, 1991; Jena and Kim, 2010)
bph5	ARC 10550	ND	S	S	S	R		(Khush and Brar, 1991; Jena and Kim, 2010)
BPH6	Swarnalata	4	R	R	R	R	WBPH	(Khush and Brar, 1991; Jena and Kim, 2010; Guo et al., 2018)
bph7	T12	12	S	R	S	R		(Khush and Brar, 1991; Jena and Kim, 2010; Qiu et al., 2014)
bph8	Chin Saba	ND	R	R	R	ND		(Khush and Brar, 1991; Jena and Kim, 2010)
BPH9	Pokkali	12	R	R	R	ND		(Murata et al., 2001; Zhao et al., 2016)
BPH9	Kaharamana	12	R	ND	ND	ND		(Su et al., 2006)
BPH9	Balamawee	12	R	R	R	ND		(Khush and Brar, 1991; Jena and Kim, 2010)
BPH10	*O.australiensis*	12	R	R	R	ND		(Ishii et al., 1994)
bph11	*O.officinalis*	3	ND	ND	ND	ND		(Hirabayashi et al. 1998)
BPH12	B14 (*O.latifolia*)	4	ND	R	ND	ND		(Jena and Kim, 2010; Qiu et al., 2012)
BPH13	*O.officinalis*	3	ND	ND	ND	R		(Renganayaki et al. 2002)
BPH14	B5 (*O.officinalis*)	3	ND	ND	ND	ND		(Du et al., 2009)
BPH15	B5 (*O.officinalis*)	4	ND	ND	ND	ND		(Yang et al., 2004)
BPH16	M1635–7	12	ND	ND	ND	ND		(Hirabayashi et al., 2004)
BPH17	Rathu Heenati	4	R	R	ND	ND		(Sun et al., 2005)
BPH18	IR65482–7–216-1-2(*O. australiensis*	12	R	R	R	R		(Jena and Kim, 2010)
bph18(t)	*O. rufipogon*	4	R	R	ND	ND		(Li et al., 2006)
bph19(t)	AS20–1	3	ND	R	ND	ND		(Chen et al. 2006)
bph19(t)	*O. rufipogon*	12	R	R	ND	ND		(Li et al., 2006)
BPH20(t)	IR71033–121-15 (*O. miniuta*)	4	ND	ND	ND	ND		(Rahman et al., 2009)
BPH21(t)	IR71033–121-15 (*O. miniuta*)	12	ND	ND	ND	ND		(Rahman et al., 2009)
BPH22(t)	*O. glaberrima*	ND	ND	ND	ND	R		(Ram et al., 2010)
BPH23(t)	*O. minuta*	ND	ND	ND	ND	R		(Ram et al., 2010)
bph22(t)	*O. rufipogon*	4	R	R	ND	ND		(Hou et al., 2011)
bph23(t)	*O. rufipogon*	8	R	R	ND	ND		(Hou et al., 2011)
bph24(t)	IR73678–6-9-B (*O. rufipogon*)	ND	ND	ND	ND	R		(Deen et al., 2010)
bph25	ADR52	6	ND	ND	ND	ND	WBPH	(Myint et al., 2012)
BPH26	ADR52	12	ND	ND	ND	ND		(Myint et al., 2012; Tamura et al., 2014)
BPH27	*GX2183 (O. rufipogon)*	4	ND	R	ND	ND		(Huang et al., 2013)
BPH27(t)	Balamawee	4	ND	ND	ND	ND		(He et al., 2013)
BPH28(t)	DV85	11	ND	ND	ND	ND		(Wu et al., 2014)
bph29	RBPH54 (*O. rufipogon*)	6	ND	R	ND	ND		(Wang et al., 2015)
BPH30	AC-1613	4	R	R	R	ND	WBPH	(Shi et al., 2021)
BPH31	CR2711–76	3	R	R	R	R		(Prahalada et al., 2017)
BPH32	Ptb33	6	ND	ND	ND	ND		(Ren et al., 2016)
BPH33	KOLAYAL, POLIYAL	4	ND	ND	ND	ND		(Hu et al., 2018)
BPH33(t)	RP2068	1	ND	ND	ND	ND		(Naik et al., 2018)
BPH34	IRGC104646 (*O. nivara*)	4	ND	ND	ND	R		(Kumar et al., 2018)
BPH35	RBPH660 (*O.rufipogon*)	4	ND	ND	ND	ND		(Zhang et al., 2020)
BPH36	GX2183 (*O.rufipogon*)	4	ND	ND	ND	ND		(Li et al., 2019)
BPH37	IR64	1	ND	R	ND	ND		(Yang et al, 2019)
BPH38(t)	Khazar	1	ND	ND	R	ND		(Balachiranjeevi et al., 2019)
bph39(t)	IRGC81848 (*O.nivara*)	ND	ND	ND	ND	R		(Akanksha et al., 2019)
bph40(t)	IRGC81848 (*O.nivara*)	ND	ND	ND	ND	R		(Akanksha et al., 2019)
BPH41	SWD10	4	ND	ND	ND	R		(Tan et al., 2022)
BPH42	SWD10	4	ND	ND	ND	R		(Tan et al., 2022)
bph42	CR100441 (*O. rufipogon*)	4	ND	ND	ND	R		(Kaur et al., 2022)
BPH43	IRGC 8678.	11	R	ND	R	ND		(Kim et al., 2022)
BPH44	Balamawee	4	ND	ND	ND	ND		(Kiswanto et al., 2022)
BPH45	IRGC102165 (O. nivara)	4	R	ND	ND	ND		(Li et al., 2023)
qBPH3	IR02W101 (*O.officinalis*)	3	ND	R	ND	ND		(Hu et al., 2015a)
qBPH4	IR02W101 (*O.officinalis*)	4	ND	R	ND	ND		(Hu et al., 2015a)
qBPH4.2	IR65482–17-511(*O. australiensis*)	4	ND	ND	ND	ND		(Hu et al., 2015b)
qBPH4.3	Salkathi	4	ND	ND	ND	R		(Mohanty et al., 2017)
qBPH4.4	Salkathi	4	ND	ND	ND	R		(Mohanty et al., 2017)
qBPH6(t)	IR71033-121-15 (*O. miniuta*)	6	ND	ND	ND	ND	yellow stem borer, WBPH, green leafhopper and gall midge	(Jairin et al., 2007b)

R: resistance; S: susceptibility; ND: no data; white-backed planthopper: WBPH

References

Akanksha, S., Jhansi Lakshmi, V., Singh, A.K., Deepthi, Y., Chirutkar, P.M., Ramdeen, Balakrishnan, D., Sarla, N., Mangrauthia, S.K., and Ram, T. (2019). Genetics of novel brown planthopper *Nilaparvata lugens* (Stål) resistance genes in derived introgression lines from the interspecific cross *O. sativa* var. Swarna × O. nivara. Journal of genetics **98**.

Balachiranjeevi, C.H., Prahalada, G.D., Mahender, A., Jamaloddin, M., Sevilla, M.A.L., Marfori-Nazarea, C.M., Vinarao, R., Sushanto, U., Baehaki, S.E., Li, Z.K., and Ali, J. (2019). Identification of a novel locus, BPH38(t), conferring resistance to brown planthopper (Nilaparvata lugens Stal.) using early backcross population in rice (Oryza sativa L.). Euphytica **215,** 185-185.

Chen, J.W., Wang, L., Pang, X.F., and Pan, Q.H. (2006). Genetic analysis and fine mapping of a rice brown planthopper (Nilaparvata lugens Stål) resistance gene bph19(t). Molecular Genetics and Genomics **275,** 321-329.

Deen, R., Ramesh, K., Gautam, S., Rao, Y., Lakshmi, V., Viraktamath, B., Brar, D., and Ram, T. (2010). Identification of new gene for BPH resistance introgressed from *O. rufipogon*. Rice Genetics Newsletter **25,** 70-71.

Du, B., Zhang, W., Liu, B., Hu, J., Wei, Z., Shi, Z., He, R., Zhu, L., Chen, R., Han, B., and He, G. (2009). Identification and characterization of *Bph14*, a gene conferring resistance to brown planthopper in rice. Proceedings of the National Academy of Sciences of the United States of America **106,** 22163-22168.

Guo, J., Xu, C., Wu, D., Zhao, Y., Qiu, Y., Wang, X., Ouyang, Y., Cai, B., Liu, X., Jing, S., Shangguan, X., Wang, H., Ma, Y., Hu, L., Wu, Y., Shi, S., Wang, W., Zhu, L., Xu, X., Chen, R., Feng, Y., Du, B., and He, G. (2018). Bph6 encodes an exocyst-localized protein and confers broad resistance to planthoppers in rice. Nature Genetics **50,** 297-306.

He, J., Liu, Y., Liu, Y., Jiang, L., Wu, H., Kang, H., Liu, S., Chen, L., Liu, X., Cheng, X., and Wan, J. (2013). High-resolution mapping of brown planthopper (BPH) resistance gene *Bph27(t)* in rice *(Oryza sativa* L.). Molecular Breeding **31,** 549-557.

Hirabayashi, H., Ideta, O., Sato, H., Takeuchi, Y., Ando, I., Nemoto, H., Imbe, T., Brar, D., and Ogawa, T. (2004). Identification of a resistance gene to brown planthopper derived from *Oryza minuta* in rice. Breeding Research **6,** 285.

Hou, L.-y., Yu, P., Xu, Q., Yuan, X.-p., Yu, H.-y., Wang, Y.-p., Wang, C.-h., Wan, G., Tang, S.-x., Peng, S.-t., and Wei, X.-h. (2011). Genetic Analysis and Preliminary Mapping of Two Recessive Resistance Genes to Brown Planthopper, *Nilaparvata lugens* Stål in Rice. Rice Science **18,** 238-242.

Hu, J., Chang, X., Zou, L., Tang, W., and Wu, W. (2018). Identification and fine mapping of Bph33, a new brown planthopper resistance gene in rice (Oryza sativa L.). Rice **11,** 55.

Hu, J., Xiao, C., Cheng, M., Gao, G., Zhang, Q., and He, Y. (2015a). Fine mapping and pyramiding of brown planthopper resistance genes QBph3 and QBph4 in an introgression line from wild rice O. officinalis. Molecular Breeding **35,** 3.

Hu, J., Xiao, C., Cheng, M.-x., Gao, G.-j., Zhang, Q.-l., and He, Y.-q. (2015b). A new finely mapped Oryza australiensis-derived QTL in rice confers resistance to brown planthopper. Gene **561,** 132-137.

Huang, D., Qiu, Y., Zhang, Y., Huang, F., Meng, J., Wei, S., Li, R., and Chen, B. (2013). Fine mapping and characterization of *BPH27*, a brown planthopper resistance gene from wild rice (*Oryza rufipogon* Griff.). Theoretical and Applied Genetics **126,** 219-229.

Ishii, T., Brar, D.S., Multani, D.S., and Khush, G.S. (1994). Molecular tagging of genes for brown planthopper resistance and earliness introgressed from *Oryza australiensis* into cultivated rice, *O. sativa*. Genome **37,** 217-221.

Jairin, J., Phengrat, K., Teangdeerith, S., Vanavichit, A., and Toojinda, T. (2007a). Mapping of a broad-spectrum brown planthopper resistance gene, Bph3, on rice chromosome 6. Molecular Breeding **19,** 35-44.

Jairin, J., Teangdeerith, S., Leelagud, P., Phengrat, K., Vanavichit, A., and Toojinda, T. (2007b). Detection of Brown Planthopper Resistance Genes from Different Rice Mapping Populations in the Same Genomic Location. ScienceAsia **33,** 347-352.

Jena, K.K., and Kim, S.-M. (2010). Current Status of Brown Planthopper (BPH) Resistance and Genetics. Rice **3,** 161-171.

Kaur, P., Neelam, K., Sarao, P.S., Babbar, A., Kumar, K., Vikal, Y., Khanna, R., Kaur, R., Mangat, G.S., and Singh, K. (2022). Molecular mapping and transfer of a novel brown planthopper resistance gene bph42 from Oryza rufipogon (Griff.) To cultivated rice (Oryza sativa L.). Molecular Biology Reports **49,** 8597-8606.

Khush, G.S., and Brar, D.S. (1991). Genetics Of Resistance To Insects In Crop Plants. In Advances in Agronomy, N.C. Brady, ed (Academic Press), pp. 223-274.

Kim, J., An, X., Yang, K., Miao, S., Qin, Y., Hu, Y., Du, B., Zhu, L., He, G., and Chen, R. (2022). Molecular Mapping of a New Brown Planthopper Resistance Gene *Bph43* in Rice (*Oryza sativa* L.). Agronomy **12,** 808.

Kiswanto, I., Soetopo, L., and Adiredjo, A.L. (2022). Identification of novel candidate of brown planthopper resistance gene Bph44 in rice (Oryza sativa L.). Genome **65,** 505-511.

Kumar, K., Sarao, P.S., Bhatia, D., Neelam, K., Kaur, A., Mangat, G.S., Brar, D.S., and Singh, K. (2018). High-resolution genetic mapping of a novel brown planthopper resistance locus, *Bph34* in *Oryza sativa* L. X *Oryza nivara* (Sharma & Shastry) derived interspecific F2 population. Theoretical and Applied Genetics **131,** 1163-1171.

Li, C.-P., Wu, D.-H., Huang, S.-H., Meng, M., Shih, H.-T., Lai, M.-H., Chen, L.-J., Jena, K.K., Hechanova, S.L., Ke, T.-J., Chiu, T.-Y., Tsai, Z.-Y., Chen, G.-K., Tsai, K.-C., and Leu, W.-M. (2023). The Bph45 Gene Confers Resistance against Brown Planthopper in Rice by Reducing the Production of Limonene. International Journal of Molecular Sciences **24,** 1798.

Li, R., Li, L., Wei, S., Wei, Y., Chen, Y., Bai, D., Yang, L., Huang, F., Lu, W., Zhang, X., Li, X., Yang, X., and Wei, Y. (2006). The evaluation and utilization of new genes for brown planthopper resistance in common wild rice (*Oryza rufipogon* Griff.). Molecular Plant Breeding **14,** 365-371.

Li, Z., Xue, Y., Zhou, H., Li, Y., Usman, B., Jiao, X., Wang, X., Liu, F., Qin, B., Li, R., and Qiu, Y. (2019). High-resolution mapping and breeding application of a novel brown planthopper resistance gene derived from wild rice (*Oryza. rufipogon Griff*). Rice **12,** 41.

Liu, Y., Wu, H., Chen, H., Liu, Y., He, J., Kang, H., Sun, Z., Pan, G., Wang, Q., Hu, J., Zhou, F., Zhou, K., Zheng, X., Ren, Y., Chen, L., Wang, Y., Zhao, Z., Lin, Q., Wu, F., Zhang, X., Guo, X., Cheng, X., Jiang, L., Wu, C., Wang, H., and Wan, J. (2015). A gene cluster encoding lectin receptor kinases confers broad-spectrum and durable insect resistance in rice. Nature Biotechnology **33,** 301-305.

Mohanty, S.K., Panda, R.S., Mohapatra, S.L., Nanda, A., Behera, L., Jena, M., Sahu, R.K., Sahu, S.C., and Mohapatra, T. (2017). Identification of novel quantitative trait loci associated with brown planthopper resistance in the rice landrace Salkathi. Euphytica **213,** 38.

Murata, K., Fujiwara, M., Murai, H., Takumi, S., Mori, N., and Nakamura, C. (2001). Mapping of a brown planthopper (*Nilaparvata lugens* Stål) resistance gene *Bph9* on the long arm of rice chromosome 12. Cereal Research Communications **29,** 245-250.

Myint, K.K.M., Fujita, D., Matsumura, M., Sonoda, T., Yoshimura, A., and Yasui, H. (2012). Mapping and pyramiding of two major genes for resistance to the brown planthopper (Nilaparvata lugens [Stål]) in the rice cultivar ADR52. Theoretical and Applied Genetics **124,** 495-504.

Prahalada, G.D., Shivakumar, N., Lohithaswa, H.C., Sidde Gowda, D.K., Ramkumar, G., Kim, S.-R., Ramachandra, C., Hittalmani, S., Mohapatra, T., and Jena, K.K. (2017). Identification and fine mapping of a new gene, BPH31 conferring resistance to brown planthopper biotype 4 of India to improve rice, Oryza sativa L. Rice **10,** 41.

Qiu, Y., Guo, J., Jing, S., Zhu, L., and He, G. (2012). Development and characterization of japonica rice lines carrying the brown planthopper-resistance genes BPH12 and BPH6. Theoretical and Applied Genetics **124,** 485-494.

Qiu, Y., Guo, J., Jing, S., Zhu, L., and He, G. (2014). Fine mapping of the rice brown planthopper resistance gene *BPH7* and characterization of its resistance in the 93-11 background. Euphytica **198,** 369-379.

Rahman, M.L., Jiang, W., Chu, S.H., Qiao, Y., Ham, T.-H., Woo, M.-O., Lee, J., Khanam, M.S., Chin, J.-H., Jeung, J.-U., Brar, D.S., Jena, K.K., and Koh, H.-J. (2009). High-resolution mapping of two rice brown planthopper resistance genes, Bph20(t) and Bph21(t), originating from Oryza minuta. Theoretical and Applied Genetics **119,** 1237-1246.

Ram, T., Deen, R., Gautam, S.K., Kandu, R., Rao, Y.K., and Brar, D.S. (2010). Identification of new genes for brown planthopper resistance in rice introgressed from *O. glaberrima* and *O. minuta*. Rice Genetics Newsletter **25,** 67-69.

Ren, J., Gao, F., Wu, X., Lu, X., Zeng, L., Lv, J., Su, X., Luo, H., and Ren, G. (2016). Bph32, a novel gene encoding an unknown SCR domain-containing protein, confers resistance against the brown planthopper in rice. Scientific Reports **6,** 37645.

Renganayaki, K., Fritz, A.K., Sadasivam, S., Pammi, S., Harrington, S.E., McCouch, S.R., Kumar, S.M., and Reddy, A.S. (2002). Mapping and Progress toward Map-Based Cloning of Brown Planthopper Biotype-4 Resistance Gene Introgressed from Oryza officinalis into Cultivated Rice, O. sativa. Crop Science **42,** 2112-2117.

Shi, S., Wang, H., Nie, L., Tan, D., Zhou, C., Zhang, Q., Li, Y., Du, B., Guo, J., Huang, J., Wu, D., Zheng, X., Guan, W., Shan, J., Zhu, L., Chen, R., Xue, L., Walling, L.L., and He, G. (2021). Bph30 confers resistance to brown planthopper by fortifying sclerenchyma in rice leaf sheaths. Molecular Plant **14,** 1714-1732.

Su, C.-C., Zhai, H.-Q., Wang, C.-M., Sun, L.-H., and Wan, J.-M. (2006). SSR Mapping of Brown Planthopper Resistance Gene *Bph9* in Kaharamana, an Indica Rice (*Oryza sativa* L.). Acta Genetica Sinica **33,** 262-268.

Sun, L., Su, C., Wang, C., Zhai, H.-q., and Wan, J.J.B.S. (2005). Mapping of a Major Resistance Gene to the Brown Planthopper in the Rice Cultivar Rathu Heenati. Breeding Science **55,** 391-396.

Tamura, Y., Hattori, M., Yoshioka, H., Yoshioka, M., Takahashi, A., Wu, J., Sentoku, N., and Yasui, H. (2014). Map-based Cloning and Characterization of a Brown Planthopper Resistance Gene BPH26 from Oryza sativa L. ssp. indica Cultivar ADR52. Scientific Reports **4,** 5872.

Tan, H.Q., Palyam, S., Gouda, J., Kumar, P.P., and Chellian, S.K. (2022). Identification of two QTLs, BPH41 and BPH42, and their respective gene candidates for brown planthopper resistance in rice. Scientific Reports **12,** 18538.

Wang, Y., Cao, L., Zhang, Y., Cao, C., Liu, F., Huang, F., Qiu, Y., Li, R., and Luo, X. (2015). Map-based cloning and characterization of BPH29, a B3 domain-containing recessive gene conferring brown planthopper resistance in rice. Journal of Experimental Botany **66,** 6035-6045.

Wu, H., Liu, Y., He, J., Liu, Y., Jiang, L., Liu, L., Wang, C., Cheng, X., and Wan, J. (2014). Fine mapping of brown planthopper (*Nilaparvata lugens* Stål) resistance gene *Bph28(t)* in rice (*Oryza sativa* L.). Molecular Breeding **33,** 909-918.

Yang, H., You, A., Yang, Z., Zhang, F., He, R., Zhu, L., and He, G. (2004). High-resolution genetic mapping at the Bph15 locus for brown planthopper resistance in rice (Oryza sativa L.). Theoretical and Applied Genetics **110,** 182-191.

Yang, M., Cheng, L., Yan, L., Shu, W., Wang, X., and Qiu, Y. (2019). Mapping and characterization of a quantitative trait locus resistance to the brown planthopper in the rice variety IR64. Hereditas **156,** 22.

Young-Soon, C., Hyeonso, J., Doh-Won, Y., Byoung-Ohg, A., Myung Chul, L., Seok-Cheol, S., Chun Seok, L., Eok Keun, A., Yong-Hee, J., Il-Doo, J., Jae-Keun, S., Hee-Jong, K., and Moo-Young, E. (2008). Fine Mapping of the Rice Bph1 Gene, which Confers Resistance to the Brown Planthopper (Nilaparvata lugens Stal), and Development of STS Markers for Marker-assisted Selection. Mol. Cells **26,** 146-151.

Zhang, Y., Qin, G., Ma, Q., Wei, M., Yang, X., Ma, Z., Liang, H., Liu, C., Li, Z., Liu, F., Huang, D., and Li, R. (2020). Identification of Major Locus *Bph35* Resistance to Brown Planthopper in Rice. Rice Science **27,** 237-245.

Zhao, Y., Huang, J., Wang, Z., Jing, S., Wang, Y., Ouyang, Y., Cai, B., Xin, X.-F., Liu, X., Zhang, C., Pan, Y., Ma, R., Li, Q., Jiang, W., Zeng, Y., Shangguan, X., Wang, H., Du, B., Zhu, L., Xu, X., Feng, Y.-Q., He, S.Y., Chen, R., Zhang, Q., and He, G. (2016). Allelic diversity in an NLR gene *BPH9* enables rice to combat planthopper variation. The Proceedings of the National Academy of Sciences **113,** 12850-12855.
