# Supplementary material for: Current status of molecular rice breeding for durable and broad-spectrum resistance to major diseases and insect pests
Source: Theor Appl Genet. 2024 Sep 10;137(10):219. doi: 10.1007/s00122-024-04729-3 (PMC11387466; doi:10.1007/s00122-024-04729-3)
Supplement: Supplementary file 5 — Supplementary file5 (PDF 213 KB) [file 122_2024_4729_MOESM5_ESM.pdf]

Supplemental Table 5. BPH genes/loci in rice breeding by MAS

	Released BPH-introgressed varirty	BPH breeding line	References
BPH1	IR26, IR28, IR29, IR30,IR34,IR44,IR45,IR46,IR64		(Jena and Kim, 2010)
bph2	IR32, IR36, IR38,IR40,IR42,IR48,IR50,IR52,IR54,IR65,Hwacheongbyeo		(Young-Soon et al., 2008; Jena and Kim, 2010)
BPH3	IR56, IR58, IR60,IR62, IR68,IR70,IR72, IR74,TeYou373	R373, TeYou373	(Jena and Kim, 2010; Qing et al., 2019)
bph4	IR66		(Jena and Kim, 2010)
BPH6		Luoyang-6	(Fan et al., 2017)
BPH9		Luoyang-9	(Fan et al., 2017)
BPH10		IR65482-4-136-2-2	(Ishii et al., 1994)
bph11		IR 54751-2-44-15-24-3	(Deen et al., 2010)
BPH12		B14	(Jena and Kim, 2010)
BPH13		IR54745-2-21-12-17-6	(Renganayaki et al. 2002)
BPH14		HF106	(Han et al., 2018)
BPH15		C602	(Han et al., 2018)
BPH18		IR65482-7-216-1-2	(Jena et al., 2006)
bph24(t)		IR 73678-6-9-B	(Deen et al., 2010)
bph29		RBPH54	(Wang et al., 2015)
BPH31	CR2711–76		(Prahalada et al., 2017)
BPH33(t)		RP2068	(Naik et al., 2018)
BPH35		RBPH660	(Zhang et al., 2020)
BPH36		RBPH16, RBPH17	(Li et al., 2019)
BPH37	IR64		(Yang et al, 2019)
BPH38(t)	Khazar		(Balachiranjeevi et al., 2019)
qBPH4.2		IR65482-17	(Hu et al., 2015b)
BPH6<br>*BPH9*		LuoYang69	(Wang et al., 2017)
BPH14<br>*BPH15*		B5, Luohong 4A, BR4831, BX9113-01, BX9113-12, zgkb01, zgkb02, zgkb03	(Huang et al., 2001; Zhu et al., 2013; Han et al., 2018, He et al., 2019, Chen et al., 2020)
BPH20(t)<br>*BPH21(t)*		IR71033-121-15,32-4-34, 32-4-35, 12-35-6-3, 12-35-7-16, 12-35-13-1	(Mohanapriya et al., 2019; Thulasinathan et al., 2020)
bph22(t)<br>*bph23(t)*		WB01	(Hou et al., 2011)
BPH27<br>*BPH36*		RBPH16, RBPH17	(Li et al., 2019)
bph39(t)<br>*bph40(t)*		RPBio4918-230S	(Akanksha et al., 2019)
BPH41<br>*BPH42*		SWD10	(Tan et al., 2022)
qBPH3<br>*qBPH4*		IR02W101	(Hu et al., 2015a)
BPH20(t)<br>*BPH21(t)*<br>*qBPH6(t)*		IR71033-121-15	(Jairin et al., 2007; Rahman et al., 2009)
BPH3<br>*BPH27*<br>*BPH29*<br>*BPH36*		VP1720, VP1728, VP1731, VP1732, VP1733	(Li et al., 2019)

Reference

Akanksha, S., Jhansi Lakshmi, V., Singh, A.K., Deepthi, Y., Chirutkar, P.M., Ramdeen, Balakrishnan, D., Sarla, N., Mangrauthia, S.K., and Ram, T. (2019). Genetics of novel brown planthopper *Nilaparvata lugens* (Stål) resistance genes in derived introgression lines from the interspecific cross *O. sativa* var. Swarna × O. nivara. Journal of genetics **98**.

Chen, Q., Zeng, G., Hao, M., Jiang, H., and Xiao, Y. (2020). Improvement of rice blast and brown planthopper resistance of PTGMS line C815S in two-line hybrid rice through marker-assisted selection. Molecular Breeding **40**, 21.

Deen, R., Ramesh, K., Gautam, S., Rao, Y., Lakshmi, V., Viraktamath, B., Brar, D., and Ram, T. (2010). Identification of new gene for BPH resistance introgressed from *O. rufipogon*. Rice Genetics Newsletter **25**, 70-71.

Fan, F., Li, N., Chen, Y., Liu, X., Sun, H., Wang, J., He, G., Zhu, Y., and Li, S. (2017). Development of Elite BPH-Resistant Wide-Spectrum Restorer Lines for Three and Two Line Hybrid Rice. Frontiers in Plant Science **8**.

Han, Y., Wu, C., Yang, L., Zhang, D., and Xiao, Y. (2018). Resistance to Nilaparvata lugens in rice lines introgressed with the resistance genes *Bph14* and *Bph15* and related resistance types. PLOS ONE **13**, e0198630.

He, C., Xiao, Y., Yu, J., Li, J., Meng, Q., Qing, X., and Xiao, G. (2019). Pyramiding *Xa21*, *Bph14*, and *Bph15* genes into the elite restorer line Yuehui9113 increases resistance to bacterial blight and the brown planthopper in rice. Crop Protection **115**, 31-39.

Hou, L.-y., Yu, P., Xu, Q., Yuan, X.-p., Yu, H.-y., Wang, Y.-p., Wang, C.-h., Wan, G., Tang, S.-x., Peng, S.-t., and Wei, X.-h. (2011). Genetic Analysis and Preliminary Mapping of Two Recessive Resistance Genes to Brown Planthopper, *Nilaparvata lugens* Stål in Rice. Rice Science **18**, 238-242.

Hu, J., Xiao, C., Cheng, M., Gao, G., Zhang, Q., and He, Y. (2015a). Fine mapping and pyramiding of brown planthopper resistance genes QBph3 and QBph4 in an introgression line from wild rice O. officinalis. Molecular Breeding **35**, 3.

Hu, J., Xiao, C., Cheng, M.-x., Gao, G.-j., Zhang, Q.-l., and He, Y.-q. (2015b). A new finely mapped Oryza australiensis-derived QTL in rice confers resistance to brown planthopper. Gene **561**, 132-137.

Huang, Z., He, G., Shu, L., Li, X., and Zhang, Q. (2001). Identification and mapping of two brown planthopper resistance genes in rice. Theoretical and Applied Genetics **102,** 929-934.

Ishii, T., Brar, D.S., Multani, D.S., and Khush, G.S. (1994). Molecular tagging of genes for brown planthopper resistance and earliness introgressed from *Oryza australiensis* into cultivated rice, *O. sativa*. Genome **37,** 217-221.

Jairin, J., Teangdeerith, S., Leelagud, P., Phengrat, K., Vanavichit, A., and Toojinda, T. (2007). Detection of Brown Planthopper Resistance Genes from Different Rice Mapping Populations in the Same Genomic Location. ScienceAsia **33,** 347-352.

Jena, K.K., and Kim, S.-M. (2010). Current Status of Brown Planthopper (BPH) Resistance and Genetics. Rice **3,** 161-171.

Jena, K.K., Jeung, J.U., Lee, J.H., Choi, H.C., and Brar, D.S. (2006). High-resolution mapping of a new brown planthopper (BPH) resistance gene, *Bph18(t)*, and marker-assisted selection for BPH resistance in rice (*Oryza sativa* L.). Theoretical and Applied Genetics **112,** 288-297.

Li, Z., Xue, Y., Zhou, H., Li, Y., Usman, B., Jiao, X., Wang, X., Liu, F., Qin, B., Li, R., and Qiu, Y. (2019). High-resolution mapping and breeding application of a novel brown planthopper resistance gene derived from wild rice (*Oryza. rufipogon Griff*). Rice **12,** 41.

Mohanapriya, B., Jeyaprakash, P., Raveendran, M., Soundararajan, R.P., Ramchander, S., Subashini, G., Prabhakaran, P., Manonmani, K., and Robin, S. (2019). Marker assisted introgression for brown planthopper resistance genes *Bph20* and *Bph21* in *CO43Sub1* variety of rice. Electronic Journal of Plant Breeding **10,** 645-652.

Mohanty, S.K., Panda, R.S., Mohapatra, S.L., Nanda, A., Behera, L., Jena, M., Sahu, R.K., Sahu, S.C., and Mohapatra, T. (2017). Identification of novel quantitative trait loci associated with brown planthopper resistance in the rice landrace Salkathi. Euphytica **213,** 38.

Naik, S.B., Divya, D., Sahu, N., Sundaram, R.M., Sarao, P.S., Singh, K., Lakshmi, V.J., and Bentur, J.S. (2018). A new gene *Bph33(t)* conferring resistance to brown planthopper (BPH), *Nilaparvata lugens* (Stål) in rice line RP2068-18-3-5. Euphytica **214,** 53.

Prahalada, G.D., Shivakumar, N., Lohithaswa, H.C., Sidde Gowda, D.K., Ramkumar, G., Kim, S.-R., Ramachandra, C., Hittalmani, S., Mohapatra, T., and Jena, K.K. (2017). Identification and fine mapping of a new gene, BPH31 conferring resistance to brown planthopper biotype 4 of India to improve rice, Oryza sativa L. Rice **10,** 41.

Qing, D., Dai, G., Zhou, W., Huang, S., Liang, H., Gao, L., Gao, J., Huang, J., Zhou, M., Chen, R., Chen, W., Huang, F., and Deng, G. (2019). Development of molecular marker and introgression of *Bph3* into elite rice cultivars by marker-assisted selection. Breeding science **69,** 40-46.

Rahman, M.L., Jiang, W., Chu, S.H., Qiao, Y., Ham, T.-H., Woo, M.-O., Lee, J., Khanam, M.S., Chin, J.-H., Jeung, J.-U., Brar, D.S., Jena, K.K., and Koh, H.-J. (2009). High-resolution mapping of two rice brown planthopper resistance genes, Bph20(t) and Bph21(t), originating from Oryza minuta. Theoretical and Applied Genetics **119,** 1237-1246.

Renganayaki, K., Fritz, A.K., Sadasivam, S., Pammi, S., Harrington, S.E., McCouch, S.R., Kumar, S.M., and Reddy, A.S. (2002). Mapping and Progress toward Map-Based Cloning of Brown Planthopper Biotype-4 Resistance Gene Introgressed from Oryza officinalis into Cultivated Rice, O. sativa. Crop Science **42,** 2112-2117.

Tan, H.Q., Palyam, S., Gouda, J., Kumar, P.P., and Chellian, S.K. (2022). Identification of two QTLs, BPH41 and BPH42, and their respective gene candidates for brown planthopper resistance in rice. Scientific Reports **12,** 18538.

Thulasinathan, T., Nallathambi, J., Rahman, H., Kambale, R., Ayyenar, B., Venkatasamy, B., and Muthurajan, R. (2020). Marker assisted introgression and validation of resistance genes *Bph20* and *Bph21* for brown plant hopper (*Nilaparvata lugens* sta1) into a popular rice variety of CO51. Journal of Pharmacognosy and Phytochemistry **9,** 939-944.

Wang, Y., Jiang, W., Liu, H., Zeng, Y., Du, B., Zhu, L., He, G., and Chen, R. (2017). Marker assisted pyramiding of Bph6 and Bph9 into elite restorer line 93–11 and development of functional marker for *Bph9*. Rice **10,** 51.

Young-Soon, C., Hyeonso, J., Doh-Won, Y., Byoung-Ohg, A., Myung Chul, L., Seok-Cheol, S., Chun Seok, L., Eok Keun, A., Yong-Hee, J., Il-Doo, J., Jae-Keun, S., Hee-Jong, K., and Moo-Young, E. (2008). Fine Mapping of the Rice Bph1 Gene, which Confers Resistance to the Brown Planthopper (Nilaparvata lugens Stal), and Development of STS Markers for Marker-assisted Selection. Mol. Cells **26,** 146-151.

Zhu, R., Huang, W., Hu, J., Liu, W., and Zhu, Y. (2013). Breeding of new sterile line Luohong 4A of Honglian type hybrid rice. J Wuhan Univ (Nat Sci Ed) **59,** 33-36.
