# Supplementary material for: Current status of molecular rice breeding for durable and broad-spectrum resistance to major diseases and insect pests
Source: Theor Appl Genet. 2024 Sep 10;137(10):219. doi: 10.1007/s00122-024-04729-3 (PMC11387466; doi:10.1007/s00122-024-04729-3)
Supplement: Supplementary file 6 — Supplementary file6 (PDF 291 KB) [file 122_2024_4729_MOESM6_ESM.pdf]

Supplemental Table 6. Resistance of Chinese super rice varieties to blast, BLB and BPH

Name	Resistance				References
	Blast	BLB	BPH		
Beijing1705	HR	-	-	https://ricedata.cn/variety/varis/621308.htm	
Jiahe You5	MR-MS	S	HS	https://ricedata.cn/variety/varis/620753.htm	
Jiayou Zhongke 13-1	MR	S	S	https://ricedata.cn/variety/varis/617828.htm	(Gao et al., 2019)
Long Liangyou5438	MR	S	S	https://ricedata.cn/variety/varis/620809.htm	
Zhong Zheyou H7	HS	S	HS	https://ricedata.cn/variety/varis/620869.htm	
Yuehe You1002	S	MS	HS	https://ricedata.cn/variety/varis/620757.htm	(Cai et al., 2020)
Wantai Youmeizhan	MS	HS	-	https://ricedata.cn/variety/varis/620033.htm	
Yanjing 15 (Yanjing1121)	MR	MS	-	https://ricedata.cn/variety/varis/616207.htm	(Chen et al., 2016; Zhou et al., 2018)
Suken 118 (Nanjing 13118)	MR-MS	MR-MS	-	https://ricedata.cn/variety/varis/616203.htm	(Yang et al., 2017b)
Nanjing 5718 (Ning5718)	MS	MS	-	https://ricedata.cn/variety/varis/620342.htm	(Zhang et al., 2020)
Ningjing 7 (W030)	S	S	-	https://ricedata.cn/variety/varis/615587.htm	
Nanjing 0212 (JD0212)	MS	MS	-	https://ricedata.cn/variety/varis/615318.htm	(Zhu et al., 2016)
Chugeng 37	S	R	-	https://ricedata.cn/variety/varis/615049.htm	(Huang et al., 2019; Xu et al., 2019)
Jijing 511	S	-	-	https://ricedata.cn/variety/varis/613104.htm	(Guo et al., 2014; Yang et al., 2017a)
Nanjing52 (Ning9213)	S	R	-	https://ricedata.cn/variety/varis/609134.htm	(Zhu et al., 2015)
Yabgyujing 2	S	MS	-	https://ricedata.cn/variety/varis/608805.htm	(Shao et al., 2012; Zhang et al., 2012)
Nanjing9108	S	MS	-	https://ricedata.cn/variety/varis/609133.htm	(Liu et al., 2014; Shen et al., 2014)
Zhendao 18	MS	MR	-	https://ricedata.cn/variety/varis/611540.htm	(Jing et al., 2014)
Wuyun Jing 27 (Wuyun 2743)	S	MS	-	https://ricedata.cn/variety/varis/609151.htm	(Xu et al., 2012b)
Nanjing 5055 (Ning 5055)	S	MS	-	https://ricedata.cn/variety/varis/608806.htm	(Wang et al., 2012)
Longjing 39 (Longsheng 01-030)	MR	-	-	https://ricedata.cn/variety/varis/612419.htm	(Wang, 2013)
Longjing 31 (Longhua 01-687)	R-MS	-	-	https://ricedata.cn/variety/varis/609156.htm	(Qiao, 2012)
Songjing 15 (Song 06-308)	HR-R	-	-	https://ricedata.cn/variety/varis/609972.htm	(Wu et al., 2013)
Zhendao 11 (Zhendao 413)	S	MR		https://ricedata.cn/variety/varis/604048.htm	(Jing et al., 2010)
Yangjing 4227	MR	MS	-	https://ricedata.cn/variety/varis/605695.htm	(Wang et al., 2009)
Ningjing 4 (W008)	MR	-	-	https://ricedata.cn/variety/varis/605677.htm	
Lianjing 7 (Lian 05-45)	S	MS	-	https://ricedata.cn/variety/varis/604349.htm	(Xu et al., 2010)
Longjing 21 (Longhua 99-454)	HR-R	-	-	https://ricedata.cn/variety/varis/605757.htm	(Chu et al., 2009)
Liaoxing 1 (Liaonong 21)	R	-	-	https://ricedata.cn/variety/varis/602079.htm	(Zhang et al., 2008)
Jijing 88 (Ji01-124; Ji01-22)	MS	-	-	https://ricedata.cn/variety/varis/600602.htm	(Wu and Zhao, 2005)
Liaoyou 1052	MR	-	-	https://ricedata.cn/variety/varis/600016.htm	(Hou, 2009)
Zhongzu 143	MR	HR	-	https://ricedata.cn/variety/varis/617816.htm	
Huahang No.31	R	MS	-	https://ricedata.cn/variety/varis/607506.htm	(Liu et al., 2013)
Zhongzao 39	MS	S	HS	https://ricedata.cn/variety/varis/605891.htm	(Li and Zhao, 2012)
Zhongzao 35	HS	MS	HS	https://ricedata.cn/variety/varis/605894.htm	(Luo et al., 2011)
Jinnong Simiao	MS	MR	-	https://ricedata.cn/variety/varis/607502.htm	
Zhong Jiazao 17	HS	S	HS	https://ricedata.cn/variety/varis/605661.htm	(Luo et al., 2009)
He Meizhan	MS	MR	-	https://ricedata.cn/variety/varis/604183.htm	
Yuxiang Youzhan	HR	MR	-	https://ricedata.cn/variety/varis/600880.htm	(Zheng and Bai, 2005)
Gui Nongzhan	S	S	-	https://ricedata.cn/variety/varis/600873.htm	(Zheng et al., 2005)
Y Liangyou 305	HR	HS	-	https://ricedata.cn/variety/varis/615775.htm	(Peng et al., 2018)
Jiang Liangyou 1988	MR	S	HS	https://ricedata.cn/variety/varis/618463.htm	
Shen Liangyou 862	S	MS	S	https://ricedata.cn/variety/varis/615474.htm	
Long Liangyou 1308	MR	S	HS	https://ricedata.cn/variety/varis/616737.htm	(Liu et al., 2021)
Long Liangyou 1377	MS	MS	HS	https://ricedata.cn/variety/varis/616688.htm	
He Liangyou 713	MR	MS	HS	https://ricedata.cn/variety/varis/616597.htm	
Y Liangyou 957	HS	MS	HS	https://ricedata.cn/variety/varis/616623.htm	
Long Liangyou 1212	MS	MS	HS	https://ricedata.cn/variety/varis/615692.htm	
Jing Liangyou 1212	MR	S	HS	https://ricedata.cn/variety/varis/615951.htm	
Long Liangyou 1988	S	HS	HS	https://ricedata.cn/variety/varis/615945.htm	
Shen Liangyou 136	HS	MS	HS	https://ricedata.cn/variety/varis/615940.htm	
Long Liangyou 1988	S	MS	MS	https://ricedata.cn/variety/varis/615945.htm	
Shen Liangyou 136	HS	MS	HS	https://ricedata.cn/variety/varis/615940.htm	
Y Liangyou 900	HS	HS	HS	https://ricedata.cn/variety/varis/614537.htm	(Li et al., 2013)
Long Liangyou	MR	S	HS	https://ricedata.cn/variety/varis/615342.htm	(Liu et al., 2016)
Shen Liangyou 8386	S	MS	-	https://ricedata.cn/variety/varis/615252.htm	
Y Liangyou 1173	R	S	-	https://ricedata.cn/variety/varis/615190.htm	(Liu et al., 2018a)
Hui Liangyou 996	HS	S	HS	https://ricedata.cn/variety/varis/613040.htm	
Shen Liangyou 870	R-MS	MS-S	-	https://ricedata.cn/variety/varis/614817.htm	
H Liangyou 991	MS-S	MS-HS	-	https://ricedata.cn/variety/varis/610937.htm	
Liangyou 616	MS	-	-	https://ricedata.cn/variety/varis/612252.htm	(Wang and Wang, 2012)
Liangyou 6	HS	S	HS	https://ricedata.cn/variety/varis/612063.htm	(Shu et al., 2013)
Guang Liangyou 272	HS	MS	-	https://ricedata.cn/variety/varis/612404.htm	(Liu et al., 2012)
C Liangyou Huazhan	S	S	HS	https://ricedata.cn/variety/varis/609267.htm	(Lin et al., 2014)
Liangyou 038	HS	-	-	https://ricedata.cn/variety/varis/607363.htm	(Xiao et al., 2012)
Y Liangyou 5867 (Shen Liangyou 5867)	MS	MR	HS	https://ricedata.cn/variety/varis/607361.htm	(Rao, 2011)
Y Liangyou 2	HS	S	HS	https://ricedata.cn/variety/varis/609898.htm	(Zhang, 2010)
Y Liangyou 087	S	S	-	https://ricedata.cn/variety/varis/607485.htm	Li et al., 2011)
Zhun Liangyou 608	HS	HS	HS	https://ricedata.cn/variety/varis/605672.htm	(Zhou et al., 2009)
Shen Liangyou 5814	S	MS	HS	https://ricedata.cn/variety/varis/604198.htm	(Tong et al., 2008)

Guangliangyouxiang	S	MS	S	https://ricedata.cn/variety/varis/605726.htm	(You et al., 2009)
Lingliangyou 268	S	S	MR	https://ricedata.cn/variety/varis/605259.htm	(Huang et al., 2011)
YangLiangyou 6	S	MR	MS	https://ricedata.cn/variety/varis/600543.htm	(Dai et al., 2005)
Feng Liang You Xiang No.1	HS	S	-	https://ricedata.cn/variety/varis/600652.htm	(Xu et al., 2008)
Fengliangyou 4 (Wandao 187)	HS	S	HS	https://ricedata.cn/variety/varis/601304.htm	(Zhou et al., 2007)
Zhuliangyou 819	S	S	-	https://ricedata.cn/variety/varis/600951.htm	(Shan et al., 2006)
Liangyou 287	HS	S	-	https://ricedata.cn/variety/varis/600958.htm	(Zhou et al., 2008)
Xinliangyou 6	HS	S	-	https://ricedata.cn/variety/varis/601039.htm	(Zhang et al., 2007)
Quanyou 212	MR	-	-	https://ricedata.cn/variety/varis/617687.htm	
Jiafengyou 2	S	MS	HS	https://ricedata.cn/variety/varis/616447.htm	(Wang et al., 2018)
Huazheyou 71	S	MR-S	HS	https://ricedata.cn/variety/varis/616429.htm	
Funongyou 676	MS	-	-	https://ricedata.cn/variety/varis/617686.htm	
Jiyouhang 1573	HS	-	-	https://ricedata.cn/variety/varis/615362.htm	(Li et al., 2016)
Taiyou 871	MS	-	-	https://ricedata.cn/variety/varis/615906.htm	
Longfengyou 826	S	HS	-	https://ricedata.cn/variety/varis/616845.htm	(Chen et al., 2019)
Jingyou Huazhen	MS	MS-S	-	https://ricedata.cn/variety/varis/616599.htm	
Hua Zhenyou 1	HS	HS	HS	https://ricedata.cn/variety/varis/616430.htm	
Wantaiyou 3158 (Wanfengyou 3158; Dengyou 3158)	MS	MS-HS	-	https://ricedata.cn/variety/varis/618012.htm	(Zhou et al., 2019)
Wuyou 369	MS	MS-S	-	https://ricedata.cn/variety/varis/614857.htm	(Tang et al., 2016)
Neuxiang 6 you 9	S	-	HS	https://ricedata.cn/variety/varis/615457.htm	(Yuan et al., 2016)
Shuyou 217	MS	HS	HS	https://ricedata.cn/variety/varis/615463.htm	(Liao et al., 2017)
Huyou 727	S	MS-S	HS	https://ricedata.cn/variety/varis/615545.htm	
Jiyou 615	R	HS	-	https://ricedata.cn/variety/varis/615152.htm	
Wuyou 1179	MS	HS	-	https://ricedata.cn/variety/varis/615187.htm	(Liu et al., 2018b)
Yixiang 4245	S	S	HS	https://ricedata.cn/variety/varis/605705.htm	(Wang et al., 2010)
Jifengyou 1002	HR	S	-	https://ricedata.cn/variety/varis/614320.htm	
Wuyou 116 (Wufengyou 116)	R	HS	-	https://ricedata.cn/variety/varis/615160.htm	
Deyou 4727 (Deyou 427)	S	MS	S	https://ricedata.cn/variety/varis/614121.htm	(Guo et al., 2016)
Fengtianyou 553	R-S	S-HS	-	https://ricedata.cn/variety/varis/614179.htm	(Pang et al., 2013)
Wuyou 662 (Wufengyou 662)	HS	-	-	https://ricedata.cn/variety/varis/612267.htm	(Xu et al., 2012a)
Jiyou 225	HS	-	-	https://ricedata.cn/variety/varis/615003.htm	
Wufengyou 286	HS	S	HS	https://ricedata.cn/variety/varis/612437.htm	
Wuyou 1573 (Wuyouhang 1573)	HS	-	-	https://ricedata.cn/variety/varis/615010.htm	(Mao et al., 2014)
Yixiangyou 2115	R	-	HS	https://ricedata.cn/variety/varis/611178.htm	
Shenyou 1029	HS	HS	HS	https://ricedata.cn/variety/varis/614262.htm	
F you 498	S	-	S	https://ricedata.cn/variety/varis/605602.htm	
Rongyou 225	HS	MS	HS	https://ricedata.cn/variety/varis/605450.htm	(Yan et al., 2009)
Nei 5 You 8015 (Guodao 7)	HS	HS	HS	https://ricedata.cn/variety/varis/607291.htm	(Yan et al., 2011)
Shengtaiyou 722	MS	-	-	https://ricedata.cn/variety/varis/612925.htm	(Lan et al., 2012)
Wufengyou 615	MR	S	-	https://ricedata.cn/variety/varis/612189.htm	(Liu et al., 2019)
Tianyou 3618	R	MS	-	https://ricedata.cn/variety/varis/605471.htm	(Zhu et al., 2012)
Tianyou Huazhan	MS	S	S	https://ricedata.cn/variety/varis/605274.htm	(Yu et al., 2009)
Zhong 9 you 8012	HS	S	HS	https://ricedata.cn/variety/varis/605665.htm	
H you 518	HS	S	HS	https://ricedata.cn/variety/varis/607432.htm	(Tang et al., 2012)
Dexiang 4103	HS	HS	S	https://ricedata.cn/variety/varis/605046.htm	(Zheng et al., 2008)
Yiyou 673 (Yixiangyou 673)	HS	HS	S	https://ricedata.cn/variety/varis/605370.htm	(Huang et al., 2006)
Shenyou 9516	R	MS	-	https://ricedata.cn/variety/varis/607527.htm	(Zhou, 2011)
Teyou 582	S	S	-	https://ricedata.cn/variety/varis/605565.htm	(Dai et al., 2015)
Wuyou 308	HS	S	MS	https://ricedata.cn/variety/varis/605267.htm	(Huang et al., 2010)
Tianyou 3301	MS	HS	S	https://ricedata.cn/variety/varis/604136.htm	(Wen and Chen, 2009)
Luoyou 8 (Honglianyou 8)	HS	S	-	https://ricedata.cn/variety/varis/602148.htm	(Zhu et al., 2008)
Zhongzheyou 1	MR-S	MS-MR	-	https://ricedata.cn/variety/varis/600617.htm	(Zhang et al., 2005)
II youming 86	MS	S	S	https://ricedata.cn/variety/varis/600653.htm	(Liang, 2003)
II you 602 (Beifeng 3)	HS	S	MS	https://ricedata.cn/variety/varis/600072.htm	(Liu et al., 2004)
Tianyou 998 (Tianfengyou 998)	HS	S	-	https://ricedata.cn/variety/varis/600017.htm	(Liang et al., 2004)
Yongyou 7850	MR	MS	HS	https://ricedata.cn/variety/varis/615312.htm	
Yongyou 1540	HS	MS	HS	https://ricedata.cn/variety/varis/614572.htm	(Cai et al., 2018)
Yongyou 2640	MR-MS	MR-MS	-	https://ricedata.cn/variety/varis/613565.htm	
Yongyou 538	MR	MS	S	https://ricedata.cn/variety/varis/613563.htm	
Chunyou 84 (Chunyou 684)	MR	S	S	https://ricedata.cn/variety/varis/613561.htm	(Wu et al., 2014)
Zheyou 18 (Zheyou 818)	MS	MS	S	https://ricedata.cn/variety/varis/612346.htm	
Yongyou 15 (06G370)	R-MS	S	S	https://ricedata.cn/variety/varis/612348.htm	(Sun et al., 2013)
Yongyou 12 (05-G364)	MR	MS	S	https://ricedata.cn/variety/varis/605000.htm	(Sun et al., 2014)

The super rice varieties have been admitted by the Ministry of Agriculture and Rural Affairs of China by 2020. HR: highly resistant; R: resistant; MR: moderate resistant; MS: moderate susceptible; S: susceptible; HS: highly susceptible: - indicates no data. BLB: bacterial leaf blight disease; BPH: brown planthopper. Data were collected from China Rice Data Center (https://ricedata.cn)

References

Cai, K., Ma, R., Wang, X., Lu, Y., Zhou, H., Tang, Z., Wang, Y., and Chen, H. (2018). Breeding and Application of Wide Adaptability Hybrid Indica-japonica Rice Yongyou 1540. China Rice **24,** 118-119.

Cai, L., Yang, W., Liang, T., Li, J., Zhuang, J., and Feng, d. (2020). Breeding for a new hybrid rice combination Yuehe You1002 of the high yield and quanlity and high-yield cultivation techniques. Bulletin of Agricultural Science and Technology**,** 261-263.

Chen, W., Deng, G., Dai, G., Zhou, W., Zhou, M., Liang, H., Liang, S., Chen, R., and Chen, R. (2019). Breeding and application of Longfengyou 826 with weak photosensitivity, good quality and high yiel in hybrid rice. Guizhou Agricultural Sciences **47,** 1-4.

Chen, Y., Zhang, Y., Si, Z., Dayou, Z., Shao, F., and Liang, S. (2016). Breeding for a new rice variety, Yanjing 15 and high-yield cultivation techniques. Bulletin of Agricultural Science and Technology, 189-190.

Chu, G., Zhang, S., Wang, R., and Wang, C. (2009). Characteristics and high yiled cultivation techniques of the super rice Longjing 21. North Rice **39,** 56-57.

Dai, G., Deng, G., Chen, R., Liang, S., Zhou, M., Zhou, W., and Gao, L. (2015). Breeding and Application of Teyou 582, a Middle-maturity Early Indica Super Rice Cultivar. Guizhou Agricultural Sciences **43,** 5-6,23.

Dai, Z., Liu, G., Li, A., Xu, M., Liu, X., Zhou, C., and Hongxi, Z. (2005). Breeding of Two-line Indica Hybrid Rice Combination, "Yangliangyou 6", and Studying on its Culture Characteristics. Chinese Agricultural Science Bulletin **21,** 114-116.

Gao, R., Fu, X., Lu, J., and Li, J. (2019). Jiayou Zhongke 13-1, a New Three-line Hybrid Rice Combination with Fine Grain Quality. HYBRID RICE **34,** 93-94.

Guo, Gui-zhen, Zhang, K.-l., Yang, C.-g., and Qiu, Z.-g. (2014). Breeding and High Yield Cultivation Techniques of a New Rice Variety Jijing 511. China Rice **20**.

Guo, X.-j., Zhang, T., Jiang, K.-f., Yang, L., Qin, J., Cao, Y.-j., You, S.-m., Yang, Q.-h., Wan, X.-q., Luo, J., Li, Z.-x., Gao, L., and Zheng, J.-k. (2016). Breeding and Application of New Hybrid rice Combination Deyou 4727 with High Yield and Fine Quality. Hybrid Rice **31,** 16-18.

Hou, Z. (2009). Introduction, and green and high yield cultivation techniques of Liaoyou 1052. North Rice **39,** 55-56.

Huang, G., Yu, X., and Zhang, X. (2011). Performance and High-yielding Cultural Techniques of Two-line Early Hybrid Rice Lingliangyou 268 in Demonstration at Liling, Hunan. Hybrid Rice **26,** 40-41.

Huang, H., Wang, F., Wu, Y., Fu, F., Zhu, M., Huang, D., Liu, Z., Liao, Y., Liu, W., and Li, J. (2010). Wuyou 308, a New Super Hybrid Rice Combination with Good Grain Quality. Hybrid Rice **25,** 82-84.

Huang, T., Xie, H., You, Q., Wang, W., Zheng, J., Zhang, H., and Huang, H. (2006). Breeding and Application of "Yiyou 673" - A new combination of fragrant rice with high quality and yield. Acta Agriculturae Jiangxi **18,** 6-9.

Huang, W., LI, K., Ruan, W., Zhang, T., Xu, J., Wang, Z., and Duan, H. (2019). Breeding and Application of A new Super Rice Variety Chugeng 37. China Rice **25,** 115-116.

Jing, D., Diao, L., Lin, T., Hu, C., Gong, H., Zhou, Y., Qian, H., Li, C., Yu, B., and Sheng, S. (2010). Breeding and high yield cultivation techniques of an early-maturing late japonica Zhendao 11. Jiangsu Agricultural Sciences, 94-95.

Jing, D., Qian, H., Lin, T., Yu, B., Gong, H., Zhou, Y., Li, C., Zeng, S., Zhang, X., Yang, J., and Sheng, S. (2014). Breeding of a new early-maturing late japonica rice cultivar Zhendao 18 and high and stable yield analysis. Jiangsu Agricultural Sciences **42,** 88-89, 164.

Lan, S., Zhang, S., Shi, L., Yu, M., Yang, X., and Liu, Z. (2012). High-yielding Autumn Seed Production Techniques of New Late Indica Hybrid Rice Combination Shengtaiyou 722 by Broadcasting Seedlings of Female Parent. Hybrid Rice **27,** 33-35.

Li, H., and Zhao, Y. (2012). Planting nad high yield cultivation techniques of Zhongzao 38 in Dongyang City. China Rice **18,** 54-56.

Li, J., Deng, Q., Zhang, Y., Xiao, L., and Wu, J. (2013). Cultural Techniques of Y Liangyou 900, a Promising Hybrid for Phase IV Super Rice, Yielding 14.82 t/hm2 in High-yielding Pilot Demonstration. Hybrid Rice **28,** 46-48.

Li, Y.-q., Zou, Y.-h., Li, W.-r., Liu, Y.-h., Chun-yu, H., Wei, Q.-y., Liu, W.-d., Zhou, Y.-m., and Tan, J.-l. (2011). Breeding and Application of New Two-line Hybrid Rice Combination Y Liangyou 087. Hybrid Rice **26,** 14-17.

Li, Y., Nie, Y., Huang, Q., Cao, Z., Cai, Y., and Yang, P. (2016). Jiyouhang 1573, a New Late Indica Hybrid Rice Combination. Hybrid Rice **31,** 88-90.

Liang, G. (2003). High-yielding Hybrid Seed Production Techniques of Ⅱ You Ming 86. Hybrid Rice **18,** 32-33.

Liang, S., Li, C., Fu, F., Huang, H., and Zhu, M. (2004). Tianyou 998, a New Indica Hybrid Rice Combination with High Yield and Good Quality. Hybrid Rice **19,** 62-63.

Liao, Y.-x., Xu, P.-z., Zheng, J.-g., Luo, Q.-x., Gao, K.-f., Yang, H.-s., Tian, Y.-f., Xiang, G.-r., Zhang, S.-q., Jiang, J.-l., Zhang, H.-y., Chen, X.-q., and Wu, X.-j. (2017). Breeding and Application of Indica CMS Line Shu 21A in Rice. Hybrid Rice **32,** 14-15,19.

Lin, Y., Yang, B., Zeng, Q., and Du, J. (2014). C Liangyou Huazhan, a New Two-line Medium Hybrid Rice Combination with High Yield and Good Quality. Hybrid Rice **29,** 76-77.

Liu, A., Sun, Y., Zheng, J., and Xiong, K. (2014). Characteristics and cultivation techniques of the rice new variety Nanjing 9108. Modern Agricultural Science and Technology.

Liu, G., Kuang, H., and Wen, S. (2004). Ⅱ You 602, a New Indica Hybrid Rice Combination with High Yield and Wide Adaptability. Hybrid Rice **19,** 63-64.

Liu, H., Chen, C., and Song, J. (2016). Performance and High-yielding Cultivation Techniques of Longliangyou Huazhan in Huichang， Jiangxi. Hybrid Rice **31,** 36-37.

Liu, K., Yang, G.-c., Chen, Z.-j., Hu, G., Zhou, L., Li, S.-h., and You, A.-q. (2012). Breeding and Application of New Two-line Medium Hybrid Rice Combination Guangliangyou 272 with Fine Quality. Hybrid Rice **27,** 31-33.

Liu, S., Fu, C., Qin, P., Li, C., Hu, X., Fu, X., Wu, T., and Yang, Y. (2021). Breeding of the green super hybrid rice Long Liangyou 1308 with high yield and quality, and wide adaptability. Bulletin of Agricultural Science and Technology, 279-283.

Liu, y.-z., Wang, H., Guo, T., Zhang, J.-g., Tang, X.-r., and Chen, Z.-q. (2013). Breeding and application of high-quality and disease-resistant rice variety, huahang No.31. Guangdong Agricultural Sciences **40,** 8-11.

Liu, Y.-z., Xiao, W.-m., Wang, H., Guo, T., Tang, X.-r., and Chen, Z.-q. (2018a). Breeding and Application of New Two-line Super Hybrid Rice Combination Y Liangyou 1173. Hybrid Rice **33,** 17-19, 24.

Liu, Y., Xiao, W., Wang, H., Guo, T., and Chen, Z. (2018b). Breeding and High – yield Culture Technology of a New Super Hybrid Rice Combination Wuyou 1179. Crop Research **32,** 280-282.

Liu, Z.-x., He, Z.-y., Lin, Q.-s., Feng, D.-j., Gao, Y., Hu, W., Zhang, Q., Lliang, Q.-d., and Jiang, Y.-j. (2019). Breeding and Application of Super Hybrid Rice Combination Wufengyou 615. Hybrid Rice **34,** 12-14.

Luo, J., Yao, R., Tang, S., Jiao, G., Shao, G., and Hu, P. (2009). Breeding and cultivation techniques of the super high yiled early rice variety Zhong Jiazao 17. China Rice, 50-51.

Luo, L., Chen, C., Song, F., Yu, J., and Yan, J. (2011). Characteristics and cultivation techniques od the new early indica Zhongzao 35. China Rice **17,** 70-73.

Mao, L., Li, Y., Nie, Y., Xie, H., Cao, Z., Yan, M., Huang, Y., Fan, Z., Cai, Y., Yan, L., and Mao, Y. (2014). Wuyouhang 1573, a New late hybrid rice combination. Hybrid Rice **29,** 86-88.

Pang, G., Liang, M., Yan, Y., Chen, C., and Su, X. (2013). Fengtianyou 553, a New Late Hybrid Rice Combination. Hybrid Rice **28,** 90,94.

Peng, R., Peng, J., Tang, Z., Sun, J., and Mao, Z. (2018). Y Liangyou 305, a New Hybrid Rice Combination with High Blast Resistance. Hybrid Rice **33,** 76-77.

Qiao, X. (2012). The new super rice variety Longjing 31, an early japonica with high quality in cold region China Agricultural Technology Extension **28,** 17-18.

Rao, X. (2011). Breeding and Application of Good-quality and High-yielding Hybrid Rice New Combination "Y-Liangyou 5867". Acta Agriculturae Jiangxi **23,** 4.

Shan, P., Fu, C., and Xie, J. (2006). Characteristics and Cultural Techniques of the NewHybrid Rice Combination Zhuliangyou 819. Hybrid Rice **21,** 50-51.

Shao, F., Zhang, D., Xu, D., Dong, J., Zhang, Y., Xu, J., Liu, J., Chen, Y., and Li, Y. (2012). Breeding and cultivation techniques of rice new variety Yangyujing 2. Bulletin of Agricultural Science and Technology, 88-89.

Shen, B., Ding, X., and Sun, R. (2014). Demonstration experiment of the rice new variety Nanjing 9108. Modern Agricultural Science and Technology, 44-45.

**Shu, B., Tu, Z., Duan, H., Liu, Q., Xie, X., Zhang, S., and Yang, Q.** (2013). Liangyou 6, a New Two-line Early-cropping Indica Hybrid Rice Combination with High Yield and Fine Grain Quality. Hybrid Rice **28,** 85-87.
**Sun, J., Yan, B., Wu, M., Dai, J., and Zhao, S.** (2013). High-yielding Cultivation Techniques of New Hybrid Rice Combination Yongyou 15. Hybrid Rice **28,** 45-46.
**Sun, Y.-f., Liang, Y.-m., Chen, Y.-g., Feng, Z.-m., Chen, J.-h., and Chen, F.** (2014). Preliminary Integration of Hand-transplanted Cultivation Techniques for 1000 kg/667 m^2 of Super Rice Yongyou 12. China Rice **20,** 63-67.
**Tang, S., Deng, Y., and Xu, K.** (2016). High yield late indica Wuyou 369 and high yield cultivation technique. China Seed Industry**,** 80-81.
**Tang, W., Chen, G., Xiao, Y., Deng, H., and Chen, L.** (2012). Seed Production Techniques of New Three-line Late Hybrid Rice Combination H You 518. Hybrid Rice **27,** 33-34.
**Tong, X., Ceng, S., Chu, F., Ma, Y., Qian, X., Zhang, C., Jin, W., and Tang, J.** (2008). High-yielding Cultural Techniques for Shenliangyou 5814, a Two-line Hybrid Rice Combination with Good Grain Quality and High Yield. Hybrid Rice **23,** 48-49.
**Wang, B., Dai, Z., Zhao, B., Li, A., Zhou, C., Liu, X., Li, Y., and Zhang, H.** (2009). Characteristics and cultivation techniques of a early-maturing late japonica Yangjing 4227. Jiangsu Agricultural Sciences**,** 117-118.
**Wang, C., Zhang, Y., Zhu, Z., Chen, T., Zhao, Q., Zhao, L., Zhou, L., and Yao, S.** (2012). Breeding and application of a new japonica Nanjing 5055 with good eating quality. Bulletin of Agricultural Science and Technology**,** 84-88.
**Wang, C., Zhang, Y., Zhu, Z., Yao, S., Zhao, Q., Chen, T., Zhou, L., and Zhao, J.** (2013). Breeding and application of a new japonica Nanjing 9108 with good eating quality. Jiangsu Agricultural Sciences **41,** 86-88.
**Wang, F., Lin, G., Zhao, D., Jiang, Q., Li, Y., He, B., and Yang, C.** (2010). Yixiang 4245,a New Quasi-aromatic Hybrid Rice Combination with Good Grain Quality. Hybrid Rice **25,** 85-86.
**Wang, R.** (2013). Breeding of a new rice variety Longjing 39 with high yield and quality. Heilongjiang Agricultural Sciences**,** 167-168.
**Wang, S.-l., Ding, Z.-q., and Huang, H.-x.** (2018). Breeding and Application of New Indica-japonica Hybrid Rice Combination Jiafengyou 2. Hybrid Rice **33,** 24-26.
**Wang, Z.-f., and Wang, W.-q.** (2012). Breeding and application of two-line hybrid rice "Liangyou616". Fujian Agricultural Science and Technology**,** 1-4.
**Wen, Y., and Chen, M.** (2009). Performance and High-yielding Cultural Techniques of Tianyou 3301 Grown as Late Rice after Tabacco. Hybrid Rice **24,** 57,90.
**Wu, H., Yan, P., Mou, F., Yu, Y., and Zhang, S.** (2013). Breeding and cultivation techniques of a new japonica Songjing 15 with super high yield. Crops **3,** 148.
**Wu, L., and Zhao, J.** (2005). Characteristics and cultivation techniques of the high yield and quality rice variety Jijing 88. Xinjiang Agricultural Science and Technology**,** 10.
**Wu, M.-g., Lin, J.-r., Song, X.-w., and Ruan, G.-h.** (2014). reeding of New Japonica-indica Hybrid Rice Combination Chunyou 84. Hybrid Rice **29,** 19-21.
**Xiao, C., Yu, Q., Zhang, S., Chen, D., Zhang, Y., and Qin, Y.** (2012). Liangyou 038, a New High-yielding Two-line Hybrid Rice Combination. Hybrid Rice **27,** 88-90.
**Xu, J., Zhou, G., and Zhang, G.** (2008). Breeding and Application of New Rice Variety Feng Liang You Xiang No.1 with High Quality and Yield. Journal of Anhui Agricultural Sciences **36,** 4016-4017.
**Xu, J., Li, K., and Ruan, W.** (2019). Breeding and application of suer rice variety in Yunnan Plateau. Bulletin of Agricultural Science and Technology **2,** 215-217.
**Xu, J., He, C., Lei, Y., Yan, Z., and Hu, G.** (2012a). Wufengyou 662, a New Late Hybid Rice Combination. Hybrid Rice **27,** 91.
**Xu, X., Zhu, B., Shi, S., Xu, J., Zhang, Q., and Xu, Y.** (2012b). Breeding and cultivation techniques of a new japonica Wuyun jing 27 with high quality. Jiangsu Agricultural Sciences **40,** 102-103.
**Xu, Z., Wang, H., Zhang, L., Su, X., Li, D., Yu, S., and Zhong, W.** (2010). The discussion on the high yield cultivation techniques of a rice new variety Lianjing 7. Jiangsu Agricultural Sciences**,** 106-107.
**Yan, C., Cao, L., Ruan, G., Huang, Y., Chen, S., and Hong, X.** (2011). Effects of Nitrogen Rates on Dry Matter Production after Heading,Yield and Yield Structure of Nei 5 You 8015. Hybrid Rice **26,** 43-46.
**Yan, M., Li, Y., Cai, Y., Li, M., Mao, L., Li, Y., and Yan, L.** (2009). Characteristics and cultivation techniques of a new late-cropping hybrid rice Rongyou 225. China Rice**,** 71-72.
**Yang, C., Guizhen, G., Zhou, G., and Qiu, Z.** (2017a). Breeding and Popularization of Good Eating Quality New Super Rice Variety 'Jijing 511'. Journal of Northeast Agricultural Sciences **42,** 4-7.
**Yang, J., Zhong, W., Wang, J., Zhu, J., Li, W., and Wang, F.** (2017b). The breeding and application of a new japonica Suken 118 with good eating quality. Jiangsu Agricultural Sciences **45,** 79-81.
**You, Q.-t., Wang, Z., and He, X.-j.** (2009). Performance and Cultivation Techniques of Two-line Hybrid Rice Variety Guangliangyouxiang 66 Planted in Southern Henan. Hubei Agricultural Sciences **48,** 2367-2368.
**Yu, S., Zhu, L., Ouyang, Y., Xu, D., Zhu, X., and Jin, Q.** (2009). Characteristics and High-yielding Cultivation Techniques of New Hybrid Rice Combination Tianyou Huazhan. Hybrid Rice **24,** 42-44.
**Yuan, X., Li, Y., Liu, J., Fu, W., Liu, X., and Fu, Q.** (2016). Breeding and Utilization of High Yield and Blast-resistant Hybrid Rice Neixiang 6 you 9. Hybrid rice **22,** 86-87.
**Zhang, C., Chen, J., Jiang, J., Wang, H., Yan, Z., Jiang, J., Chen, L., Liu, X., Zhang, Y., and Jiang, Z.** (2007). Xinliangyou 6, a NewTwo-line Super Hybrid Rice Combination. Hybrid Rice **22,** 76-78.
**Zhang, D., Wang, Y., Xu, D., Dong, J., Shao, F., Zhang, Y., Xu, J., Liu, J., Chen, Y., Dong, X., and Li, Y.** (2012). Characteristics and cultication techniques of rice new variety Yangyujing 2. Jiangsu Agricultural Sciences **40,** 109-110.
**Zhang, M.** (2010). Planting and high yield cultivation techniques of the dominant hybrid rice combination Y Liangyou 2 with high quality. Bulletin of Agricultural Science and Technology**,** 142-143.
**Zhang, S.q., Tong, H.h., Cao, Y.p., Tong, H.j., Yin, S.f., and Tang, C.h.** (2005). Breeding and Application of New Indica Hybrid Rice Combination Zhongzheyou 1 with High Yield and Good Quality. Hybrid Rice **20,** 21-22.
**Zhang, Y.-z., Wang, C.-h., Zheng, W., Zhao, J., Teng, G., and Zhou, Y.-h.** (2008). Analysis on Utilization of Luminous Efficiency in a New Rice Variety 'Liaoxing 1'. Liaoning Agricultural Sciences**,** 11-13.
**Zhang, Y., Zhu, Z., Tao, C., Qingyong, Z., Feng, K., Yao, S., Lihui, Z., Ling, Z., Zhao, C., Liang, W., Lu, K., and Wang, C.** (2020). Breeding and Characteristics of a New Japonica Rice Variety Nanggeng 5718 with Good Eating Quality. China Rice **26,** 100-102.
**Zheng, C., and Bai, S.** (2005). The high yield cultivation technique makes the per unit area yield of super rice Yuxiang Youzhan reach 756 kg. Guangdong Agricultural Sciences**,** 25-26.
**Zheng, C., Lu, Y., and Gao, Y.** (2005). Introduction and cultivation techniques of the super rice Gui Nongzhan with wide adaptability. Guangdong Agricultural Sciences**,** 22-23.
**Zheng, J.-k., Jiang, K.-f., Yang, Q.-h., Zhang, T., Yang, L., Wan, X.-q., Cao, Y.-j., and You, S.-m.** (2008). Breeding of New Hybrid Rice Combination Dexiang 4103 and Analysis of the Reasons for Its High Yield. Hybrid Rice **23,** 13-15.
**Zhou, G., Zhang, G., and Xu, X.** (2007). Fengliangyou 4, a New Medium Indica Hybrid Rice Combination. Hybrid Rice **22,** 91-92.
**Zhou, J.-y., Liao, C.-m., Liu, J.-b., and Song, C.-f.** (2009). High-yielding Cultural Techniques of Two-line Hybrid Rice Zhunliangyou 608 Demonstrating in Sanya. Hunan Agricultural Sciences, 33-34, 42.
**Zhou, J., Zhang, Y., Zhang, Q., Chen, Y., Si, Z., Liang, S., and Lu, L.** (2018). The breeding and application of a new rice variety Yanjing 15. Bulletin of Agricultural Science and Technology**,** 161-163.

Zhou, W., Chen, W., Dai, G., Liang, H., Zhou, M., Chen, R., and Deng, G. (2019). Wantaiyou 3158, a New Super Hybrid Rice Combination for Both Early and Late Season. Hybrid Rice **34,** 80-82.

Zhou, Y. (2011). Performance and High-yielding Cultural Techniques of Shenyou 9516 Planted at Xinyi, Guangdong. Hybrid Rice **26,** 52-53.

Zhou, Y., Ju, C., Xu, G., Xie, P., and Gao, M. (2008). Liangyou 287,a New Two-line Early-cropping Super Hybrid Rice Combination with Fine Grain Quality. Hybrid Rice **23,** 71-72.

Zhu, M., Fu, F., Wang, F., Huang, H., Huang, D., Liao, Y., Liu, W., Liu, Z., Li, J., and Chen, J. (2012). Tianyou 3618, a New Indica Hybrid Rice Combination with High Yield and Blast Resistance. Hybrid Rice **27,** 89-90.

Zhu, R., Wu, D., Cai, M., and Zhu, Y. (2008). High-yielding and Simplified Cultivation Techniques for Quality Hybrid Rice Luoyou 8. Hybrid Rice **23,** 39-41.

Zhu, Z., Zhao, L., Zhao, Q., Zhang, Y., Chen, T., Yao, S., Zhou, L., Yu, X., and Wang, C. (2015). Breeding and application of a high yield and quality rice variety Nanjing 52 with resistance to stripe disease. Jiangsu Agricultural Sciences **43,** 77-79.

Zhu, Z., Zhao, Q., Chen, T., Yao, S., Zhou, L., Zhang, Y., Zhao, L., Yu, X., and Wang, C. (2016). The breeding and application of a new japonica Nanjing 0212 with high yield and quality. Jiangsu Agricultural Sciences **44,** 127-129.
