# Supplementary material for: Current status of molecular rice breeding for durable and broad-spectrum resistance to major diseases and insect pests
Source: Theor Appl Genet. 2024 Sep 10;137(10):219. doi: 10.1007/s00122-024-04729-3 (PMC11387466; doi:10.1007/s00122-024-04729-3)
Supplement: Supplementary file 7 — Supplementary file7 (PDF 147 KB) [file 122_2024_4729_MOESM7_ESM.pdf]

Supplemental Table 7. Resistance of Indian high-yielding rice varieties to blast, BLB and BPH

	Resistance		
	Blast	BLB	BPH
Padma	MR	-	-
Bala	MR	-	-
Kiron	-	-	-
Krishna	R	-	-
Ratna	MR	-	-
Vijaya	R	-	-
Saket-4	-	MR	-
Jayanti	-	-	-
Kalinga-I	S	S	S
Kalinga-II	S	S	S
Shakti	-	-	-
Supriya	-	MR	-
Vani	MR	MR	-
Naikichili	-	-	-
Anamika	MR	MR	-
Indira	MR	MR	-
Pallavi	-	R	-
Ramakrishna	-	R	-
Samalei	R	-	-
Sattari	R	R	R
Narendra-1	-	-	-
Savitri/ Ponmani	MR	-	-
Khitish	-	-	-
CR 138-928	-	MR	-
Kalinga-III	-	-	-
Utkalprabha	-	-	-
Neela	-	-	MR
Sarasa	-	-	MR
Udaya	R	-	-
Annada	MR	-	-
CR 1014	-	-	-
Dharitri	MR	MR	-
Gayatri	MR	MR	-
Heera	-	-	-
Kalashree	MR	-	-
Kalyani-II	R	-	-
Kshira	R	-	R
Moti	R	-	-
Padmini	-	R	-
Panidhan	-	-	-
Tara	R	-	R
Tulasi	-	-	-
Vanaprabha	R	-	-
Shaktiman	-	R	-
CR 1002	-	-	-
Lunishree	-	-	-
Seema	R	-	-
Sneha	R	-	-
Vandana	-	-	-
Dhala Heera	R	-	-
Radhi	-	-	-
Sonamani	-	-	-
Tapaswini	-	R	-

Pooja	-	-	-
Sarala	-	-	-
Durga	-	-	-
Shatabdi	-	-	-
Anjali	MR	-	-
Hazaridhan	R	MR	-
Sadabahar	-	-	-
Abhishek	R	-	-
Chandrama	R	MR	MR
Virender	MR	-	-
Geetanjali (Aromatic)	-	-	-
Ketekijoha (Aromatic)	-	MR	-
Naveen	R	-	-
Rajalaxmi (Hybrid)	-	-	-
Ajay (Hybrid)	MR	MR	MR
Varshadhan	MR	MR	-
Satya Krishna (CR Dhan 10)	-	-	-
Nua kalajeera	MR	-	-
Nua Dhusara (CR Sugandh Dhan 3)	-	-	-
Chandan (CR Boro dhan 2)	MR	MR	MR
Hanseswari (CR Dhan 70)	MR	-	-
CR Dhan 40	MR	-	-
Swarna Sub1	-	-	-
Sahbhagidhan	-	-	-
Phalguni	R	-	MR
Reeta (CR Dhan 401)	-	-	-
Luna Suvarna (CR Dhan 403)	R	-	-
Luna Sampad (CR Dhan 402)	R	-	-
Nua Chinikamini (Aromatic)	R	-	-
CR Dhan 501	R	-	-
CR Dhan 701	-	-	-
CR Dhan 601	R	-	-
CR Dhan 500	MR	-	-
Satyabhama (CR Dhan 100)	-	-	-
Pyari (CR Dhan 200)	MR	-	-
Hue (CR Dhan 301)	-	-	-
Improved Lalat	-	R	-
Improved Tapaswini	-	R	R
Sumit (CR Dhan 404)	-	-	-
Poorna Bhog (CR Dhan 902)	R	-	-
Jalamani (CR Dhan 503)	MR	-	-

Jayanti Dhan (CR Dhan 502)	MR	-	-
Luna Barial (CR Dhan 406)	MR	-	-
Luna Sankhi (CR Dhan 405)	MR	-	-
CR Dhan 907	R	-	-
CR Dhan 300	MR	-	-
CR Dhan 303	MR	-	-
CR Dhan 305	MR	-	MR
CR Dhan 304	-	-	-
CR Dhan 201	-	-	-
CR Dhan 202	-	-	-
CR Dhan 407	-	-	-
CR Dhan 505	-	-	-
CR Dhan 204	-	-	-
CR Dhan 306 (IET 22084)	-	-	-
CR Dhan 205 (IET 22737)	-	-	-
CR Dhan 101 (Ankit)	-	-	-
CR Dhan 203	-	-	-
CR Dhan 206	-	-	-
CR Dhan 307 (Maudamani)	-	-	-
CR Dhan 408 (Chaka Akhi)	-	MR	-
CR Dhan 310	-	-	-
CR Dhan 207 (Srimati)	-	-	-
CR Dhan 209 (Priya)	-	-	-
CR Dhan 409 (Pradhan Dhan)	-	-	-
CR Dhan 507	-	-	-
CR Dhan 800	-	MR	-
CR Sugandh Dhan 910 (Aromatic)	MR	-	-
CR Dhan 311 (Mukul)	-	-	-
CR Dhan 508	-	-	-
CR Dhan 506	-	-	-
CR Sugandh Dhan 908 (Aromatic)	MR	MR	-
CR Sugandh Dhan 909 (Aromatic)	-	MR	-
Gangavati Ageti (Aromatic)	-	-	-
Purna	-	-	-
CR Dhan 309	-	-	-
CR Dhan 801	MR	MR	-
CR Dhan 802 (Subhas)	-	MR	R
CR Dhan 510	MR	-	-
CR Dhan 511	MR	MR	-
CR Dhan 312	MR	-	-
CR Dhan 313	MR	MR	-
CR Dhan 602	MR	-	-
Santha Bhima (CR Dhan 102)	MR	-	-

Sarumina (CR Dhan 210)	MR	-	-
CR Dhan 410 (Mahamani)	MR	MR	-
CR Dhan 308	MR	MR	-
CR Dhan 314 (Identified by VIC,	MS	-	-
CR Dhan 315	-	-	-
CR Dhan 318 (Identified by VIC,	MR	-	-
CR Dhan 319 (Identified by VIC,	-	-	-
CR Dhan 320 (Identified by VIC,	MR	-	-
CR Dhan 316	-	-	-
CR Dhan 317	-	-	R
CR Dhan 411	-	-	-
CR Dhan 412	-	-	-
CR Dhan 413	-	-	R
CR Dhan 512	-	-	-
CR Dhan 702	MR	-	-
CR Dhan 703	-	MR	-
CR Dhan 803	MR	-	R

The high-yielding rice varieties were developed by the Indian Council of Agricultural Research-National Rice Research Institute, and released by Central Varietal Release Committee. The resistance of rice to blast, leaf blight disease (BLB) and brown planthopper (BPH) was recorded. R: resistant; MR: moderate resistant; MS: moderate susceptible; S: susceptible; - indicates no data.Data was collected from National Rice Research Institute (https://icar-nrri.in/released-varieties/)
